# Synthesis of the [11]Cyclacene Framework by Repetitive Diels–Alder Cycloadditions

**DOI:** 10.3390/molecules26103047

**Published:** 2021-05-20

**Authors:** John B. Bauer, Fatima Diab, Cäcilia Maichle-Mössmer, Hartmut Schubert, Holger F. Bettinger

**Affiliations:** 1 Institut für Organische Chemie, Universität Tübingen, Auf der Morgenstelle 18, 72076 Tübingen, Germany; john.bauer@uni-tuebingen.de (J.B.B.); fatima.diab@web.de (F.D.); 2 Institut für Anorganische Chemie, Universität Tübingen, Auf der Morgenstelle 18, 72076 Tübingen, Germany; Caecilia.Maichle-Moessmer@uni-tuebingen.de (C.M.-M.); hartmut.schubert@uni-tuebingen.de (H.S.)

**Keywords:** Diels–Alder reaction, cyclacene, diastereoselectivity, zigzag hydrocarbon belt, density functional theory, cycloaddition

## Abstract

The Diels–Alder cycloaddition between bisdienes and bisdienophile incorporating the 7-oxa-bicyclo[2.2.1]heptane unit are well known to show high diastereoselectivity that can be exploited for the synthesis of molecular belts. The related bisdiene 5,6,7,8-tetramethylidene-2-bicyclo[2.2.2]octene is a valuable building block for the synthesis of photoprecursors for acenes, but it has not been employed for the synthesis of molecular belts. The present work investigates by computational means the Diels–Alder reaction between these bisdiene building blocks with *syn*-1,4,5,8-tetrahydro-1,4:5,8-diepoxyanthracene, which shows that the diastereoselectivity of the Diels–Alder reaction of the etheno-bridged bisdiene is lower than that of the epoxy-bridged bisdiene. The reaction of the etheno-bridged bisdiene and *syn*-1,4,5,8-tetrahydro-1,4:5,8-diepoxyanthracene in 2:1 ratio yields two diastereomers that differ in the orientation of the oxa and etheno bridges based on NMR and X-ray crystallography. The all-*syn* diastereomer can be transformed into a molecular belt by inter- and intramolecular Diels–Alder reactions with a bifunctional building block. The molecular belt could function as a synthetic intermediate *en route* to a [11]cyclacene photoprecursor.

## 1. Introduction

**Scheme 1:**
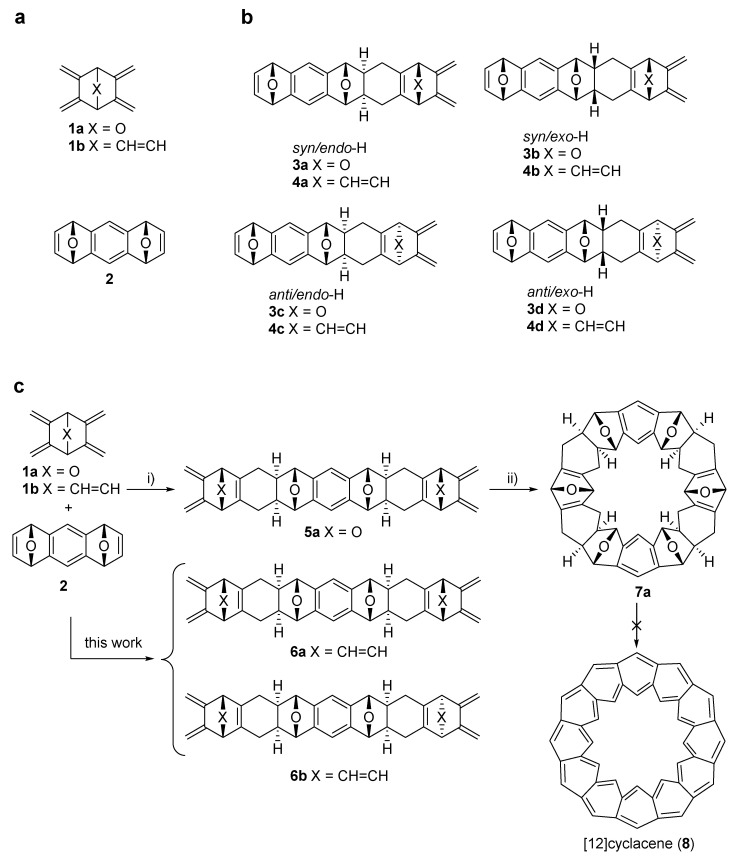
(**a**) Structures of bisdiene **1a**–**b** and bisdienophil **2**; (**b**) cycloaddition of compounds **2** and **1a**,**b** in 1:1 ratio could in principle result in four diastereoisomeric 1:1 adducts **3a**–**d** and **4a**–**d**. For **1a** only isomer **3a** could be detected by Stoddart’s group; (**c**) Diels–Alder reaction between **1** and **2** (2:1 ratio) yields **5a** (from **1a**) or **6a** and **6b** (from **1b**) and synthesis of kohnkene (**7a**), a precursor of [12]cyclacene (**8**), by Stoddart’s group; (i) **1a** + **2**, toluene, reflux, 14 h, 61%; (ii) **5a** + **2**, CH_2_Cl_2_, 10 kbar, 200 h, 55–60 °C, 20%.

The Diels–Alder (DA) reaction developed into a powerful synthetic method since its discovery more than 100 years ago [1,2,3,4,5,6]. As a member of the class of pericyclic reactions, this cycloaddition is particularly valuable due to its stereospecificity and the often observed high diastereoselectivity. This last feature is highly desirable and turned out to be the key to the successful synthesis of molecular belts [7,8,9,10]. In a series of classic papers, Stoddart et al. reported their results of the detailed analysis of the diastereoselectivity of DA reactions involving epoxy bridged bisdiene **1a** and bisepoxy bridged bisdienophile **2** (Scheme 1) [7,11,12,13,14,15].

The DA reaction of a 1:1 mixture of **1a** and **2** exclusively yields the *syn/endo*-H isomer **3a** out of four possible diastereoisomers due to stereoelectronic effects, as discussed by Stoddart et al. (Scheme 1b) [7]. We follow their nomenclature and use the descriptors *syn* and *anti* for the relative configurations of the endoxide bridges and *exo* and *endo* for the relative configurations of the hydrogen atoms of the bisdiene after DA reaction.

As a consequence of the pronounced diastereoselectivity of the DA reaction involving strained bisdiene **1a** and bisdienophile **2**, the reaction in 2:1 ratio also yields a single diastereomer of the 2:1 adduct **5a** (Scheme 1c). Remarkably, the reaction in 1:1 ratio does not produce polymers since the reactivity of the second diene unit is reduced once the first diene unit has undergone a DA reaction, as discussed by Stoddart et al. [7]. Due to the diminished reactivity, the reaction with another bisdienophile building block requires high pressure (Scheme 1c). In this way, it was possible to synthesise molecular belt **7a**, a potential precursor of [12]cyclacene (**8**) (Scheme 1c).

First envisioned by Heilbronner [16], fully conjugated molecular belts consisting solely of linearly fused benzene rings, cyclacenes ([n]CA, where n is the number of benzene rings), have not yet been synthesised although related systems have been achieved, for example, by Gleiter et al. [17,18] and by Itami et al. [19]. Seminal work by Stoddart [7,11,12,13,14,15], Schlüter [9,20], and Corey [8,21] demonstrated the significant resistance of the belts toward full conjugation. More recent attempts by on-surface synthesis using a tetraepoxy precursor further demonstrated the challenge in achieving full conjugation [10]. Synthesis of conjugated molecular belts and structurally related molecules has been reviewed in the literature [22,23,24,25,26]. Computational analyses of the strain energies arrive at values around 123 kcal mol^−1^ for [10]CA and 103 kcal mol^−1^ for [12]CA, indicating that the final reaction step in a CA synthesis will result in a substantial energy penalty due to the buildup of strain [27,28,29].

The resistance of cyclacene formation also under the conditions of on-surface synthesis is remarkable as the combination of ultrahigh vacuum, submonolayer coverage, and catalytically active single crystal transition metal surface is employed. These conditions previously allowed the production of acenes [30,31,32,33,34], the highly reactive linear analogues of cyclacenes [35]. Acenes could be obtained up to undecacene under matrix isolation conditions by photocleavage of α-diketone bridges. These were installed synthetically by DA reactions involving bisdiene **1b** that is closely related to **1a** employed by Stoddart. If this shows similar diastereoselectivity, then it should be possible to employ it in the synthesis of molecular belts as well. However, the high diastereoselectivity of **1a** is ascribed to the distortion of the diene unit from planarity, which causes the ‘lower’ side to be more reactive, thus favoring the formation of *syn* diastereomers [7]. The larger etheno bridge of **1b** causes less distortion of the diene moieties according to single-crystal X-ray crystallography [36] and thus reduced diastereoselectivity is anticipated.

Indeed, Wegener and Müllen reported that the DA reaction of bisdiene **1b** with the closely related monodienophile 1,4-dihydro-1,4-epoxynaphthalene gave two diastereomers in a ratio of 4:1 under high-pressure conditions [37,38]. The major isomer was identified as having all bridges *syn* standing with hydrogen atoms at the newly formed bonds in *endo* position with respect to the epoxy bridge. The minor isomer had the corresponding H atoms also in *endo* position, but one epoxy bridge was *anti* to the other bridges, as evidenced by X-ray crystallography [37,38]. This observation confirms the reduced diastereoselectivity in DA reactions of bisdiene **1b** and bisdienophile **2** under high-pressure conditions. The reaction did not result in cyclic materials, according to Müllen, but in polymeric materials for which elucidation of the stereochemistry was impossible [37,38].

We here report a computational analysis of the DA reactions involving bisdienes **1a** and **1b** with bisdienophile **2** that confirm the reduced diastereoselectivity in their Diels–Alder reaction. We demonstrate that diastereomer **6a** can be obtained from **1b** and **2** (Scheme 1c) with sufficient diastereomeric excess to employ it in the synthesis of a molecular belt, as described in the second part of this paper.

## 2. Results and Discussion

### 2.1. Computations

The computational analysis employed the M062X functional and the 6-311+G** basis set. The effects of the solvent toluene were taken into account with a polarisable continuum model for geometry optimisation and subsequent computation of harmonic vibrational frequencies (see SI for comparison with other computational methods and Tables S6 and S7 for energy data). The Diels–Alder reaction between the bisdienes **1** and the bisdienophile **2** in a 1:1 ratio can result in four diastereomeric products **3a**–**d** (from **1a**) and **4a**–**d** (from **1b**) (see Scheme 1b).

The *syn/endo*-H isomer **3a**, the sole reaction product observed by Ashton et al. [7], is the least stable diastereomer, while the *anti/exo*-H isomer **3d** is the most stable one. The energy difference (ΔΔG°(298.15 K) = 1.9 kcal mol^−1^) between these isomers is small. Among the diastereomers, **4** the *syn/endo*-H product **4a** is the most stable one, while the *syn/exo*-H isomer **4b** is the least stable. The free energy difference between these isomers, ΔΔG°(298.15 K) = 0.9 kcal mol^−1^, is smaller than in the case of **3**.

**Figure 1:**
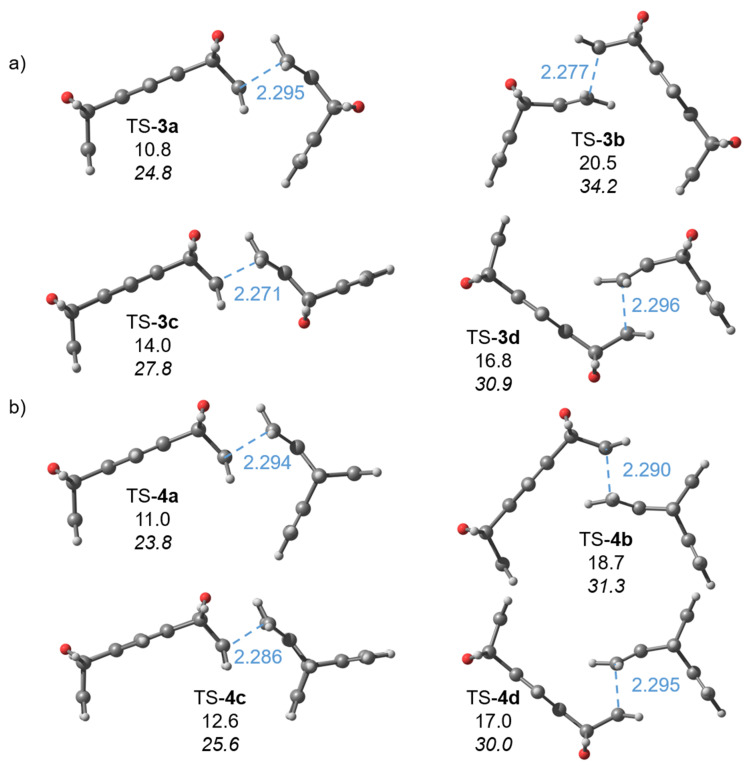
Lowest energy transition states for the formation of diastereomers **3** (**a**) and **4** (**b**) as computed at the M062X/6-311+G**/toluene level of theory. Distances between reacting carbon atoms are given in Å (blue), enthalpies (normal print), and free energies (italics) at 298.15 K are given in kcal mol^−1^. Note that for the formation of each diastereomer, there is another transition state that is higher in energy and that is not depicted for clarity: Red, oxygen; dark gray, carbon; light gray, hydrogen.

Since two transition states (TS) are conceivable for the formation of each diastereomer, eight TS each are relevant for the formation of **3** and **4**. The expected TS could each be located on the corresponding potential energy surfaces. Among diastereomers **3**, the least stable 3**a** is formed with the lowest energy barrier (ΔG^‡^ = 24.8 kcal mol^−1^) (Figure 1). The second-lowest barrier (ΔG^‡^ = 27.8 kcal mol^−1^) is associated with the formation of the *anti/endo*-H isomer **3c**. Most importantly, the energy difference between the lowest and the second-lowest barrier (formation of **3a** and **3c**) is ΔΔG^‡^ = 3.0 kcal mol^−1^. The computations also show that the subsequent DA reaction of the *syn/endo*-H adduct **3a**, with its remaining dienophile and a second equivalent of bisdiene in a *syn*/*endo*-H fashion to give **5** has almost the same barrier (ΔG^‡^ = 24.7 kcal mol^−1^) as the first *syn*/*endo* DA reaction. The large energetic preference of the *syn*/*endo*-H cycloaddition mode is in accord with the observation of the diastereoselective DA reaction between **1a** and **2** by Ashton et al. [7].

The desired *syn/endo*-H isomer **4a** and the *anti/endo*-H isomer **4c** are almost isoenergetic. As a consequence, the barriers of their formations are more much more similar, although the formation of the isomer **4a** still is preferred by ΔΔG^‡^ = 1.9 kcal mol^−1^. The barrier height for the formation of **4a** is lower than that of **3a** by 1.0 kcal mol^−1^, indicating that the endoxide bridge of **1a** reduces reactivity. Formation of the *syn/exo*-H (**4b**) and *anti/exo*-H (**4d**) isomers are associated with barriers that are higher than that for **4a** by 7.5 kcal mol^−1^ and 6.2 kcal mol^−1^, respectively, which excludes the corresponding stereochemical modes of reaction. As in the case of **3a**, the subsequent DA reaction of **4a** with the second equivalent of bisdiene **1b** has a similar barrier to the first one for both the *syn*/*endo* (ΔG^‡^ = 24.3 kcal mol^−1^ to give **6a**) and the *anti*/*endo* (ΔG^‡^ = 25.8 kcal mol^−1^ to give **6b**) fashion (see Figure S65). In consequence, the computations suggest that the 2:1 DA reaction will likely give a mixture of twofold *syn*/*endo* and *syn*/*endo* plus *anti*/*endo* diastereoselectivity.

To summarise, the results of the computations of the DA reactions involving **1a** and **2** are in full accord with the observations of Ashton et al. [7] regarding the high diastereoselectivity of the DA reaction. The computations suggest that the diastereoselectivity is reduced in the DA reaction of **1b** and **2**, in accord with the reports by Wegener and Müllen [37,38]. The formation of a molecular belt was not observed by them, but we reason that it should be possible to achieve if the proper diastereomer **6a** can be isolated and employed in subsequent DA reactions.

### 2.2. Synthesis

**Scheme 2:**
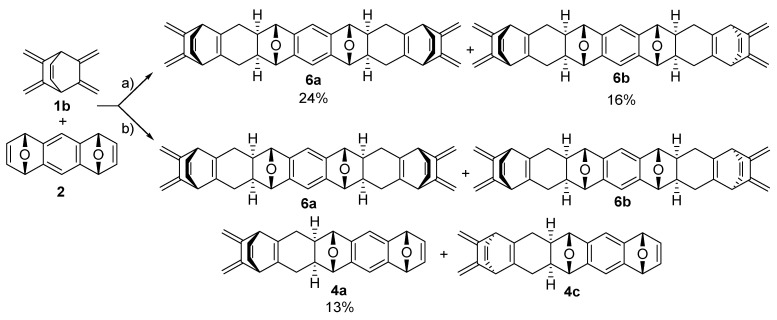
Diels–Alder reaction of **1b** and **2**. Reaction conditions and isolated yields (**a**) 2.2 equivalent of **1b**, 1 equivalent of **2**, toluene, reflux, 18 h; (**b**) equimolar ratio of **1b** and **2**, toluene, reflux, 18 h. **4c** could not be isolated.

The required bisdiene **1b** and bisdienophile **2** were synthesised, as reported in [7,39]. The DA reaction of **2** with 2.2 equivalents of **1b** in boiling toluene resulted in the formation of isomers **6a** and **6b** (Scheme 2). Integration of aromatic protons of the crude product reveals the ratio of diastereomers **6a**:**6b** to be 1.9:1.

**Figure 2:**
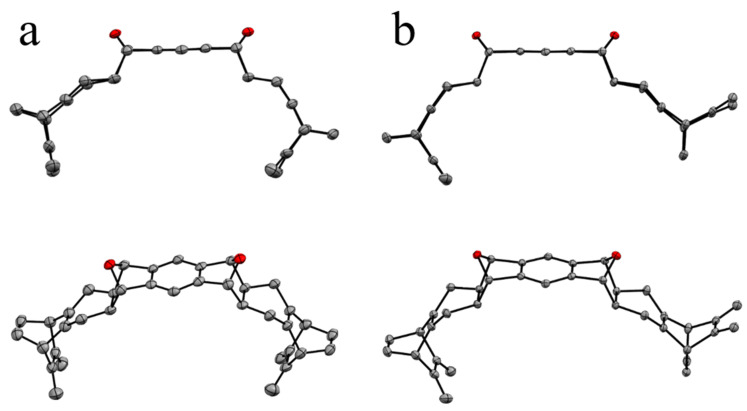
X-ray molecular structures of (**a**) **6a** and (**b**) **6b** (50% thermal ellipsoids; side views (**top**) and perspective views (**bottom**); hydrogen atoms are removed for clarity.

The stereochemistry of isomers **6a** and **6b** was determined by NMR spectroscopy and single-crystal X-ray crystallography. According to the Karplus equation, the coupling constant with a dihedral angle around 90° is close to zero, compared to dihedral angles of 0° and 180°. For the *exo*-H-isomers, the strong coupling would be expected, as opposed to no or weak coupling in the *endo*-H-isomers. In the COSY NMR spectra of **6a** (Figure S5) and **6b** (Figure S11), no coupling can be observed for the corresponding atoms. The NOESY NMR spectra, however, show an interaction between the corresponding atoms (for **6a**: Figure S6, for **6b**: Figure S12). An assignment of the ^1^H and ^13^C NMR signals to the corresponding atoms of **6a** (Table S1) and **6b** (Table S2) and the computed dihedral angles between the corresponding protons for the four diastereomers **4a**–**d** (Figure S21) can be found in the Supplementary Materials. The conclusions based on NMR spectroscopy were confirmed by single-crystal X-ray crystallography (Figure 2).

The reaction of an equimolar ratio of compounds **2** and **1b** leads to the formation of both 2:1 adducts **6a** and **6b,** and additionally, to two 1:1 adducts under these conditions (Scheme 2). The COSY NMR spectra (Figure S22) of the mixture of 1:1 adducts do not show any coupling for the corresponding atoms, which indicates the formation of the *endo*-H isomers. One 1:1 adduct could be isolated through preparative chromatography. Based on the preferred *syn*/*endo*-H diastereoselectivity, it is assigned as **4a**. The minor 1:1 adduct, likely isomer **4c**, could not be isolated, but the separation of **4c** can be achieved with HPLC–MS chromatography where the [M + H]^+^ and [M + H-H_2_O]^+^ ions can be detected (see Figure S63).

**Scheme 3:**
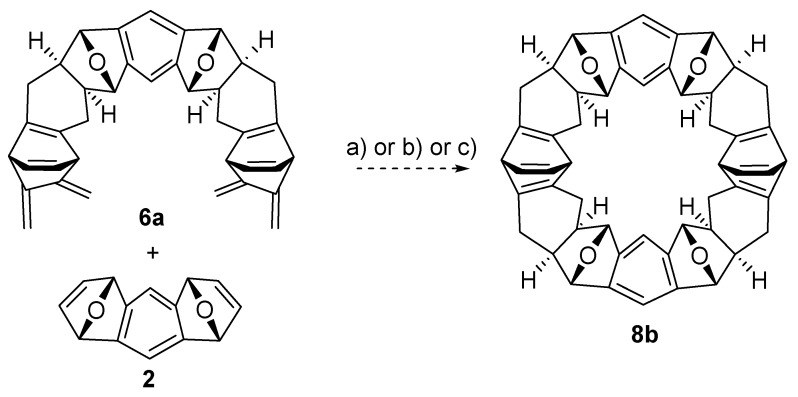
(**a**) Decalin, 150 °C, 18 h; (**b**) microwave: DCM, BMIM–PF_6_, 140 °C, 3 h, 14 bar; (**c**) autoclave: toluene, 120 °C, 168 h, 85 bar.

Diels–Alder reaction of compounds **6a** and **2** could in principle lead to macrocycle **8b**. After employing three different reaction conditions (Scheme 3), MS measurements of all crude reaction mixtures could detect *m*/*z* of 733.1, 715.4, 697.4, and 679.4, which could result from the ions [M + H]^+^, [M-H_2_O + H]^+^, [M-2H_2_O + H]^+^, and [M-3H_2_O + H]^+^. Not enough product could be gained for NMR experiments. After heating **2** and **6a** for 168 h at 120 °C and 85 bar the chromatogram of the crude product shows 2 signals at 35.16 min and 36.20 for the *m*/*z* of 733.1, 715.4, 697.4, and 679.4 (Figure S64), indicating a rearrangement of **8b**. It appears that the higher temperatures employed in this reaction, compared to those for the synthesis of **4a**, resulted in significant thermal degradation. Unfortunately, we were not able to find milder conditions for this transformation to proceed in better yields.

**Scheme 4:**
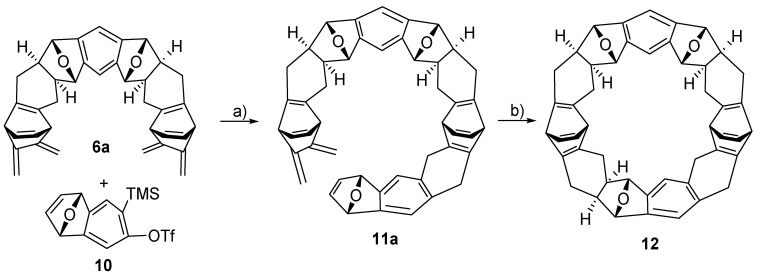
Synthesis of macrocycle **12**: (**a**) KF, 18-crown-6, DCM, MeCN, RT, 18 h; (**b**) toluene, 75 °C, 5 h. Overall yield from **6a**: 7%.

**Scheme 5:**
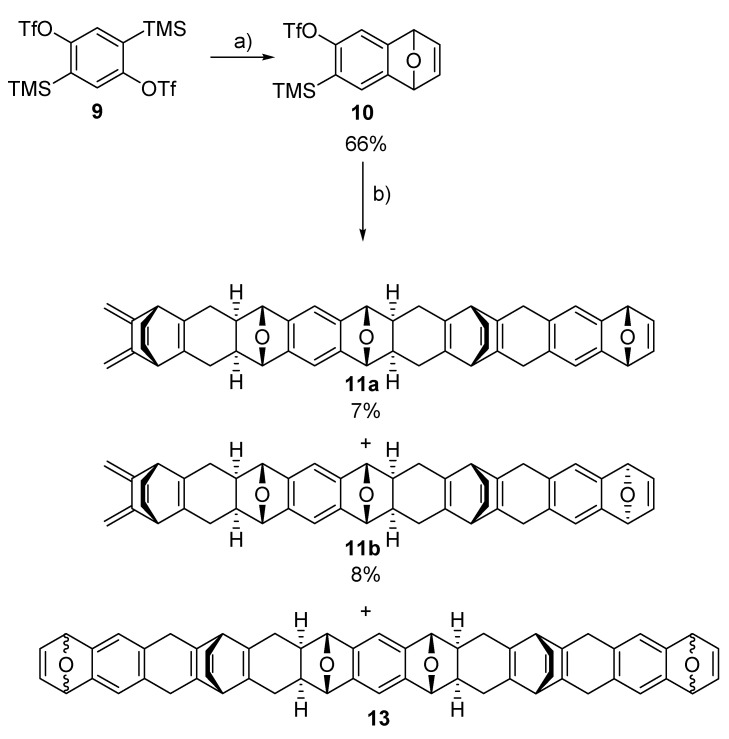
(**a**) Furan, CsF, MeCN, 45 °C, 1 h; (**b**) **6a**, KF, 18-crown-6, DCM, MeCN, 18 h RT.

We, therefore, sought a different way to construct a molecular belt. Ashton et al. [7] showed that the final DA reaction can proceed under mild conditions as it is an intramolecular reaction. We thus resorted to a more reactive dienophile than **2** willing to sacrifice the high diastereoselectivity. For this purpose, we chose an aryne that we generated from **10**. Macrocycle **12** was obtained from **6a** and **10** in an overall yield of 7% upon heating a mixture of **6a** and **10** with a fluoride source, which generates the aryne from **10** to react with **6a** in a Diels–Alder reaction to afford compound **11a** (Scheme 4). This in turn undergoes an intramolecular Diels–Alder reaction to yield **12**. Compound **10** was obtained in a yield of 66% after treating commercially available **9** with furan and CsF in MeCN for 1 h at 45 °C (Scheme 5).

The products **11a** and **11b** could be obtained and characterised after treatment of **6a** and **10** with KF and 18-crown-6 for 24 h at room temperature. All the ether and olefin bridges in the target molecule **11a** are *syn* to each other. Additional side products were formed during the reaction, which were more polar than **11a** and **11b**, and were not isolated or characterised. The formation of 2:1 adducts **13**, with different orientations of the newly installed endoxide bridges would be expected.

Since compounds **11a** and **11b** could not be differentiated by NMR and MS, both compounds were heated in boiling toluene for 2 h, respectively, with **11a** affording **12** as product and **11b** remaining unchanged. This indicates that **11a** has all the bridges on the same side. The chemical transformation from **11a** to **12** can be proven by the changes in the ^1^H and ^13^C NMR spectra. For C_S_ symmetric compound **11a** all the required ^13^C signals can be observed, but due to the very similar chemical shifts of the bridgehead carbon atoms (C-8) and (C-12), their assignment is interchangeable (Table 1). Likewise, the diastereotopic hydrogen atoms at (C-6) and (C-14) can only be assigned pairwise (Figure S49). The same is true for a number of ^13^C and ^1^H signals of **12**, but the required amount of ^13^C resonances for C_S_ symmetric **12** is observed (Table 1). The terminal carbon of the diene of **11a** can easily be identified with a DEPT-135 measurement, which shows a negative signal at δ_C_ 101.2 (C-1). After the intramolecular DA reaction to **12**, the signal is shifted to δ_C_ 31.2 (C-1). The methylene protons of the diene unit of **11a** at δ_H_ 4.86 and 4.70 (H-1) are transformed to diastereotopic protons in **12**, which can be found in the range between 2.5 and 2.0 ppm (H-1) (Figure S50). The protons for the dienophile at δ_H_ 6.91 (H-24) in **11a** are shifted to the range between 1.36 and 1.20 ppm (H-24) in **12**. Analogously a shift takes place from δ_C_ 143.0 (C-24) to δ_C_ 46.3/45.8 (C-24). The assignment to **11a** and **12** are in agreement with the 2D NMR and DEPT spectra and with the high-resolution APCI measurements.

### 2.3. Properties of Macrocycle ***12***

**Figure 3:**
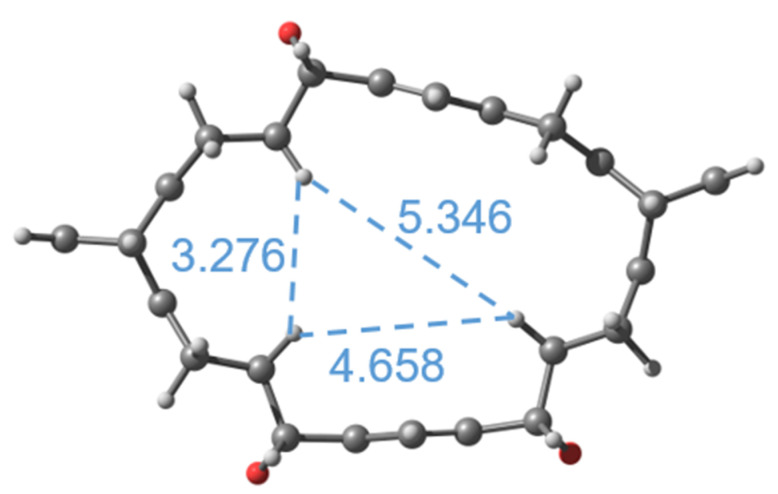
Molecular structure of macrocycle **12** as computed at the M062X/6-311+G**/toluene level of theory. Distances between *endo*-H atoms are given in Å (blue): Red, oxygen; dark gray, carbon; light gray, hydrogen.

Since we were not able to obtain single crystals suitable for X-ray crystallography, we computed the structure of **12** (Figure 3). The distances between the internal *endo* hydrogen atoms range between 3.3–5.3 Å. The molecular belt **12** is significantly more stable than precursor **11a** as indicated by the heat of reaction for cyclisation, ΔH°(298.15 K) = −42.5 kcal mol^−1^.

The molecular belt **12** decomposes both in toluene solution during heating within a few hours and in substance during storage in a refrigerator within a few weeks to unidentified products.

## 3. Materials and Methods

### 3.1. General Methods

All commercially available reagents were used as received. Air and/or water-sensitive reactions were carried out under N_2_ in oven-dried glassware. Solvents for chromatography and workup including diethyl ether (Et_2_O), ethyl acetate (EtOAc), hexane, methanol, and dichloromethane (DCM) were purchased as high-performance liquid chromatography (Sigma-Aldrich, Steinheim, Germany) grade. DCM and toluene used for the synthetic reactions were purified by an SPS system (MBraun MB-SPS-800). Acetonitrile and *n*-BuLi (1.6 M in hexane) used in the synthetic reactions were bought anhydrous and under an inert atmosphere from Sigma-Aldrich (Steinheim, Germany). KF was dried under vacuum at 150 °C for 1.5 h. For deactivated aluminium oxide for column chromatography, 8 g of water was added to 100 g of activated aluminium oxide. Compound **1b** was synthesised according to the reported literature and showed the same spectroscopic properties as reported [39]. Thin-layer chromatography (TLC) was performed on silica TLC plates (Polygram Sil G/UV, Macherey-Nagel, Düren, Germany) and visualised by illumination with a UV lamp (Analytikjena, Jena, Germany) 254 and 365 nm wavelength). TLC plates were stained with phosphomolybdic acid, 10 wt% solution in ethanol. Silica gel (Macherey-Nagel, 0.04–0.063 mm) was used as received. All NMR spectra were recorded at 298 K on 400 and 700 MHz Bruker Avance III spectrometers. ^1^H and ^13^C shifts were referenced to the residual solvent signal of CDCl_3_, ^1^H-NMR: δ = 7.26 ppm, ^13^C NMR: δ = 77.16 ppm [40]. ^1^H-NMR gave signals at 1.52 (water), 1.26 (grease), and 0.08 (grease) ppm. ^13^C NMR gave signals at 1.2 (grease) ppm [40]. Mass spectrometry measurements were recorded on a maXis 4G-UHR-TOF Bruker Daltonics spectrometer using the electrospray ionisation (ESI) or the atmospheric-pressure chemical ionisation (APCI) technique; the chromatogram in Figure S63 was obtained on an Esquire 3000 plus Bruker Daltonics spectrometer with an Agilent 1100 Series HPLC using linear-gradient conditions: from 90% A (H_2_O/0.1% formic acid) and 10% B (MeOH/0.1% formic acid) to 0% A and 100% B in 20 min, then keeping 0% A and 100% B for 10 min. The flow rate was 0.3 mL/min with eluent monitoring at 254 nm. Column: Macherey-Nagel EC 125/4 Nucleosil 100-5 C18. The chromatogram in Figure S64 was obtained on an amaZon SL Bruker Daltonics spectrometer with an Agilent 1260 Series HPLC using linear-gradient conditions: from 60% A (H_2_O/0.1% formic acid) and 40% B (MeOH/0.1% formic acid) to 5% A and 95% B in 30 min, then keeping 5% A and 95% B for 20 min. The flow rate was 0.3 mL/min with eluent monitoring at 254 nm. Column: Agilent InfinityLab Poroshell 120 EC-C18 4.6 × 100 mm 2.7 Micron with Column ID.

### 3.2. Synthetic Procedures

*rel*-(1*R*,4*S*,5*R*,8*S*)-1,4,5,8-Tetrahydro-1,4:5,8-diepoxyanthracene (**2**)

A solution of 36.6 mL (500 mmol) furan, 10 g (25.4 mmol) 1,2,4,5-tetrabromobenzene in 400 mL of toluene was cooled to −25 °C under argon and a solution of 33.3 mL *n*-butyllithium (1.6 M in hexane) was added dropwise within 1 h. After the addition, the reaction mixture was stirred at room temperature for 12 h. Then, 12.5 mL of methanol was added and the reaction mixture was washed three times with 50 mL of water and three times with 50 mL of saturated sodium chloride solution. After drying over MgSO_4_, the solvent was removed under reduced pressure. The residue was purified by column chromatography (aluminium oxide deactivated, neutral; hexane/DCM/Et_2_O 1:2:1). Product **2** was obtained as a yellow solid. Yield: 1.15 g (5.47 mmol, 21%). **^1^H-NMR** (400 MHz, CDCl_3_) δ [ppm]: 7.19 (2H, s), 7.04-7.02 (4H, apparent t, *J* = 0.96 Hz), 5.63 (4H, broad s). **^13^C-NMR** (100 MHz, CDCl_3_) δ [ppm]: 147.7, 143.5, 113.8, 82.5. **R_F_** (aluminium oxide, hexane/DCM/Et_2_O 1:2:1): 0.35. Spectral data are in accordance with literature information [7].

*rel*-(1*R*,4*S*,5a*S*,6*S*,8*R*,8a*R*,10*R*,13*S*,14a*S*,15*S*,17*R*,17a*R*)-2,3,11,12-Tetramethylene-1,4,5,5a,6,8,8a,9,10,13,14,14a,15,17,17a,18-hexadecahydro-6,17:8,15-diepoxy-1,4:10,13-diethenoheptacene (**6a**) and *rel*-(1*R*,4*S*,5a*R*,6*R*,8*S*,8a*S*,10*S*,13*R*,14a*R*,15*R*,17*S*,17a*S*)-2,3,11,12-tetramethylene-1,4,5,5a,6,8,8a,9,10,13,14,14a,15,17,17a,18-hexadecahydro-6,17:8,15-diepoxy-1,4:10,13-diethenoheptacene (**6b**)

A solution of 150 mg (0.71 mmol) bisdienophile **2** and 265 mg (1.70 mmol) bisdiene **1b** in 70 mL anhydrous toluene was heated under reflux for 18 h. After removing the solvent under reduced pressure, the residue was purified by column chromatography (silica; hexane/DCM 18:1). Both products were obtained as colourless solids. Yield: for **6a**: 90 mg (0.17 mmol, 24%), for **6b**: 60 mg (0.12 mmol, 16%). **6a: ^1^H-NMR** (400 MHz, CDCl_3_) δ [ppm]: 6.98 (2H, s), 6.41-6.37 (4H, m), 4.94 (4H, s), 4.89 (4H, s), 4.75 (4H, s), 3.92–3.88 (4H, m), 2.64–2.56 (4H, m), 2.22–2.14 (4H, m), 1.90–1.83 (4H, m). **^13^C-NMR** (100 MHz, CDCl_3_) δ [ppm]: 144.8, 144.4, 137.7, 133.8, 110.3, 101.3, 85.2, 53.7, 43.5, 30.8. **R_F_** (silica, DCM:EtOAc 18:1): 0.47. **HRMS** (ESI) *m*/*z*: [M + Na]^+^ calcd for C_38_H_34_NaO_2_^+^ 545.24510; found 545.24537. **6b: ^1^H-NMR** (400 MHz, CDCl_3_) δ [ppm]: 7.00 (2H, s), 6.41–6.36 (4H, m), 5.04 (2H, s), 4.94 (2H, s), 4.90 (2H, s), 4.89 (2H, s), 4.80 (2H, s), 4.75 (2H, s), 3.92–3.85 (4H, m), 2.65-2.55 (4H, m), 2.24–2.11 (4H, m), 1.94–1.86 (4H, m). **^13^C-NMR** (100 MHz, CDCl_3_) δ [ppm]: 144.8, 144.6, 144.4, 144.3, 137.7, 136.8, 133.9, 133.8, 110.3, 101.6, 101.3, 85.2, 85.2, 53.6, 53.5, 43.3, 41.4, 30.8, 30.1. **R_F_** (silica, DCM:EtOAc 18:1): 0.53. **HRMS** (ESI) *m*/*z*: [M + Na]^+^ calcd for C_38_H_34_NaO_2_^+^ 545.24510; found 545.24542.

*rel*-(1*R*,4*S*,6*R*,6a*R*,8*R*,11*S*,12a*S*,13*S*)-16,17-Dimethylene-1,4,6,6a,7,8,11,12,12a,13-decahydro-1,4:6,13-diepoxy-8,11-ethanopentacene (**4a**)

A solution of 100 mg (0.48 mmol) bisdienophile **2** and 74 mg (0.48 mmol) bisdiene **1b** in 60 mL anhydrous toluene was heated under reflux for 18 h. After removing the solvent under reduced pressure, the residue was purified by column chromatography (silica; DCM:EtOAc 25:1). The product **4a** was obtained as a colourless solid. Yield: 22 mg (0.06 mmol, 13%). **^1^H-NMR** (400 MHz, CDCl_3_) δ [ppm]: 7.07 (2H, s), 7.01–6.99 (2H, m), 6.42–6.38 (2H, m), 5.63 (2H, s), 4.94 (2H, s), 4.88 (2H, s), 4.75 (2H, s), 3.93–3.89 (2H, m), 2.65–2.56 (2H, m), 2.23–2.14 (2H, m), 1.94–1.87 (2H, m). **^13^C-NMR** (100 MHz, CDCl_3_) δ [ppm]: 148.7, 144.4, 143.5, 143.4, 137.7, 133.7, 112.1, 101.3, 85.0, 82.5, 53.6, 43.4, 30.7. **R_F_** (silica, DCM:EtOAc 18:1): 0.34. **HRMS** (ESI) *m*/*z*: [M + Na]^+^ calcd for C_26_H_22_NaO_2_^+^ 389.15120; found 389.15130.

7-(Trimethylsilyl)-1,4-dihydro-1,4-epoxynaphthalen-6-yl-trifluoromethanesulfonate (**10**)

A solution of 52 mg (0.34 mmol) CsF was added over a solution of 120 mg (0.23 mmol) bistriflate **9** and 33 μL (0.46 mmol) furan in 6 mL of dry MeCN. The mixture was stirred for 1 h at 45 °C. After the evaporation of the solvent under reduced pressure, the residue was purified by column chromatography (silica, hexane:EtOAc 7:3). The product **10** was obtained as a colourless solid. Yield: 56 mg (0.15 mmol, 66%) **^1^H-NMR** (700 MHz, CDCl_3_) δ [ppm]: 7.33 (1H, s), 7.24 (1H, s), 7.07-7.02 (2H, m), 5.72 (2H, broad s), 0.34 (9H, s). **^13^C-NMR** (176 MHz, CDCl_3_) δ [ppm]: 153.9, 152.5, 148.2, 143.4, 142.7, 128.6, 126.2, 121.3, 119.5, 117.7, 115.9, 113.2, 82.4, 82.1, −0.6. **R_F_** (silica, hexane:EtOAc 7:3): 0.72. Spectral data are in accordance with literature information [41].

*rel*-(1*R*,4*S*,7*R*,8a*S*,9*S*,11*R*,11a*R*,13*R*,16*S*,17a*S*,18*S*,20*S*,20a*R*,22*S*)-14,15-Dimethylene-1,4,6,7,8,8a,9,11,11a,12,13,16,17,17a,18,20,20a,21,22,23-icosahydro-1,4:9:20,11:18-triepoxy-7,22:13,16-diethenodecacene (**11a**) and *rel*-(1*R*,4*S*,7*S*,8a*R*,9*R*,11*S*,11a*S*,13*S*,16*R*,17a*R*,18*R*,20*R*,20a*S*,22*R*)-14,15-dimethylene-1,4,6,7,8,8a,9,11,11a,12,13,16,17,17a,18,20,20a,21,22,23-icosahydro-1,4:9:20,11:18-triepoxy-7,22:13,16-diethenodecacene (**11b**)

A suspension of 15 mg (0.26 mmol) KF and 63 mg (0.24 mmol) 18-crown-6 in dry MeCN was cooled to 0 °C and a solution of 83 mg (0.16 mmol) diene **6a** and 58 mg (0.16 mmol) triflate **10** in 10 mL of dry DCM was added dropwise within 10 min. The reaction mixture was stirred for 1 h at 0 °C and then at room temperature for 18 h. After the evaporation of the solvent under reduced pressure, the residue was purified by column chromatography (silica, DCM:EtOAc 25:1). Both products were obtained as colourless solids. Yield: for **11a**: 7 mg (0.011 mmol, 7%), for **11b**: 8 mg (0.012 mmol, 8%). **11a: ^1^H-NMR** (700 MHz, CDCl_3_) δ [ppm]: 6.95 (2H, s), 6.93 (2H, s), 6.91 (2H, s), 6.77–6.75 (2H, m), 6.39–6.37 (2H, m), 5.61 (2H, s), 4.87 (2H, s), 4.86 (2H, s), 4.85 (2H, s), 4.70 (2H, s), 4.15–4.13 (2H, m), 3.88–3.86 (2H, m), 3.49–3.33 (4H, m), 2.70–2.65 (2H, m), 2.60–2.55 (2H, m), 2.18–2.12 (2H, m), 2.12–2.06 (2H, m), 1.92–1.87 (2H, m), 1.77–1.71 (2H, m). **^13^C-NMR** (176 MHz, CDCl_3_) δ [ppm]: 146.6, 144.8, 144.7, 144.4, 143.8, 143.0, 140.8, 139.5, 137.5, 133.8, 130.7, 120.9, 110.2, 101.2, 85.2, 85.2, 82.2, 55.3, 53.6, 43.1, 42.9, 33.7, 31.4, 30.7. **R_F_** (silica, DCM:EtOAc 18:1): 0.36. **HRMS** (APCI) *m*/*z*: [M + H]^+^ calcd for C_48_H_41_O_3_^+^ 665.30502; found 665.30606. **11b: ^1^H-NMR** (700 MHz, CDCl_3_) δ [ppm]: 6.97 (2H, s), 6.93 (2H, s), 6.93 (2H, s), 6.74–6.72 (2H, m), 6.39–6.36 (2H, m), 5.60 (2H, s), 4.89 (2H, s), 4.87 (2H, s), 4.86 (2H, s), 4.70 (2H, s), 4.14-4.12 (2H, m), 3.88–3.86 (2H, m), 3.43–3.37 (4H, m), 2.73–2.68 (2H, m), 2.60–2.56 (2H, m), 2.19–2.12 (2H, m), 2.12–2.05 (2H, m), 1.90–1.83 (4H, m). **^13^C-NMR** (176 MHz, CDCl_3_) δ [ppm]: 146.6, 144.7 (2 isochronous nuclei), 144.3, 143.5, 142.9, 140.7, 139.6, 137.6, 133.8, 130.7, 120.9, 110.2, 101.4, 85.3, 85.2, 82.2, 55.3, 53.6, 43.3, 42.5, 33.7, 31.4, 30.8. **R_F_** (silica, DCM:EtOAc 18:1): 0.39. **HRMS** (APCI) *m*/*z*: [M + H]^+^ calcd for C_48_H_41_O_3_^+^ 665.30502; found 665.30625.

Macrocycle **12**

A solution of 7 mg (0.011 mmol) **11a** in 5 mL toluene was stirred for 5 h at 75 °C. After the evaporation of the solvent under reduced pressure, the residue was purified by column chromatography (silica, DCM:EtOAc 25:1). The product **12** was obtained as a colourless solid. The yield could not be determined reliably due to the small reaction scale but is estimated to be around 80 to 90%. **^1^H-NMR** (700 MHz, CDCl_3_) δ [ppm]: 6.85 (2H, s), 6.82–6.80 (2H, m), 6.77 (2H, s), 6.75–6.73 (2H, m), 4.82 (2H, s), 4.81 (2H, s), 4.79 (2H, s), 4.23–4.21 (2H, m), 4.14–4.12 (2H, m), 3.53–3.46 (2H, m), 3.26–3.19 (2H, m), 2.57–2.48 (6H, m), 2.23–2.17 (2H, m), 2.20–2.04 (4H, m), 1.36–1.31 (2H, m), 1.31–1.25 (2H, m), 1.25–1.20 (2H, m). **^13^C-NMR** (176 MHz, CDCl_3_) δ [ppm]: 147.8, 147.5, 147.4, 145.2, 145.1 143.7, 143.3, 139.8, 139.0, 133.5, 118.8, 110.0, 84.3, 84.1, 84.0, 57.0, 56.3, 47.2, 46.3, 45.8, 34.3, 31.7, 31.2, 31.2. **R_F_** (silica, DCM:EtOAc 18:1): 0.34. **HRMS** (APCI) *m*/*z*: [M + H]^+^ calcd for C_48_H_41_O_3_^+^ 665.30502; found 665.30521.

### 3.3. X-ray Crystallographic Data

Suitable crystals for **6b** were grown from a solution of chloroform and toluene at 5 °C. Data were collected on a Bruker SMART APEX II instrument equipped with a fine focus sealed tube and TRIUMPH monochromator using MoK_α_ radiation (λ = 0.71073 Å). The data collection strategy was determined using COSMO [42] employing ω- and ϕ scans. Raw data were processed using APEX [43] and SAINT [44] corrections for absorption effects were applied using SADABS [45].

Suitable crystals for **6a** were grown from a solution of dichloromethane and hexane at room temperature. Data collections were therefore performed on a Bruker APEX DUO instrument equipped with an IμS microfocus sealed tube and QUAZAR optics for MoK_α_ radiation (λ = 0.71073 Å). The Data collection strategy was determined using COSMO [42] employing ω- scans. Raw data were processed using APEX [46] and SAINT [47], corrections for absorption effects were also applied using SADABS [45].

The structures were solved by direct methods and refined against all data by full-matrix least-squares methods on F^2^ using SHELXTL [48] and Shelxle [49]. All atoms (except hydrogen) were refined anisotropically. All methyl groups were refined as rigid and rotating (difference Fourier density optimisation) CH_3_ groups with *U*_iso(H)_ = 1.5*U*_eq(C)_ and all other hydrogen atoms were placed in calculated positions and refined using a riding model with *U*_iso(H)_ = 1.2*U*_eq(C)_ for aromatic and *U*_iso(H)_ = 1.5*U*_eq(C)_ for all other hydrogen atoms.

Crystal Data for C_38_H_34_O_2_ **6b** (M = 522.65 g/mol): monoclinic, space group P2/c (no. 13), a = 14.0261(2)Å, b = 6.81100(10) Å, c = 28.6971(5) Å, β = 95.4760(10)°, V = 2728.97(7) Å^3^, Z = 4, T = 100(2) K, μ(MoK_α_) = 0.077 mm^−1^, D_calc_ = 1.272 g/cm^3^, 30,286 reflections measured (6.628° ≤ 2Θ ≤ 54.279°), 6017 unique (R_int_ = 0.0358, R_sigma_ = 0.0525), which were used in all calculations. The final R_1_ was 0.0453 (I > 2σ(I)) and wR_2_ was 0.12162 (all data).

Crystal Data for C_38_H_34_O_2_ **6a** (M = 522.65 g/mol): monoclinic, space group C2/c (no. 15), a = 27.338(2) Å, b = 6.6749(7) Å, c = 29.531(3) Å, β = 91.711(6)°, V = 5386.4(8) Å^3^, Z = 8, T = 100(2) K, μ(CuK_α_) = 0.601 mm^−1^, D_calc_ = 1.289 g/cm^3^, 15,880 reflections measured (6.468° ≤ 2Θ ≤ 130.032°), 4425 unique (R_int_ = 0. 1130, R_sigma_ = 0. 1406), which were used in all calculations. The final R_1_ was 0.0797 (I > 2σ(I)) and wR_2_ was 0.2122 (all data).

The X-ray crystal structures of compounds **6a** and **6b** were uploaded to the Cambridge Crystallographic Data Centre with the deposition numbers CCDC 2076991 for **6a** and CCDC 2076992 for **6b**. These data can be obtained free of charge from The Cambridge Crystallographic Data Centre via www.ccdc.cam.ac.uk/data_request/cif, accessed on 19 May 2021.

### 3.4. Computational Chemistry

The computations were performed using the M062X [50] functional along with the 6-311+G** basis set. Although the DA reaction is not very sensitive to solvent polarity, we took into account the effect of the toluene solvent in our computations. For this purpose, the polarisable continuum model using the integral equation formalism variant (IEFPCM) [51], as implemented in Gaussian 16 [52], was employed. Computations of harmonic vibrational frequencies confirm the nature of stationary points as minima or saddle points.

## 4. Conclusions

In summary, the computational investigation of the diastereoselectivity of the Diels–Alder reaction of strained bisdiene **1a** with strained bisdienophile **2** is in agreement with the experimental observations reported by Stoddart et al. [7,11,13,14,15]. The computations suggest that the geometrically more flexible bisdiene **1b** should undergo the DA reaction with bisdienophile **2** with lower diastereoselectivity, but the concave all-*syn* diastereomer **6a** required for construction of a molecular belt should be dominant. This is confirmed experimentally since **6a** is the dominant 2:1 cycloaddition product, while **6b** is a minor by-product. The concave **6a** can be chemically transformed into a molecular belt **12** by two subsequent DA reactions. The novel belt **12** could function as a synthetic intermediate for the synthesis of a photoprecursor for [11]cyclacene.

## Figures and Tables

**Table 1:** ^1^H NMR and ^13^C NMR (DEPT 135) data for compounds **11a** and **12**.

No.	11a		12	
	δ_C_	δ_H_	δ_C_	δ_H_
1	101.2, CH_2_	H_a_: 4.70, H_b_: 4.86	31.16^a^, CH_2_ or 31.19^a^, CH_2_	H_a_/H_b_: 2.57–2.48/2.20–2.04
2	144.4, C	-	147.5, C or 147.4, C	-
3	53.6, CH	3.88–3.86	57.0, CH	4.14–4.12
4	133.8, CH	6.39–6.37	139.8, CH	6.75–6.73
5	137.5, C	-	147.5, C or 147.4, C	-
6	30.7, CH_2_	H_a_/H_b_: 2.60–2.55/2.18–2.12	31.16^a^, CH_2_ or 31.19^a^, CH_2_	H_a_/H_b_: 2.57–2.48/2.20–2.04
7	43.1, CH	1.36–1.31 or 1.31–1.25 or 1.25–1.20	46.3, CH or 45.8, CH	1.36–1.31 or 1.31–1.25 or 1.25–1.20
8	85.19 ^a^, CH or 85.24 ^a^, CH	4.87	84.3, CH or 84.0, CH	4.79
9	144.8, C or 144.7, C	-	145.2, C or 145.1, C	-
10	110.2, CH	6.95	110.0, CH	6.85
11	144.8, C or 144.7, C	-	145.2, C or 145.1, C	-
12	85.19 ^a^, CH or 85.24 ^a^, CH	4.85	84.3, CH or 84.0, CH	4.82
13	42.9, CH	1.77–1.71	47.2, CH	1.36–1.31 or 1.31–1.25 or 1.25–1.20
14	31.4, CH_2_	H_a_/H_b_: 2.70–2.65/2.12–2.06	31.7, CH_2_	H_a_/H_b_: 2.57–2.48/2.23–2.17
15	143.8, C	-	147.8, C	-
16	55.3, CH	4.15–4.13	56.3, CH	4.23–4.21
17	139.5, CH	6.77–6.75	139.0, CH	6.82–6.80
18	140.8, C	-	143.3, C	-
19	33.7, CH_2_	H_a_, H_b_: 3.49–3.33	34.3, CH_2_	H_a_/H_b_: 3.53–3.46/3.26–3.19
20	130.7, C	-	133.5, C	-
21	120.9, CH	6.93	118.8, CH	6.77
22	146.6, C	-	143.7, C	-
23	82.2, CH	5.61	84.1, CH	4.81
24	143.0, CH	6.91	46.3, CH or 45.8, CH	1.36–1.31 or 1.31–1.25 or 1.25–1.20

^a^ chemical shifts are rounded to two decimals to distinguish them.

## Supplementary Materials

Charts of NMR spectra (^1^H, ^13^C, selected COSY and NOESY), mass spectra, chromatograms, computational method evaluation, relative energies, figures of transition states discussed in the text, Cartesian coordinates of stationary points are available online.

## Sample Availability

Not available.
