# Supplementary material for: Synthesis of the [11]Cyclacene Framework by Repetitive Diels–Alder Cycloadditions
Source: Molecules. 2021 May 20;26(10):3047. doi: 10.3390/molecules26103047 (PMC8161356; doi:10.3390/molecules26103047)
Supplement: Supplementary file 1 [file molecules-26-03047-s001.zip › molecules-1203245-SI.pdf]

Supporting Information

Synthesis of the [11]Cyclacene Framework by Repetitive Diels-Alder Cycloadditions

John B. Bauer,[a] Fatima Diab,[a] Cäcilia Maichle-Mössmer,[b] Hartmut Schubert,[b] Holger F. Bettinger*[a]

[a]Institut für Organische Chemie, Universität Tübingen, Auf der Morgenstelle 18, 72076 Tübingen, Germany

[b]Institut für Anorganische Chemie, Universität Tübingen, Auf der Morgenstelle 18, 72076 Tübingen, Germany

* Correspondence: holger.bettinger@uni-tuebingen.de

Table of Contents

1. NMR Spectra 3

2. ESI/APCI High Resolution Mass Spectra 37

3. HPLC-MS... 43

4. Computational Details 45

5. References 75

1. NMR Spectra

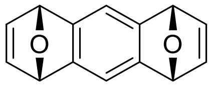

2

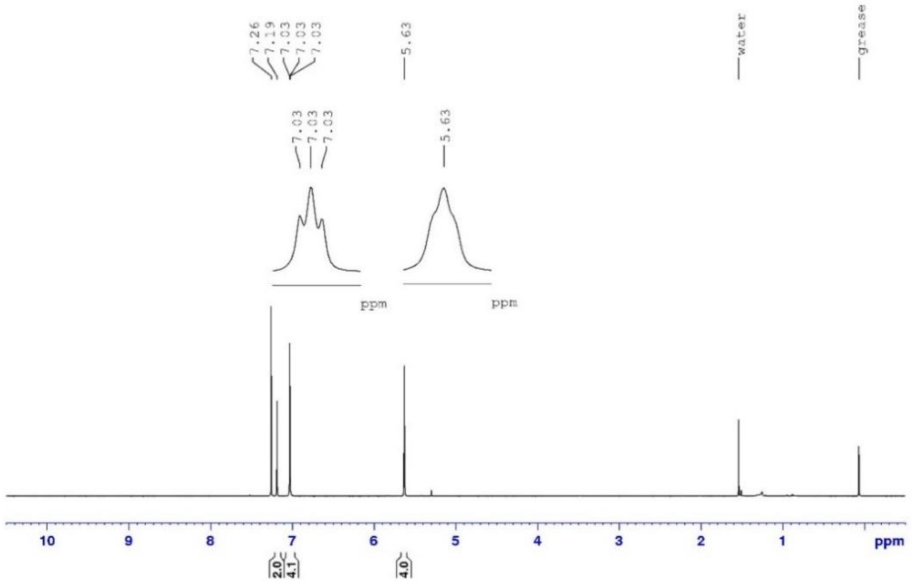

Figure S1.^{1}H NMR of **2** in $CDCl_3$ at a 400 MHz spectrometer at 298 K (10.5 ppm- -0.5 ppm).

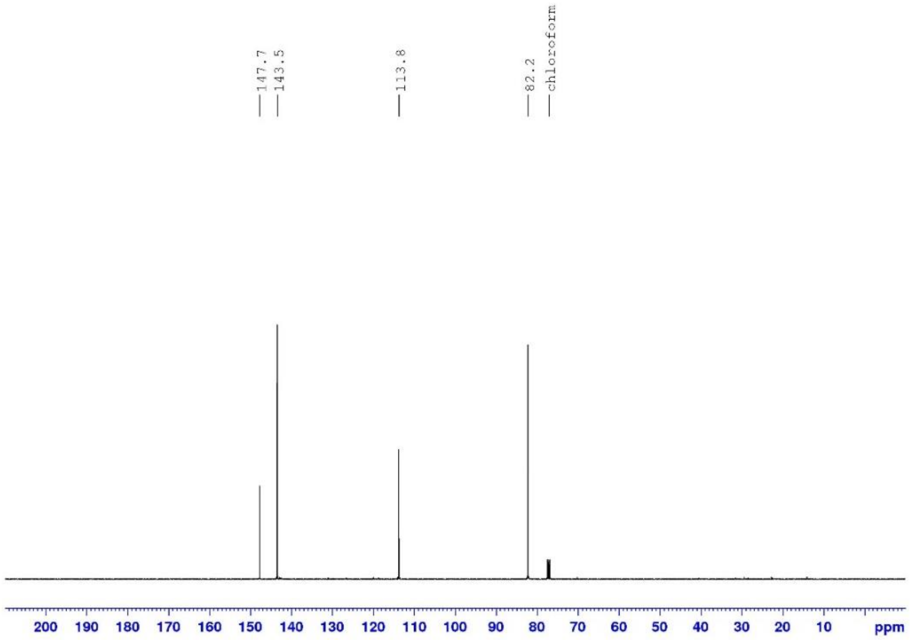

Figure S2.^{13}C{^{1}H} NMR of **2** in $CDCl_3$ at a 100 MHz spectrometer at 298 K (210 ppm- -10 ppm).

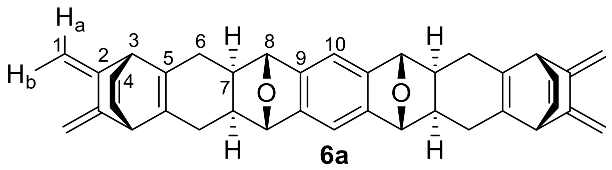

Table S1. ^{1}H NMR and 13C NMR data for compound **6a**.

No.	δ_C	δ_H
1	101.2	H_a: 4.70, H_b: 4.86
2	144.4	-
3	53.7	3.92-3.88
4	133.8	6.41-6.37
5	137.7	-
6	30.8	H_a /H_b: 2.64-2.56/ 2.22-2.14
7	43.5	1.90-1.83
8	85.2	4.89
9	144.8	-
10	110.3	6.98

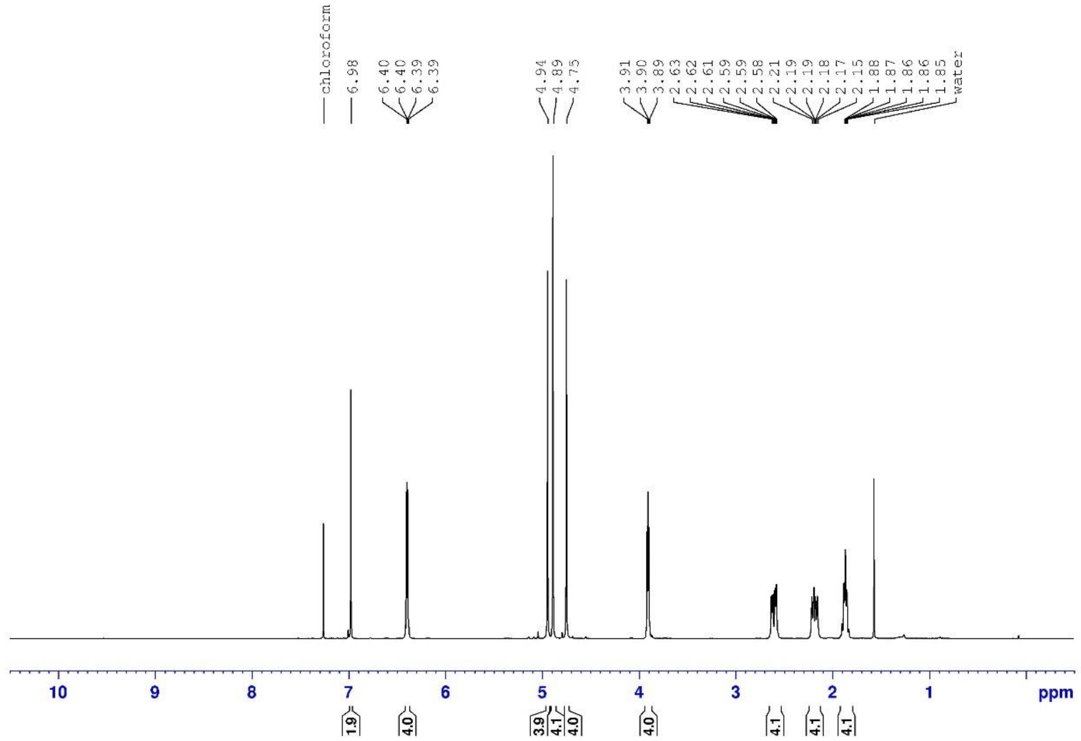

Figure S3. ^{1}H NMR of **6a** in $CDCl_3$ at a 400 MHz spectrometer at 298 K (10.5 ppm- -0.5 ppm).

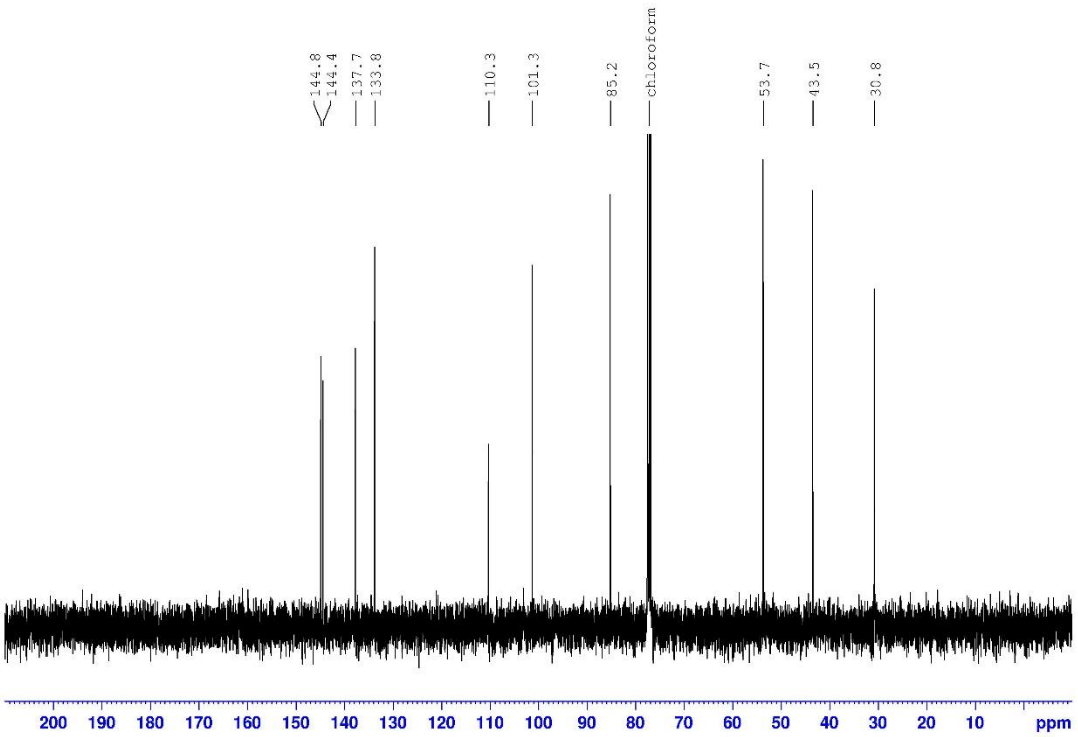

Figure S4. $^{13}C\{^{1}H\}$ NMR of **6a** in $CDCl_3$ at a 100 MHz spectrometer at 298 K (210 ppm- -10 ppm).

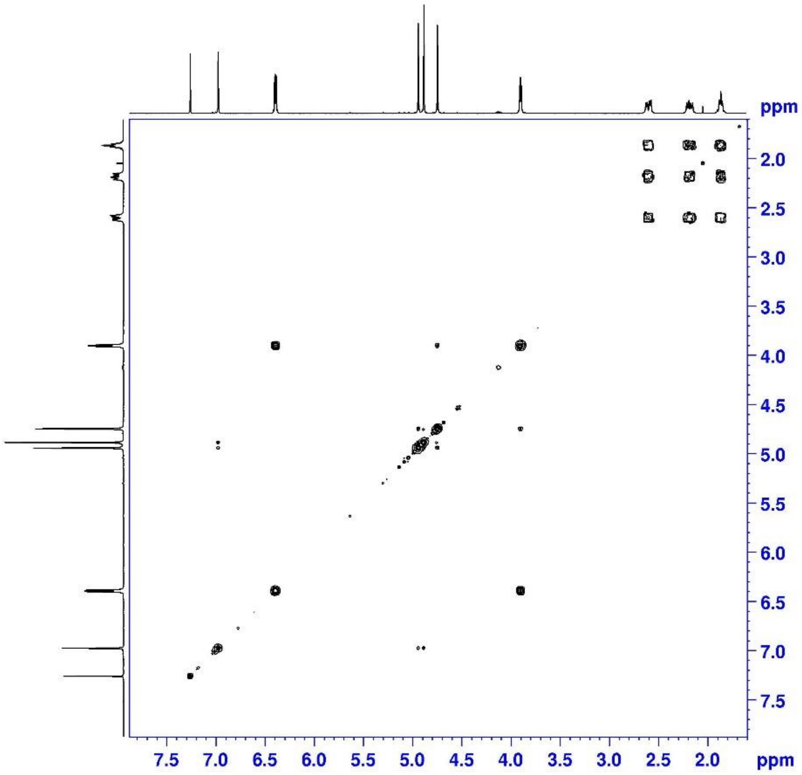

Figure S5. $^{1}H,^{1}H$ COSY NMR spectrum of **6a** in $CDCl_3$ at a 400 MHz spectrometer at 298 K.

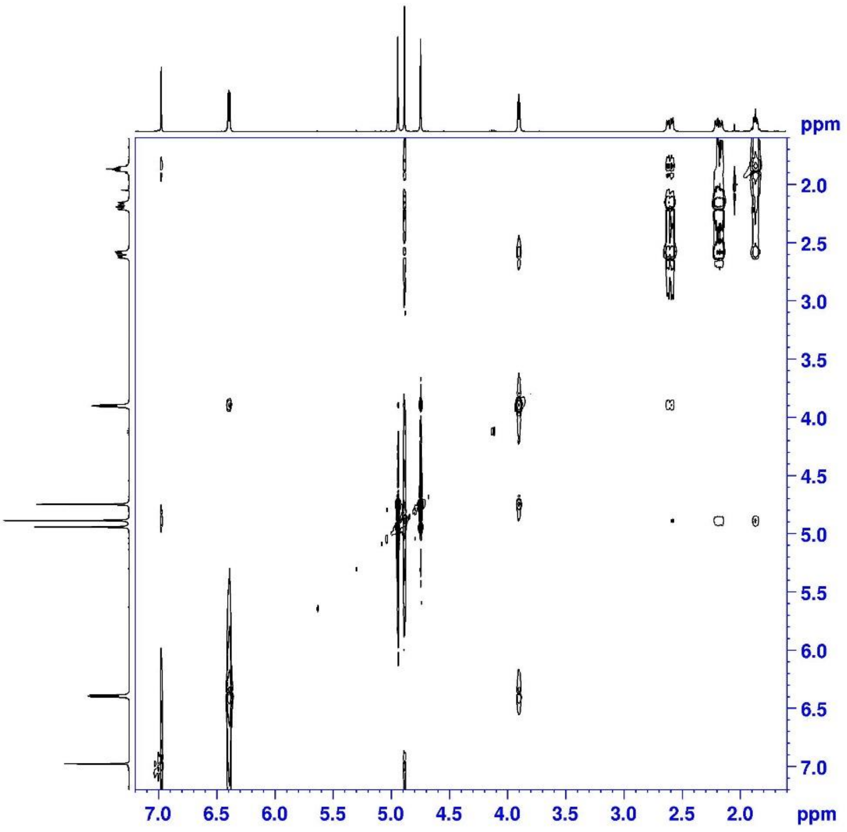

Figure S6. ^{1}H,^{1}H NOESY NMR spectrum of **6a** in $CDCl_3$ at a 400 MHz spectrometer at 298 K.

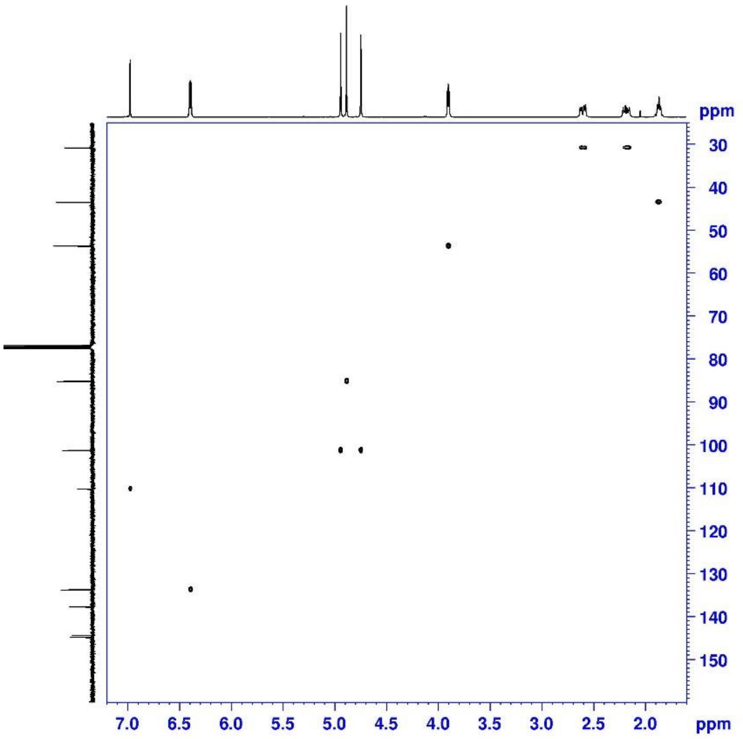

Figure S7. ^{1}H,^{13}C HSQC NMR spectrum of **6a** in $CDCl_3$ at a 400 MHz spectrometer at 298 K.

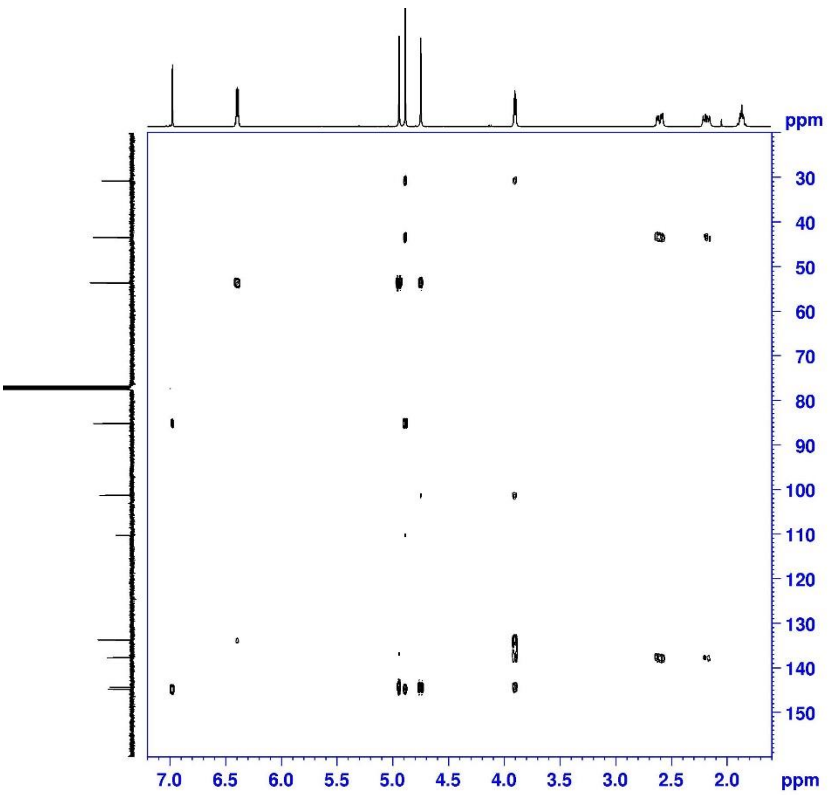

Figure S8. $^{1}H,^{13}C$ HMBC NMR spectrum of **6a** in $CDCl_3$ at a 400 MHz spectrometer at 298 K.

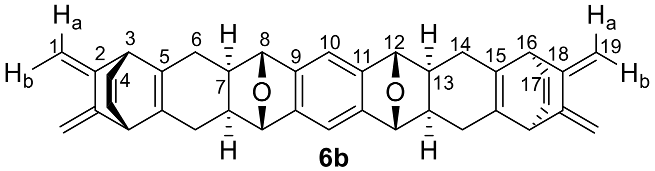

6b

Table S2. ^{1}H NMR and 13C NMR data for compound **6b**.

No.	δ_C	δ_H
1	101.6 or 101.3	H_a: 4.80 or 4.75, H_b: 5.04 or 4.94
2	144.4 or 144.3	-
3	53.6 or 53.5	3.92-3.85
4	133.9 or 133.8	6.41-6.36
5	137.7 or 136.8	-
6	30.8 or 30.1	H_a /H_b: 2.65-2.55/ 2.24-2.11
7	43.3 or 41.4	1.94-1.86
8	85.2 or 85.2	4.90 or 4.89
9	144.8 or 144.6	-
10	110.3	7.00
11	144.8 or 144.6	-
12	85.2 or 85.2	4.90 or 4.89
13	43.3 or 41.4	1.94-1.86
14	30.8 or 30.1	H_a /H_b: 2.65-2.55/ 2.24-2.11
15	137.7 or 136.8	-
16	53.6 or 53.5	6.41-6.36
17	133.9 or 133.8	3.92-3.85
18	144.4 or 144.3	-
19	101.6 or 101.3	H_a: 4.80 or 4.75, H_b: 5.04 or 4.94

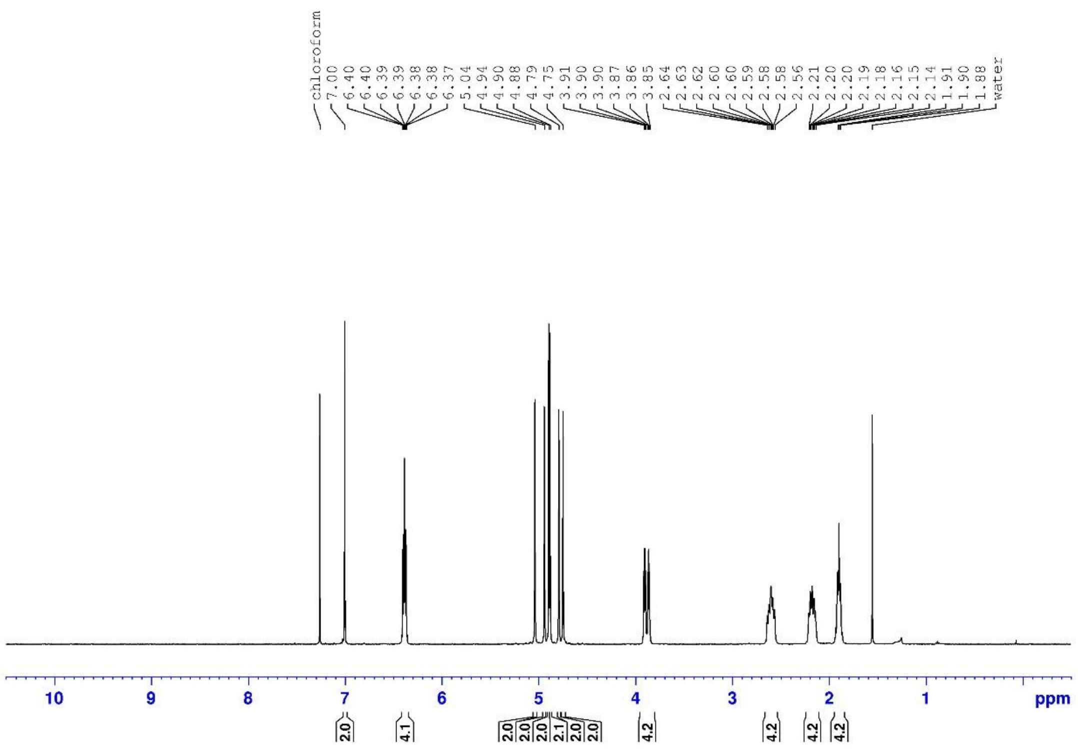

Figure S9. ^{1}H NMR of **6b** in $CDCl_3$ at a 400 MHz spectrometer at 298 K (10.5 ppm- -0.5 ppm).

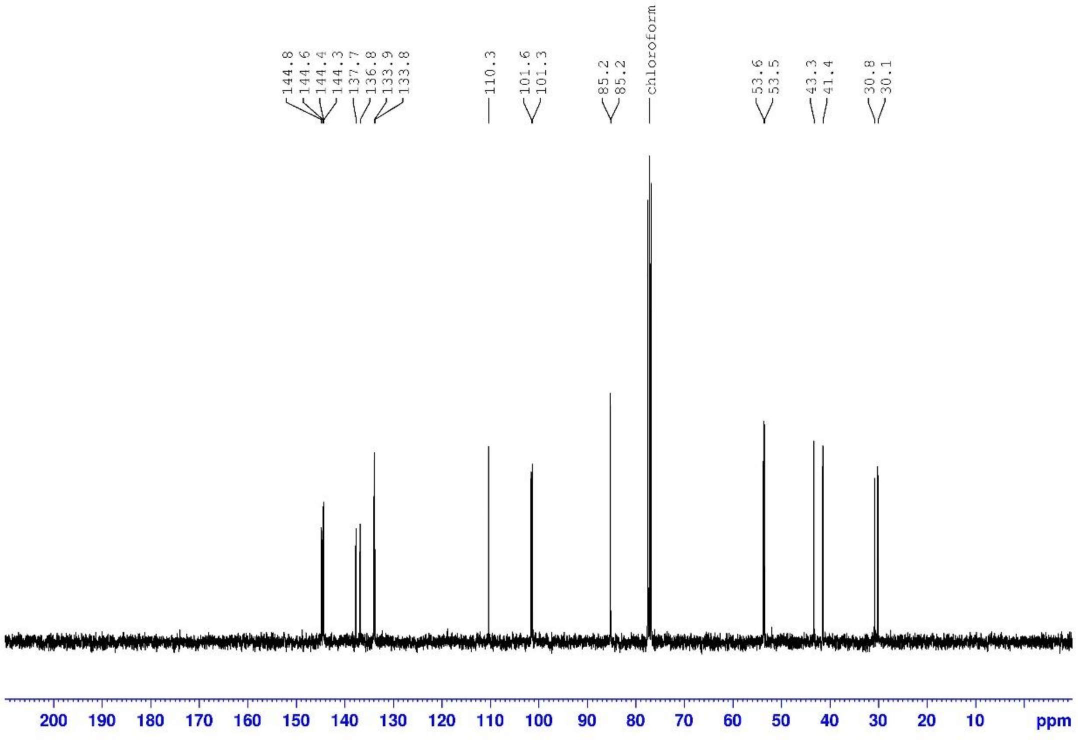

Figure S10. ^{13}C{^{1}H} NMR of **6b** in $CDCl_3$ at a 100 MHz spectrometer at 298 K (210 ppm- -10 ppm).

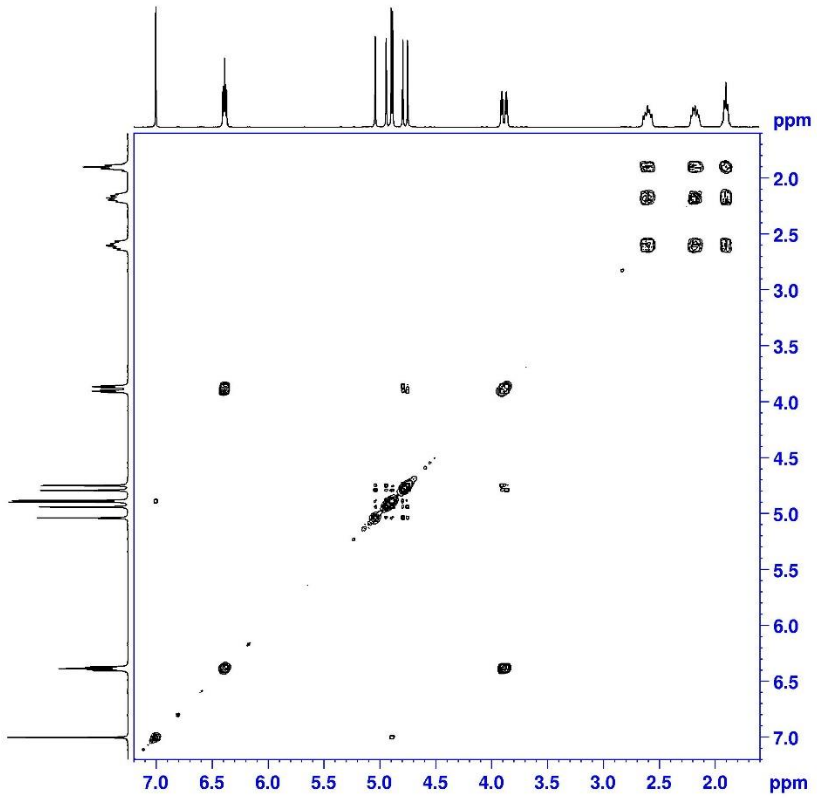

Figure S11. ^{1}H,^{1}H COSY NMR spectrum of **6b** in $CDCl_3$ at a 400 MHz spectrometer at 298 K.

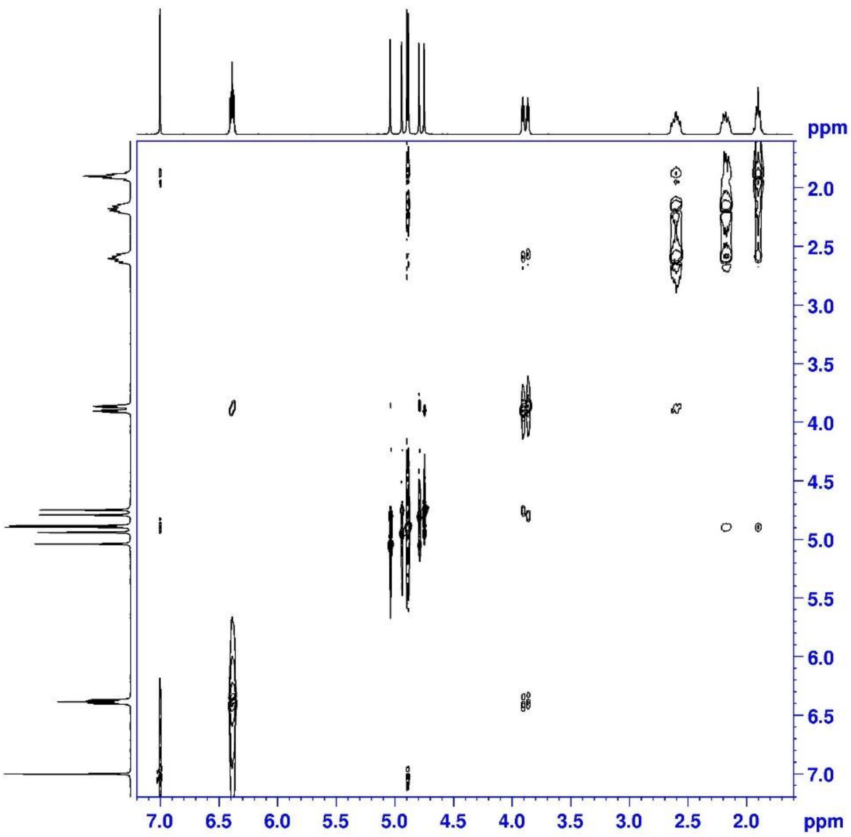

Figure S12. ^{1}H,^{1}H NOESY NMR spectrum of **6b** in $CDCl_3$ at a 400 MHz spectrometer at 298 K.

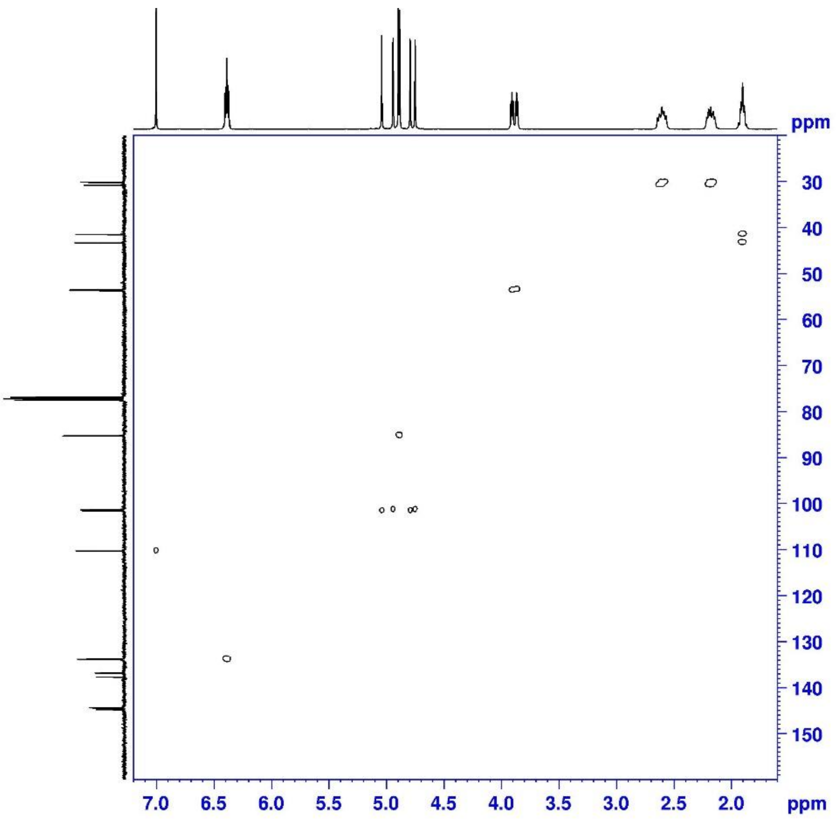

Figure S13. $^{1}H,^{13}C$ HSQC NMR spectrum of **6b** in $CDCl_3$ at a 400 MHz spectrometer at 298 K.

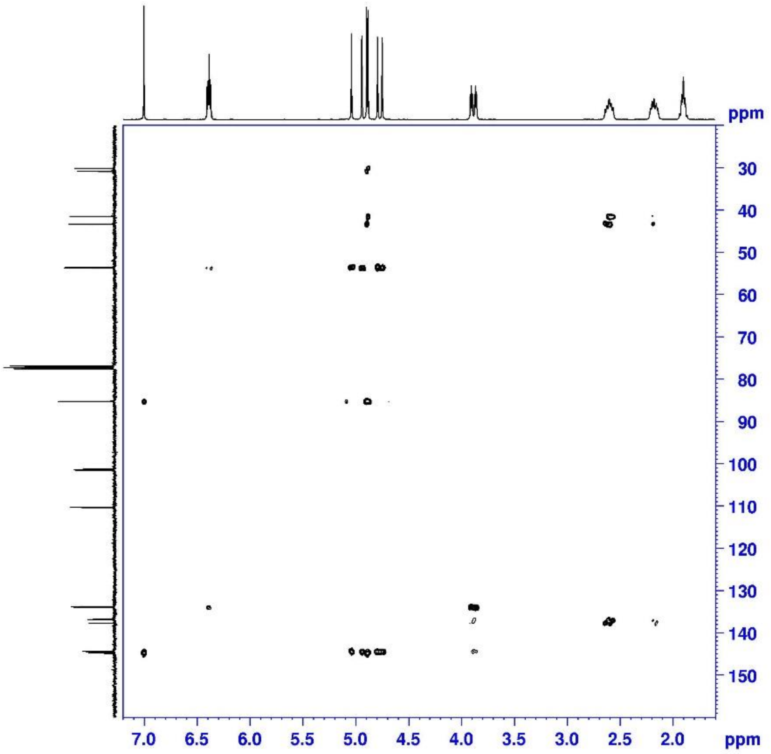

Figure S14. $^{1}H,^{13}C$ HMBC NMR spectrum of **6b** in $CDCl_3$ at a 400 MHz spectrometer at 298 K.

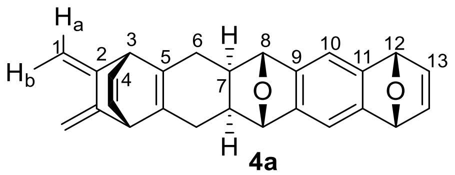

4a

Table S3. ^{1}H NMR and 13C NMR data for compound **4a**.

No.	δ_C	δ_H
1	101.3	H_a: 4.75, H_b: 4.94
2	144.4	-
3	53.6	3.93-3.89
4	133.7	6.42-6.38
5	137.7	-
6	30.7	H_a /H_b: 2.65-2.56/ 2.23-2.14
7	43.4	1.94-1.87
8	85.0	4.88
9	143.4	-
10	112.1	7.07
11	148.7	-
12	82.5	5.63
13	143.5	7.01-6.99

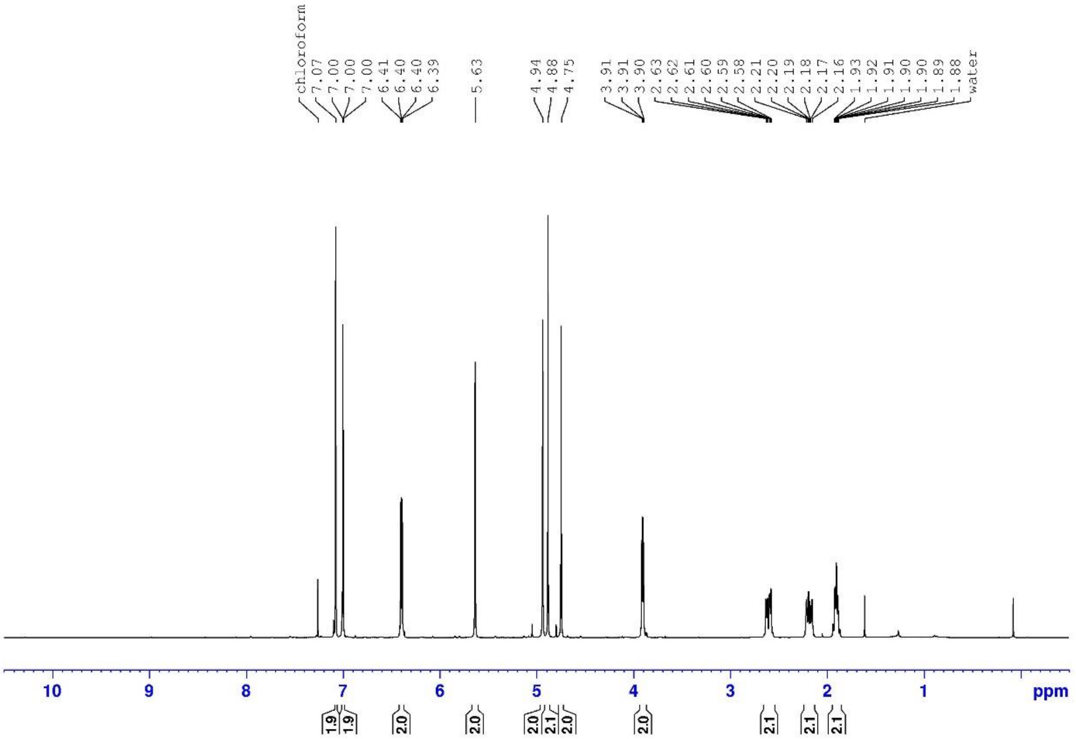

Figure S15. ^{1}H NMR of **4a** in $CDCl_3$ at a 400 MHz spectrometer at 298 K (10.5 ppm- -0.5 ppm).

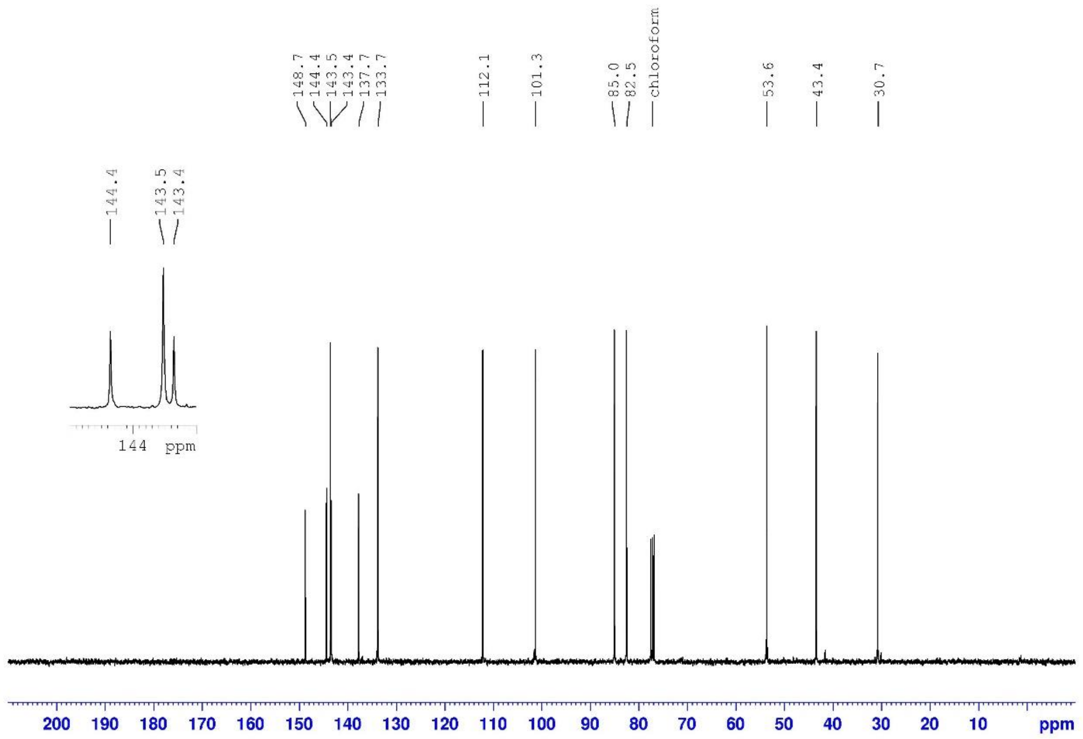

Figure S16. $^{13}C\{^{1}H\}$ NMR of **4a** in $CDCl_3$ at a 100 MHz spectrometer at 298 K (210 ppm- -10 ppm).

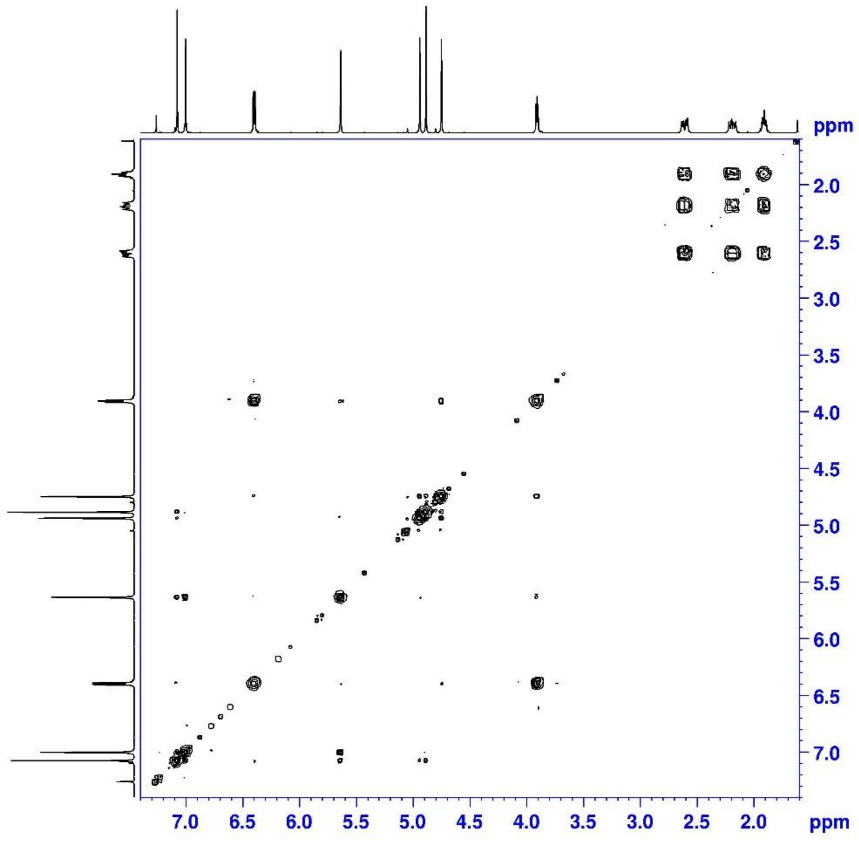

Figure S17. $^{1}H,^{1}H$ COSY NMR spectrum of **4a** in $CDCl_3$ at a 400 MHz spectrometer at 298 K.

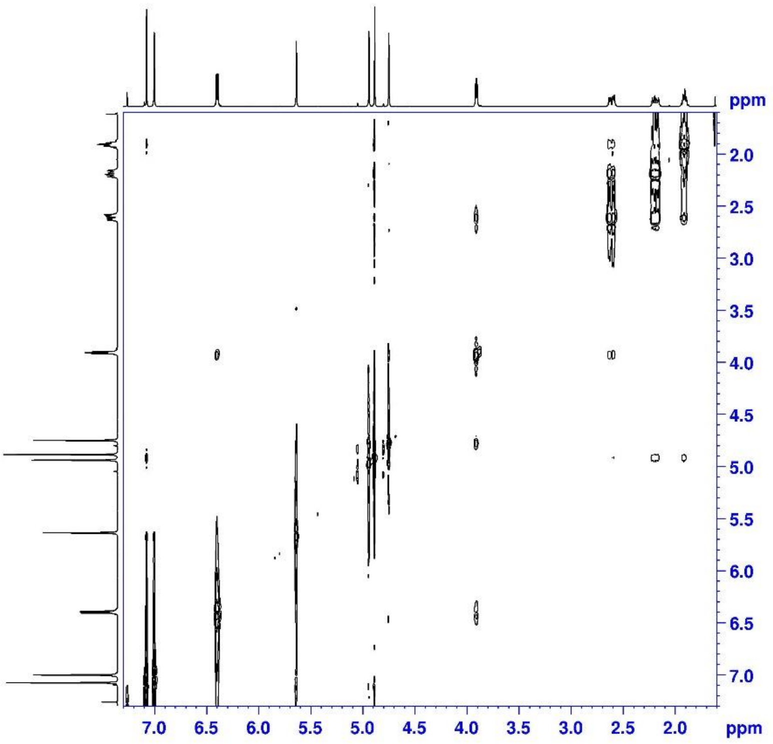

Figure S18. $^{1}H,^{1}H$ NOESY NMR spectrum of **4a** in $CDCl_3$ at a 400 MHz spectrometer at 298 K.

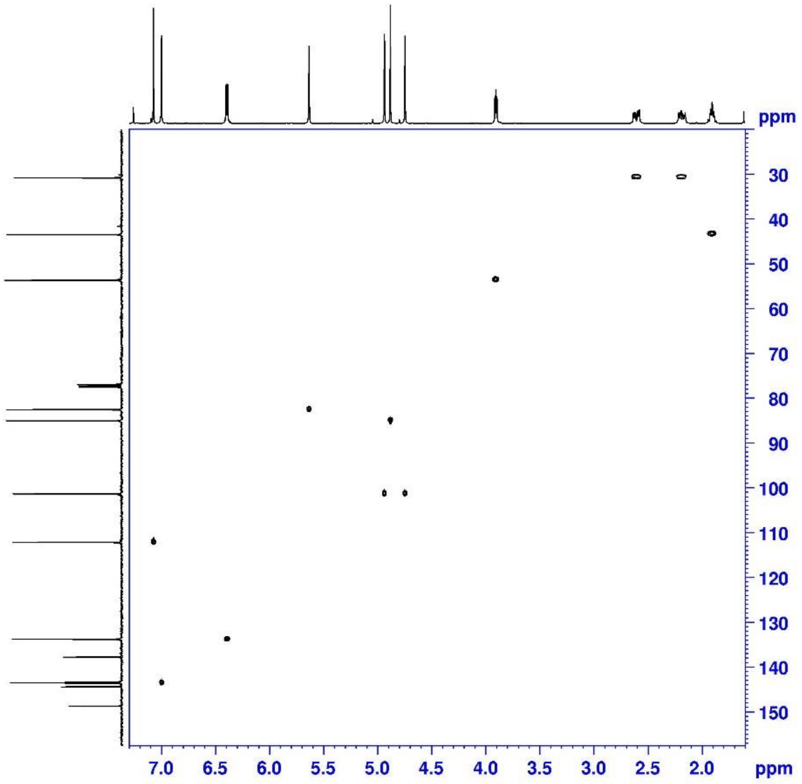

Figure S19. $^{1}H,^{13}C$ HSQC NMR spectrum of **4a** in $CDCl_3$ at a 400 MHz spectrometer at 298 K.

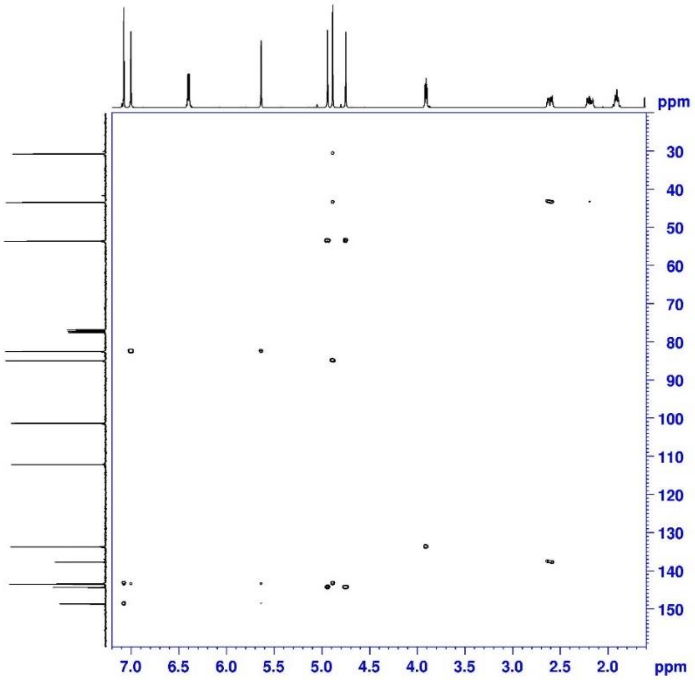

Figure S20. $^{1}H,^{13}C$ HMBC NMR spectrum of **4a** in $CDCl_3$ at a 400 MHz spectrometer at 298 K.

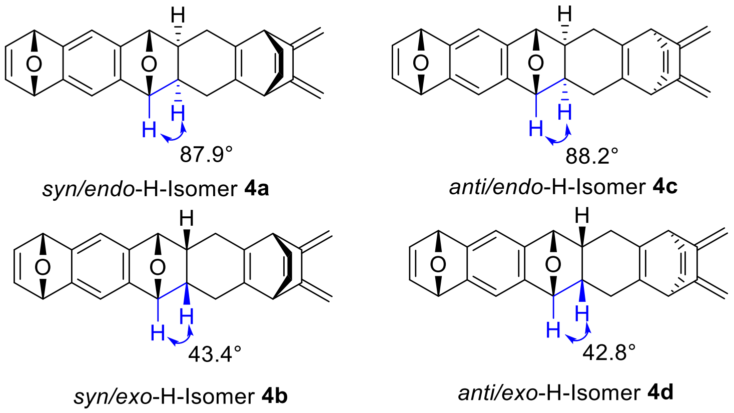

Figure S21. Computed (M062X/6-311+G**/toluene) dihedral angles between protons highlighted in blue for the four diastereomers **4a-d.**

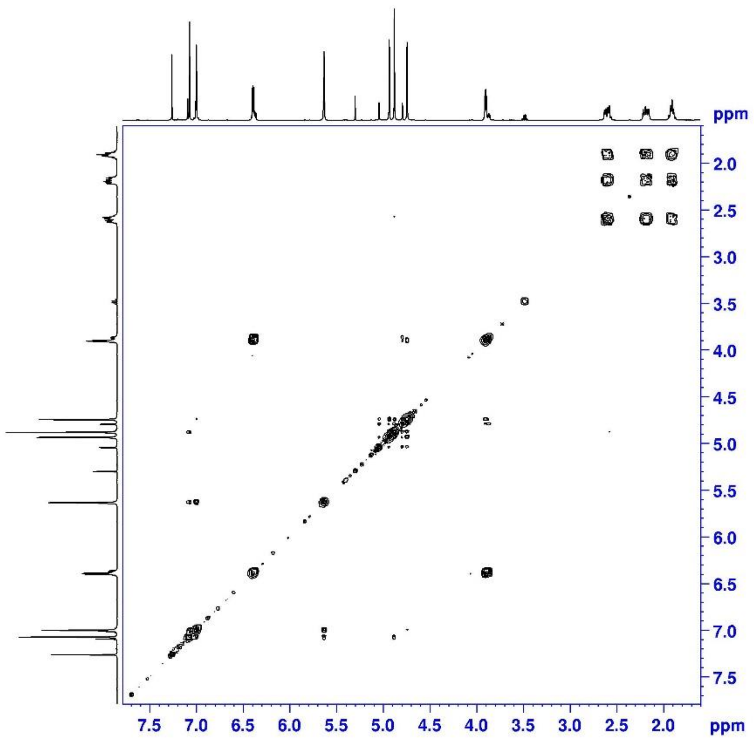

Figure S22. ^{1}H,^{1}H COSY NMR spectra of the mixture of **4a** and **4c** at a 400 MHz spectrometer at 298 K. A cross peak would be expected for exo-H subunits.

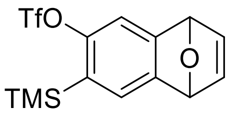

10

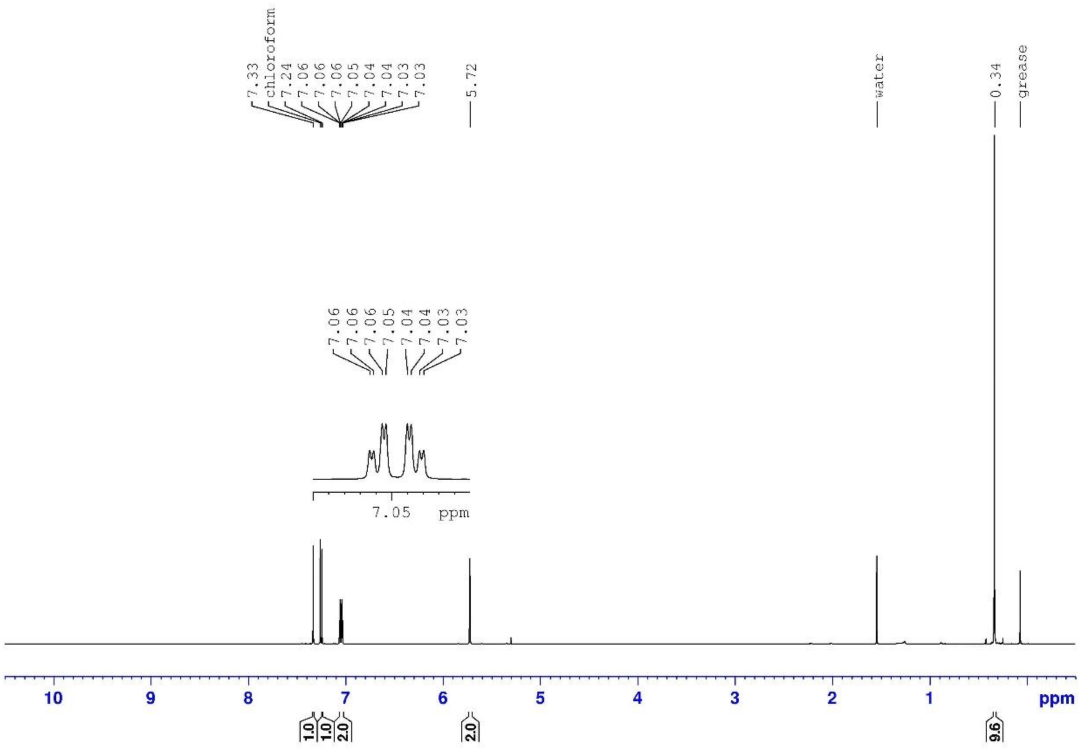

Figure S23. ^{1}H NMR of **10** in $CDCl_3$ at a 700 MHz spectrometer at 298 K (10.5 ppm- -0.5 ppm).

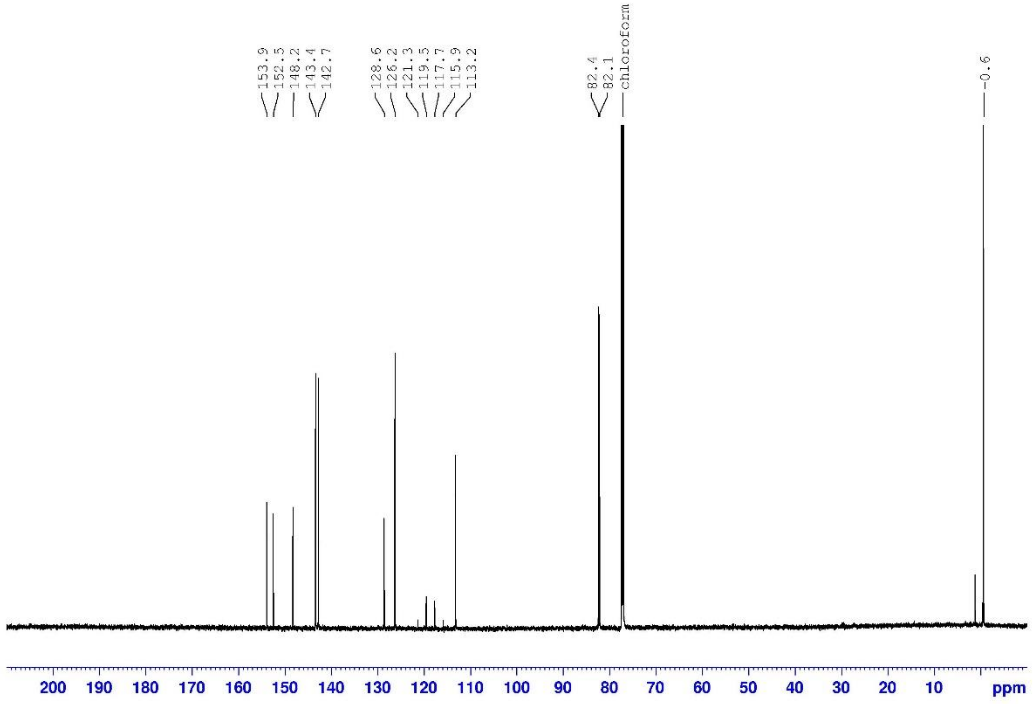

Figure S24. $^{13}C\{^{1}H\}$ NMR of **10** in $CDCl_3$ at a 176 MHz spectrometer at 298 K (210 ppm- -10 ppm).

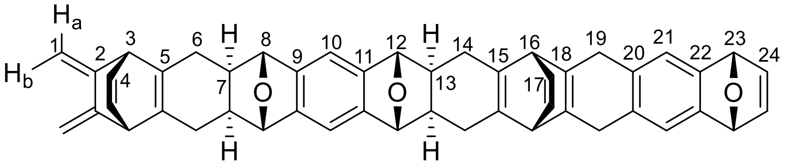

11a

Table S4. ^{1}H NMR and 13C NMR (DEPT 135) data for compound **11a**.

No.	δ_C	δ_H
1	101.2, CH_2	H_a: 4.70, H_b: 4.86
2	144.4, C	-
3	53.6, CH	3.88-3.86
4	133.8, CH	6.39-6.37
5	137.5, C	-
6	30.7, CH_2	H_a/ H_b: 2.60-2.55/ 2.18-2.12
7	43.1, CH	1.36-1.31 or 1.31-1.25 or 1.25-1.20
8	85.19[a], CH or 85.24[a], CH	4.87
9	144.8, C or 144.7, C	-
10	110.2, CH	6.95
11	144.8, C or 144.7, C	-
12	85.19[a], CH or 85.24[a], CH	4.85
13	42.9, CH	1.77-1.71
14	31.4, CH_2	H_a/ H_b: 2.70-2.65/ 2.12-2.06
15	143.8, C	-
16	55.3, CH	4.15-4.13
17	139.5, CH	6.77-6.75
18	140.8, C	-
19	33.7, CH_2	H_a, H_b: 3.49-3.33
20	130.7, C	-
21	120.9, CH	6.93
22	146.6, C	-
23	82.2, CH	5.61
24	143.0, CH	6.91

[a]chemical shifts are rounded to two decimals to distinguish them.

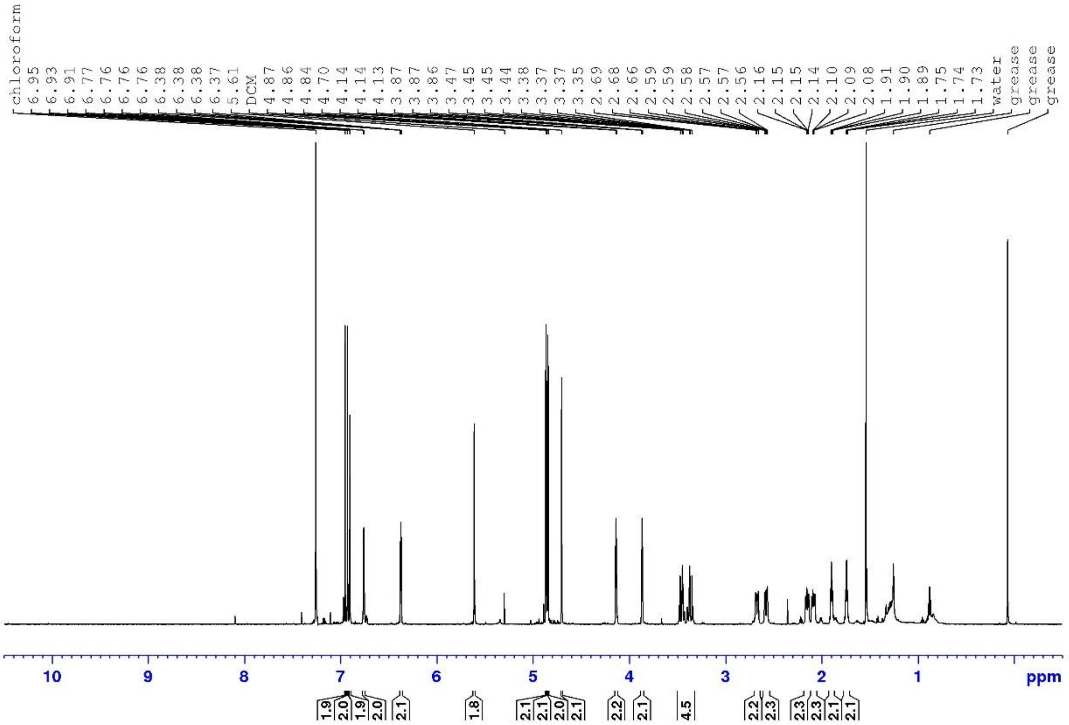

Figure S25. ^{1}H NMR of **11a** in $CDCl_3$ at a 700 MHz spectrometer at 298 K, overview. (10.5 ppm- -0.5 ppm).

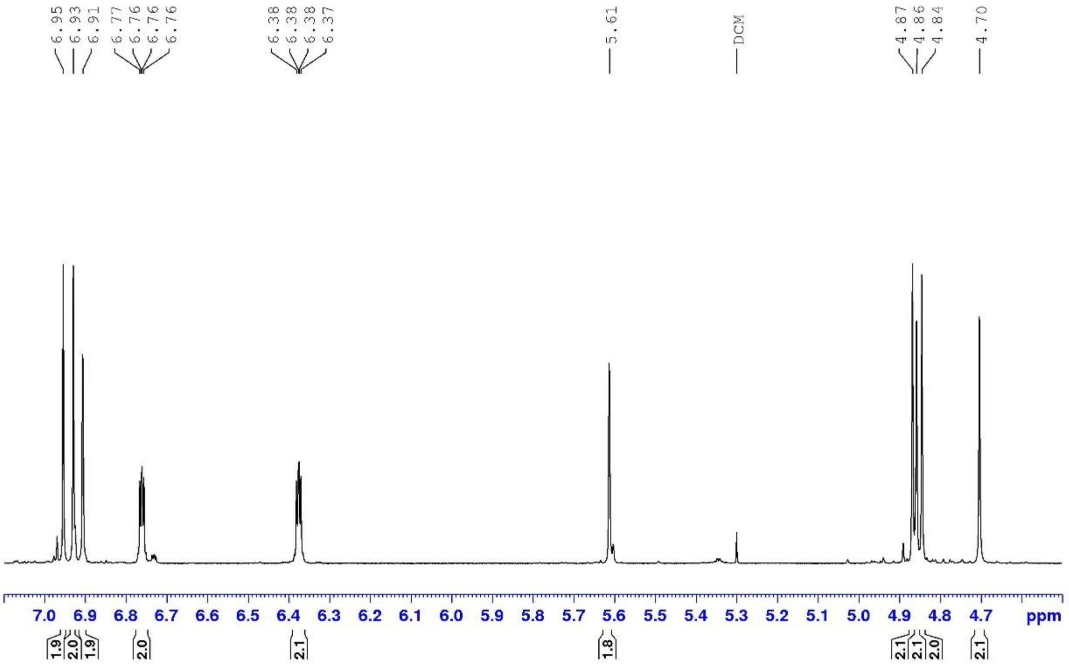

Figure S26. ^{1}H NMR of **11a** in $CDCl_3$ at a 700 MHz spectrometer at 298 K, part 1 (7.1 ppm-4.5 ppm).

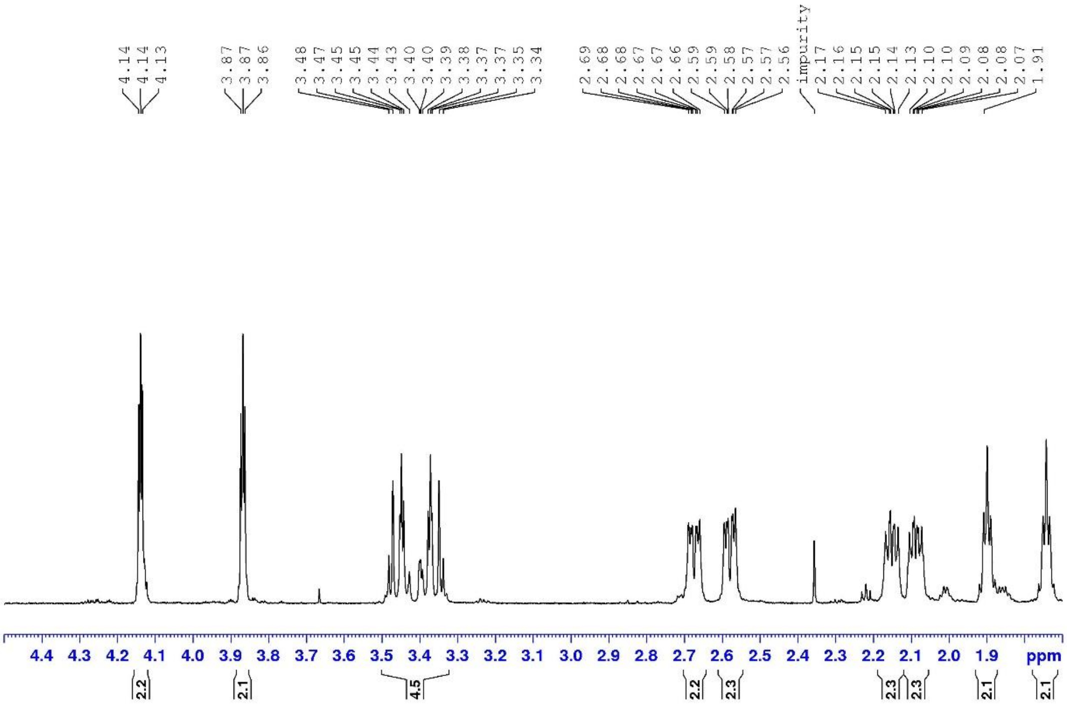

Figure S26. ^{1}H NMR of **11a** in $CDCl_3$ at a 700 MHz spectrometer at 298 K, part 2 (4.5ppm-1.7 ppm).

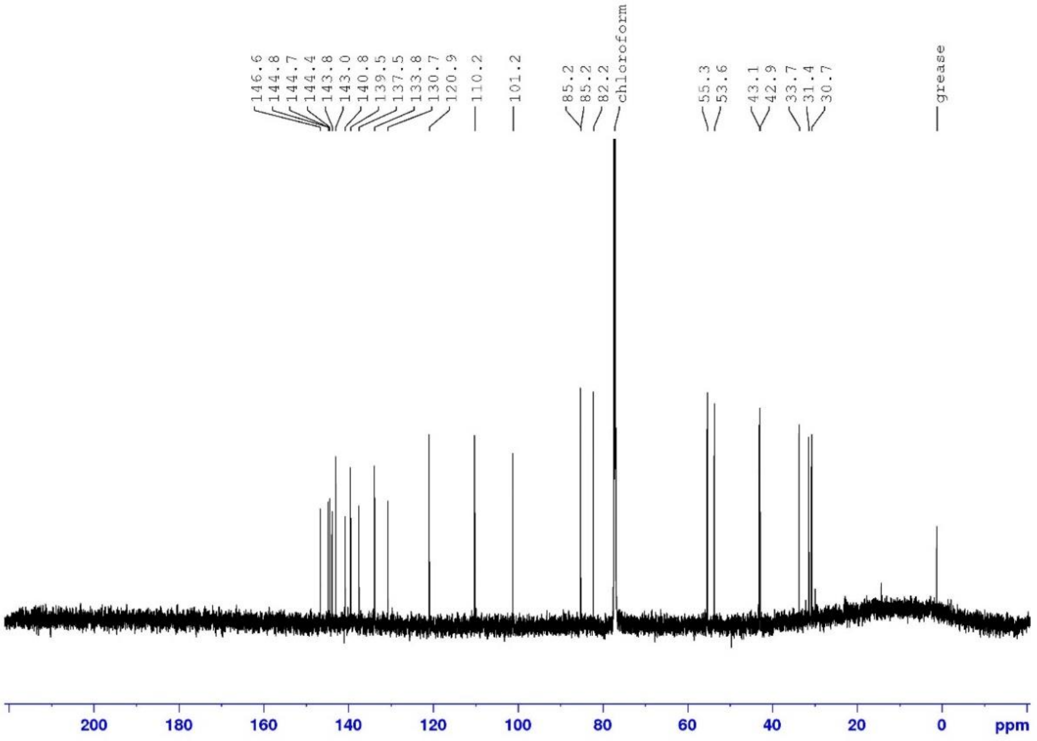

Figure S27. ^{13}C{^{1}H} NMR of **11a** in $CDCl_3$ at a 176 MHz spectrometer at 298 K, overview (210 ppm--10 ppm).

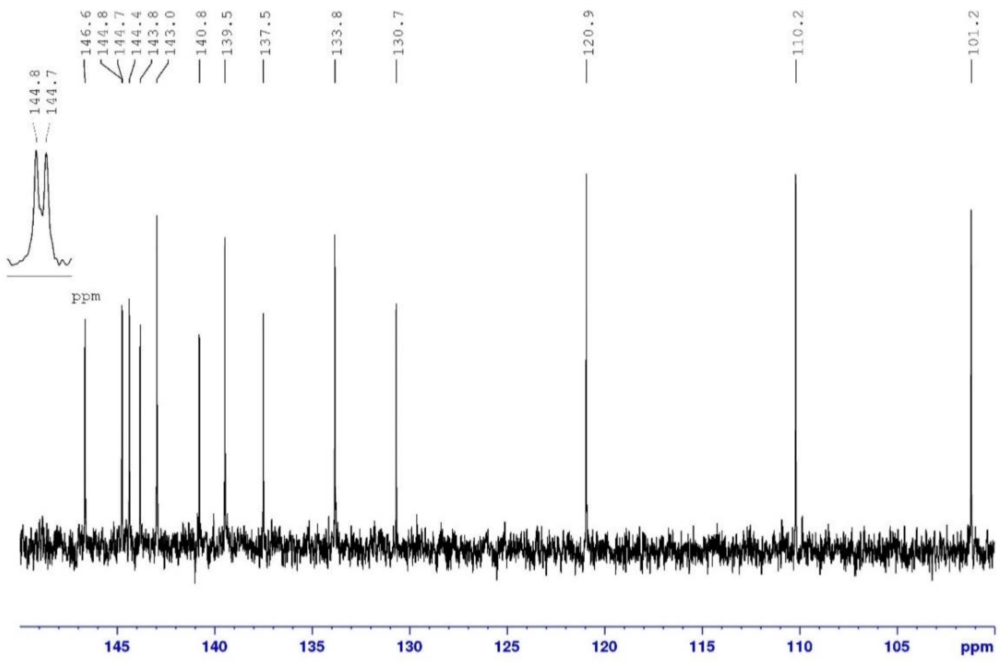

Figure S28. $^{13}C\{^{1}H\}$ NMR of **11a** in $CDCl_3$ at a 176 MHz spectrometer at 298 K, part 1 (150 ppm-100 ppm).

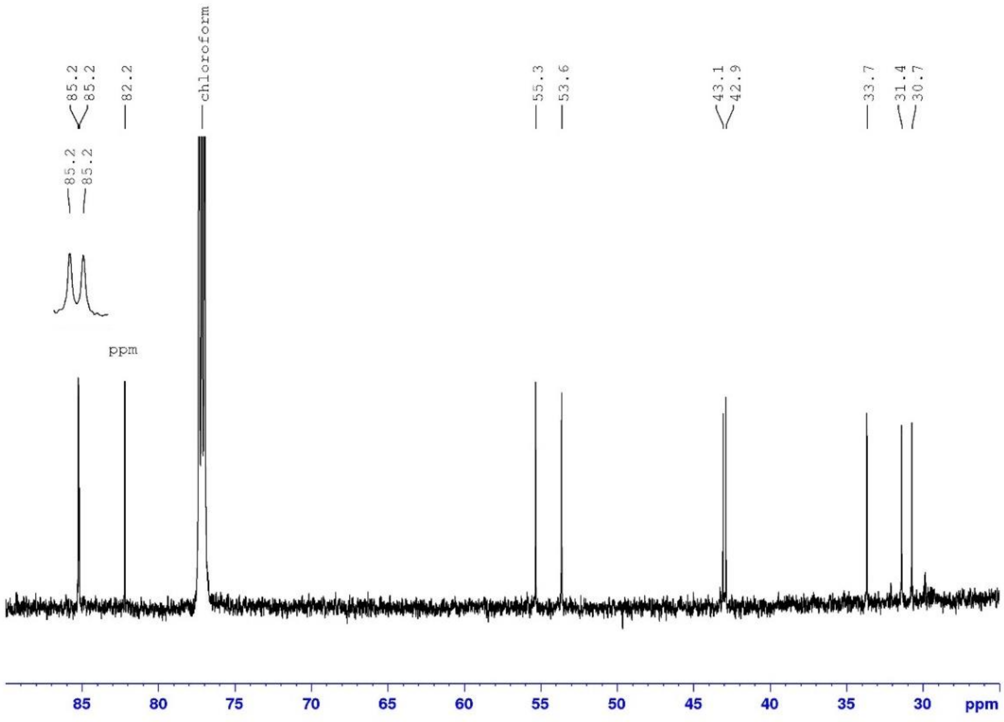

Figure S29. $^{13}C\{^{1}H\}$ NMR of **11a** in $CDCl_3$ at a 176 MHz spectrometer at 298 K, part 2 (90 ppm- 25 ppm).

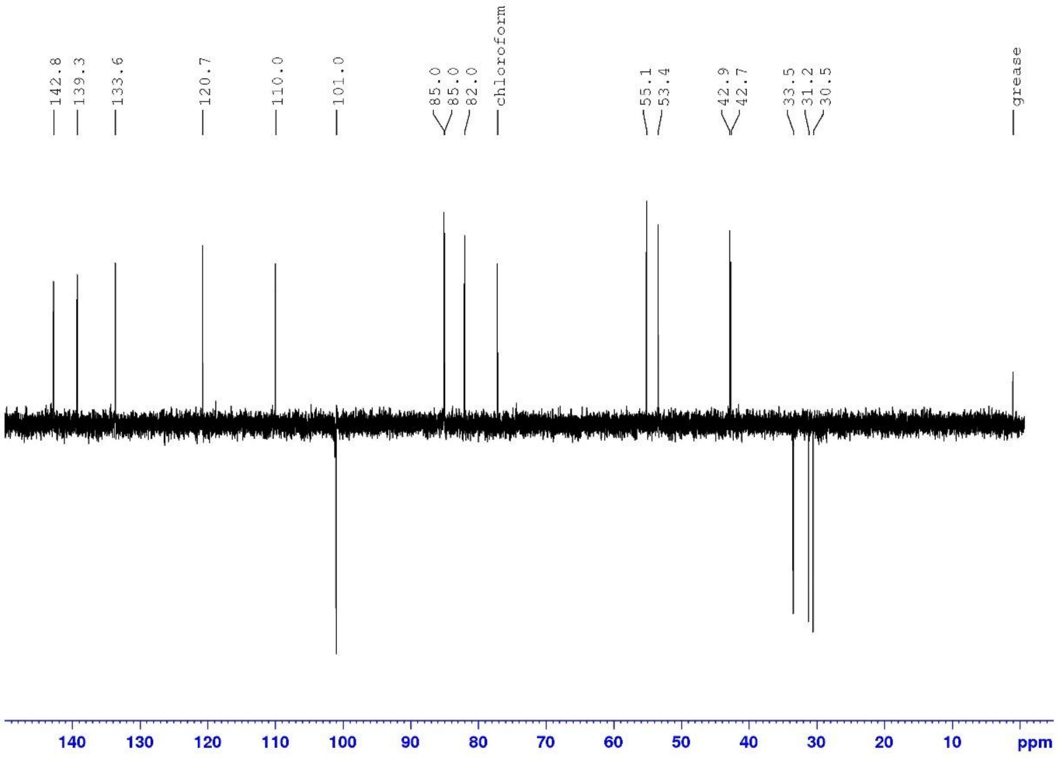

Figure S30. DEPT 135 NMR of **11a** in $CDCl_3$ at a 176 MHz spectrometer at 298 K (150 ppm- -5 ppm).

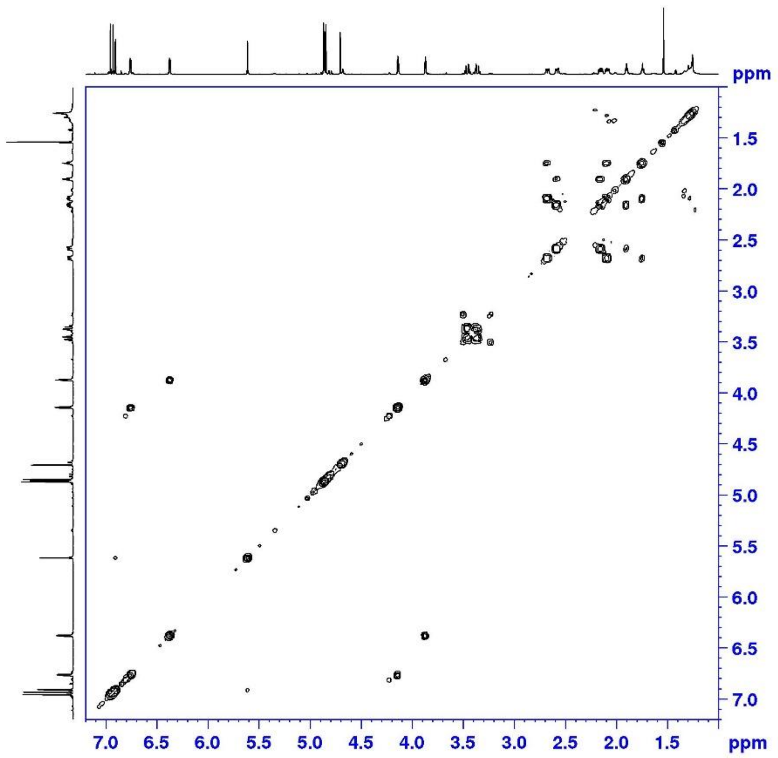

Figure S31. $^{1}H,^{1}H$ COSY NMR spectrum of **11a** in $CDCl_3$ at a 700 MHz spectrometer at 298 K.

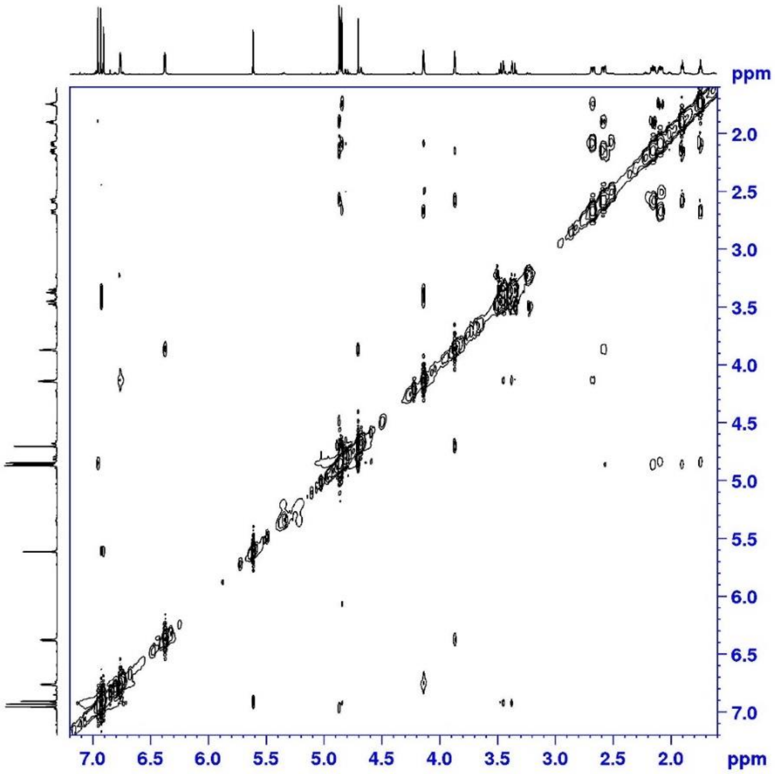

Figure S32. ^{1}H,^{1}H NOESY NMR spectrum of **11a** in $CDCl_3$ at a 700 MHz spectrometer at 298 K.

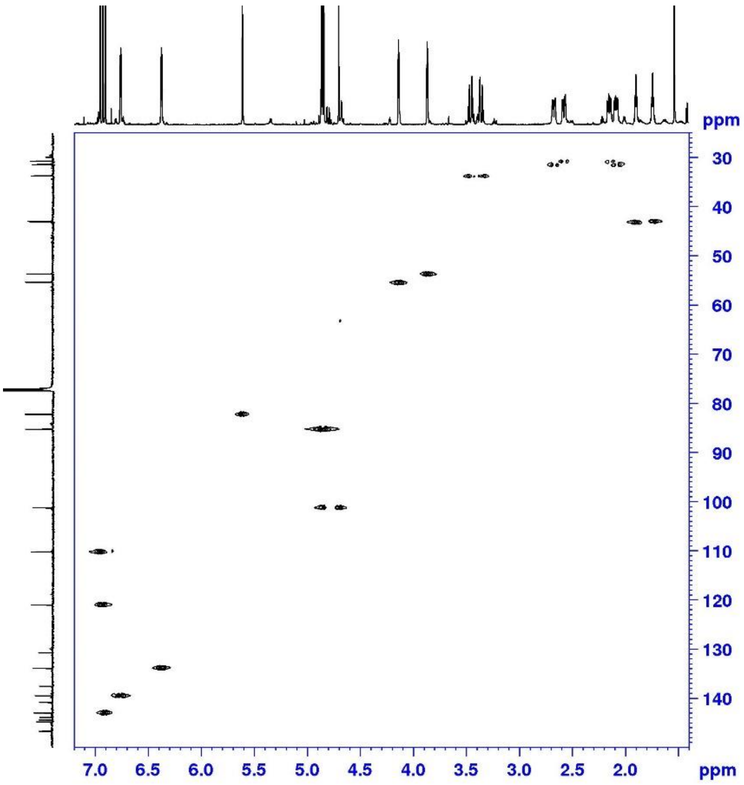

Figure S33. ^{1}H,^{13}C HSQC NMR spectrum of **11a** in $CDCl_3$ at a 700 MHz spectrometer at 298 K.

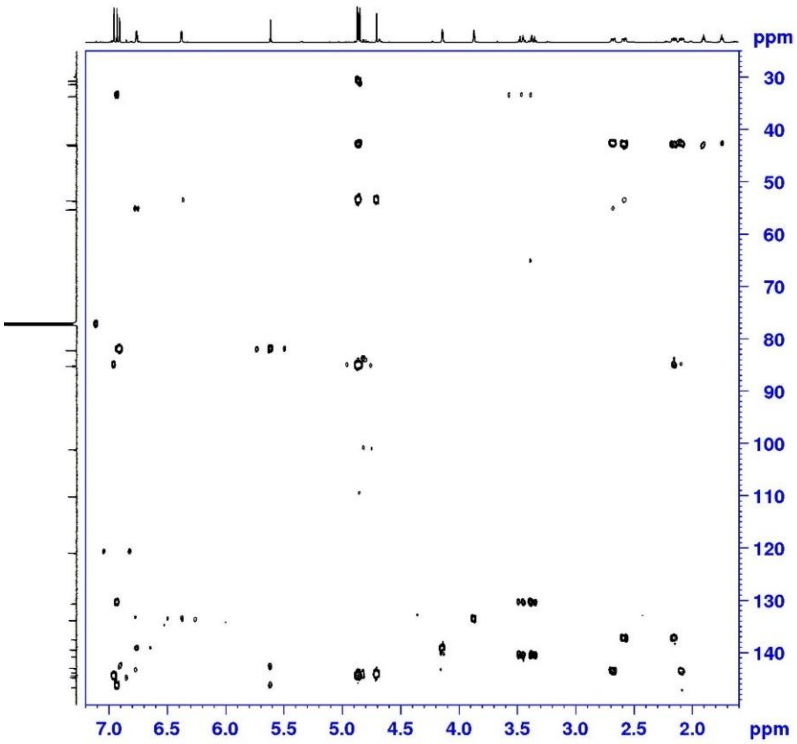

Figure S34. $^{1}H,^{13}C$ HMBC NMR spectrum of **11a** in $CDCl_3$ at a 700 MHz spectrometer at 298 K.

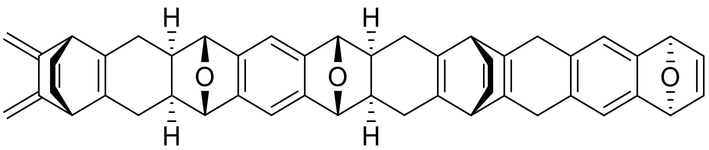

11b

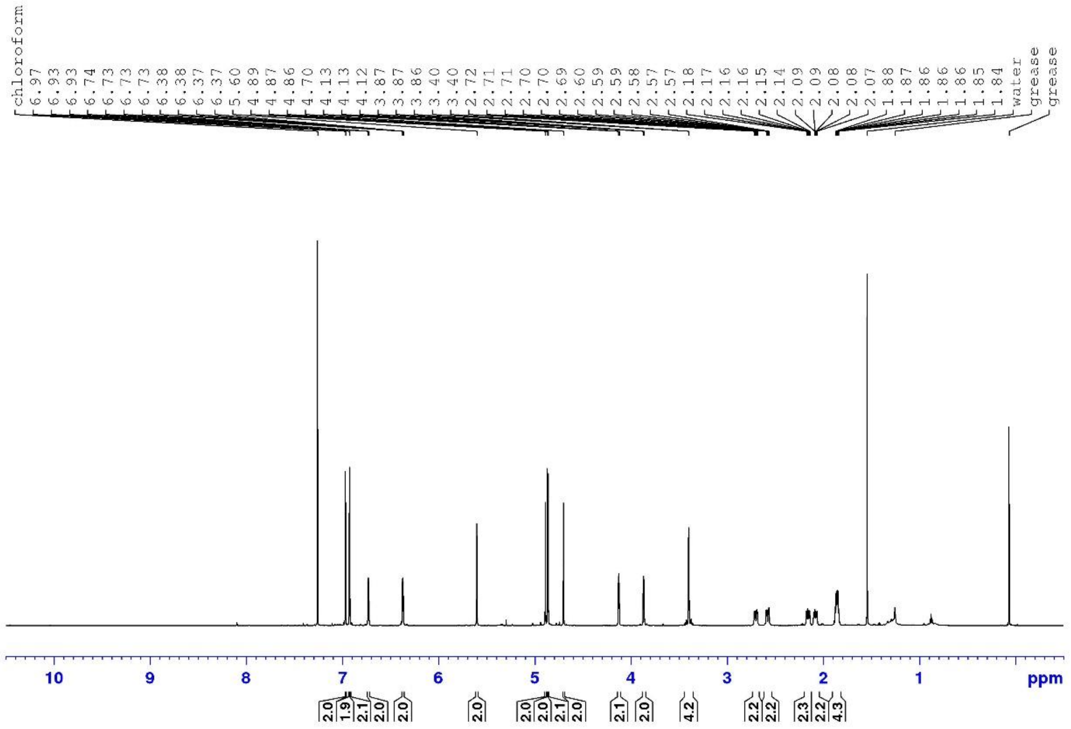

Figure S35. ^{1}H NMR of **11b** in $CDCl_3$ at a 700 MHz spectrometer at 298 K, overview. (10.5 ppm- -0.5 ppm).

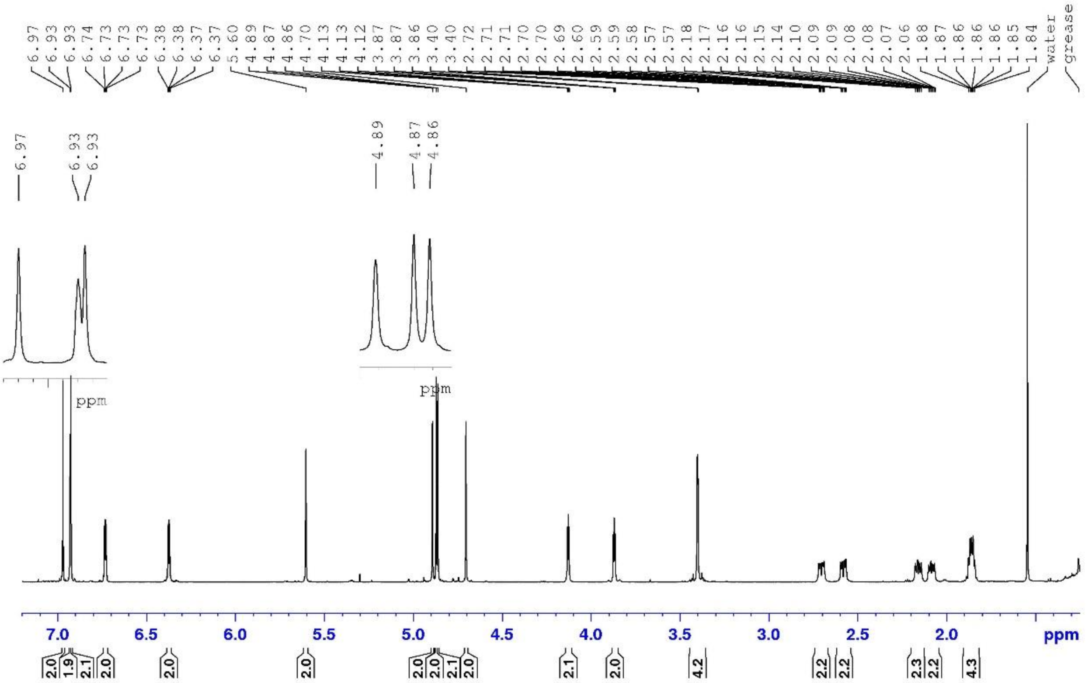

Figure S36. ^{1}H NMR of **11b** in $CDCl_3$ at a 700 MHz spectrometer at 298 K (7.2 ppm-1.3 ppm).

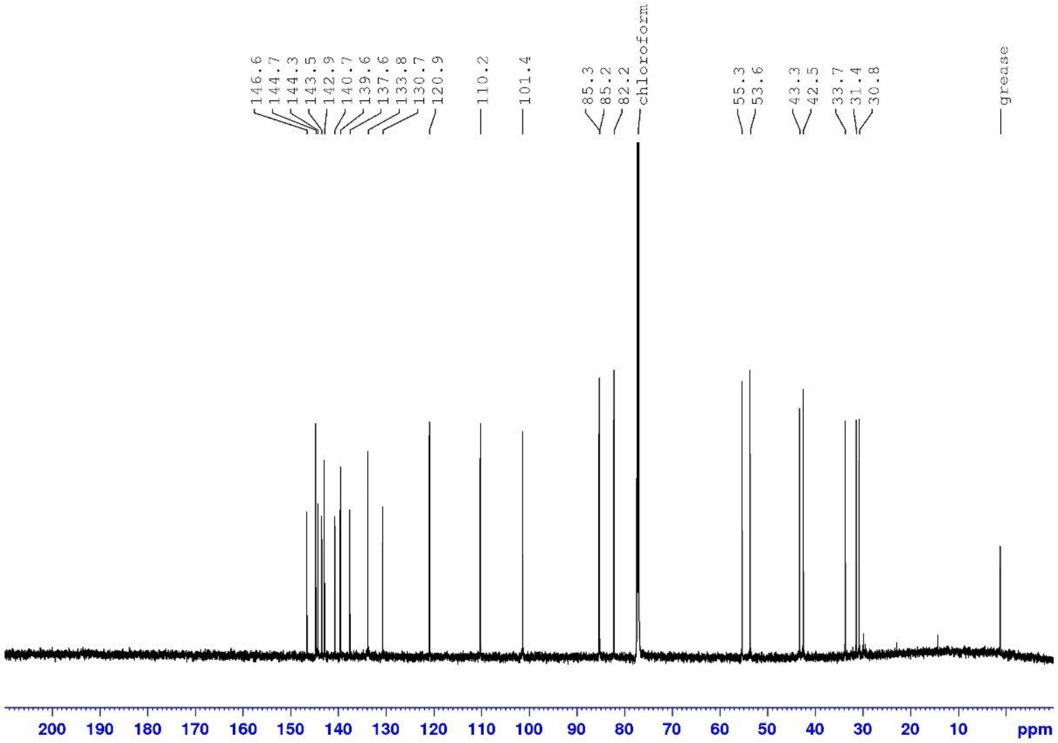

Figure S37. $^{13}C\{^{1}H\}$ NMR of **11b** in $CDCl_3$ at a 176 MHz spectrometer at 298 K, overview (210 ppm- -10 ppm).

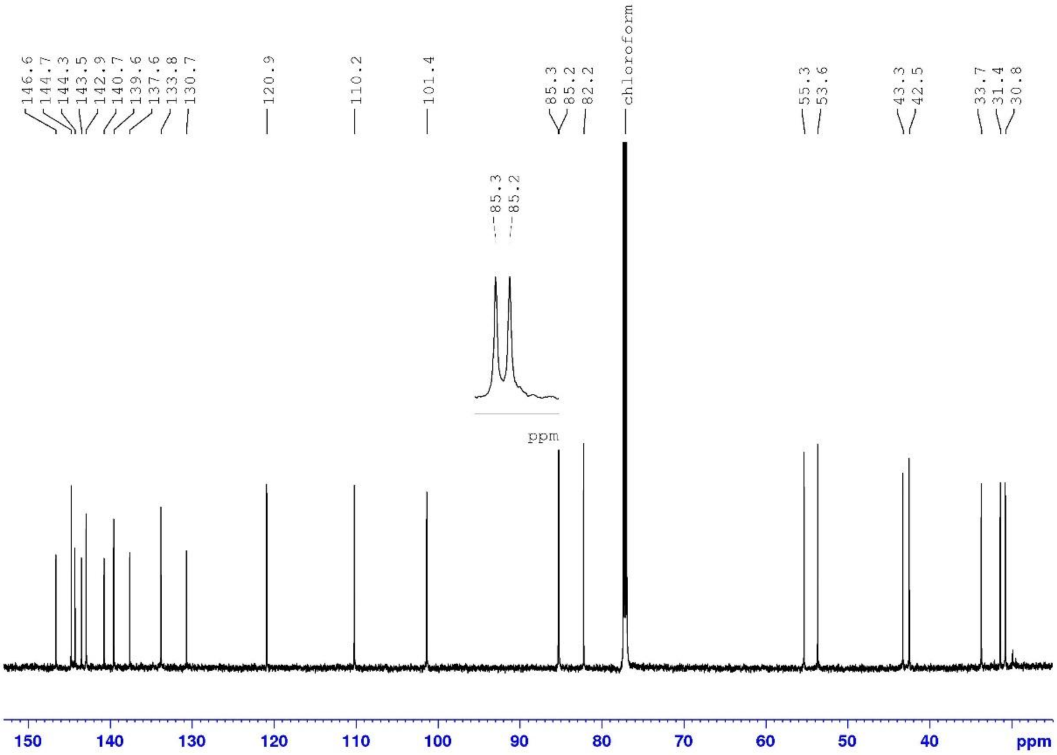

Figure S38. $^{13}C\{^{1}H\}$ NMR of **11b** in $CDCl_3$ at a 176 MHz spectrometer at 298 K (153 ppm- 25 ppm).

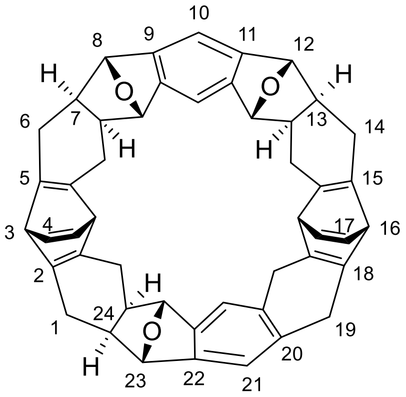

12

Table S5. ^{1}H NMR and 13C NMR (DEPT 135) data for compound **12**.

No.	δ_C	δ_H
1	31.16[a], CH_2 or 31.19[a], CH_2	H_a/ H_b: 2.57-2.48/ 2.20-2.04
2	147.5, C or 147.4, C	-
3	57.0, CH	4.14-4.12
4	139.8, CH	6.75-6.73
5	147.5, C or 147.4, C	-
6	31.16[a], CH_2 or 31.19[a], CH_2	H_a/ H_b: 2.57-2.48/ 2.20-2.04
7	46.3, CH or 45.8, CH	1.36-1.31 or 1.31-1.25 or 1.25-1.20
8	84.3, CH or 84.0, CH	4.79
9	145.2, C or 145.1, C	-
10	110.0, CH	6.85
11	145.2, C or 145.1, C	-
12	84.3, CH or 84.0, CH	4.82
13	47.2, CH	1.36-1.31 or 1.31-1.25 or 1.25-1.20
14	31.7, CH_2	H_a/ H_b: 2.57-2.48/ 2.23-2.17
15	147.8, C	-
16	56.3, CH	4.23-4.21
17	139.0, CH	6.82-6.80
18	143.3, C	-
19	34.3, CH_2	H_a/ H_b: 3.53-3.46/ 3.26-3.19
20	133.5, C	-
21	118.8, CH	6.77
22	143.7, C	-
23	84.1, CH	4.81
24	46.3, CH or 45.8, CH	1.36-1.31 or 1.31-1.25 or 1.25-1.20

[a]chemical shifts are rounded to two decimals to distinguish them.

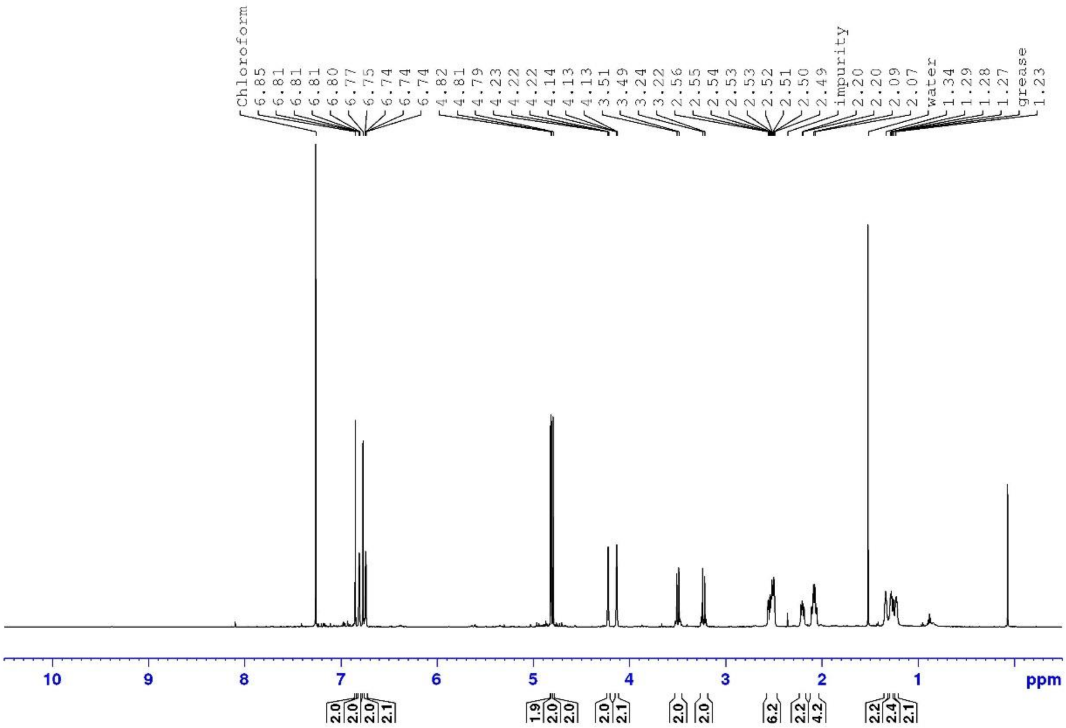

Figure S39. ^{1}H NMR of **12** in $CDCl_3$ at a 700 MHz spectrometer at 298 K, overview. (10.5 ppm- -0.5 ppm).

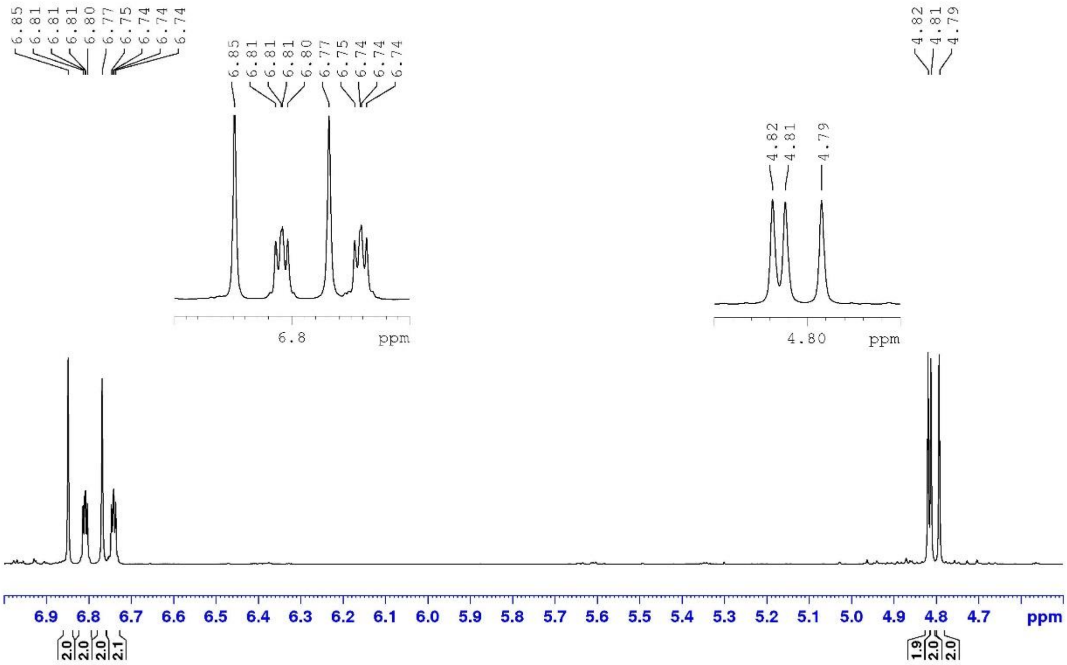

Figure S40. ^{1}H NMR of **12** in $CDCl_3$ at a 700 MHz spectrometer at 298 K, part 1 (7.0 ppm-4.5 ppm).

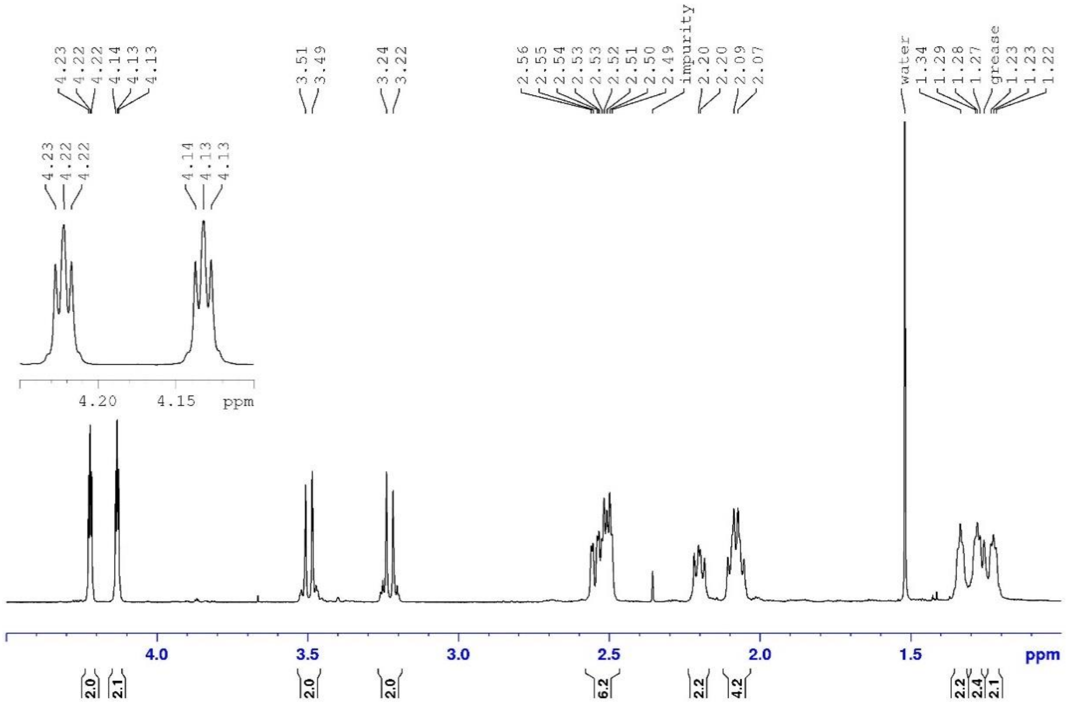

Figure S40. ^{1}H NMR of **12** in $CDCl_3$ at a 700 MHz spectrometer at 298 K, part 2 (4.5ppm-1 ppm).

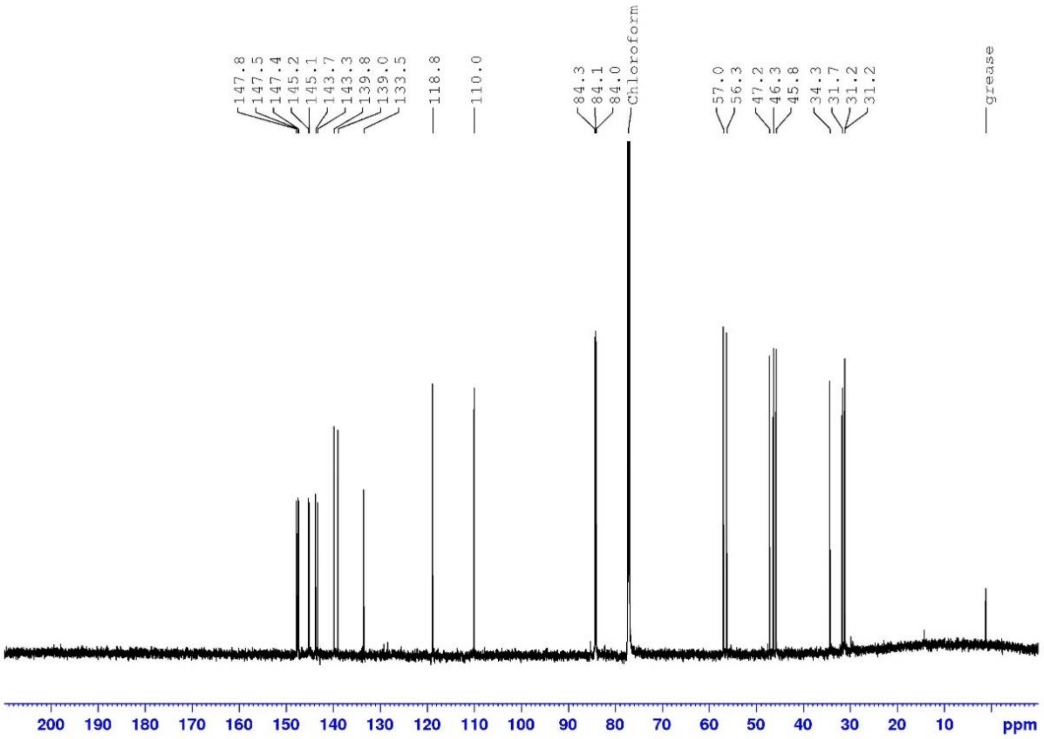

Figure S41. $^{13}C\{^{1}H\}$ NMR of **12** in $CDCl_3$ at a 176 MHz spectrometer at 298 K, overview (210 ppm--10 ppm).

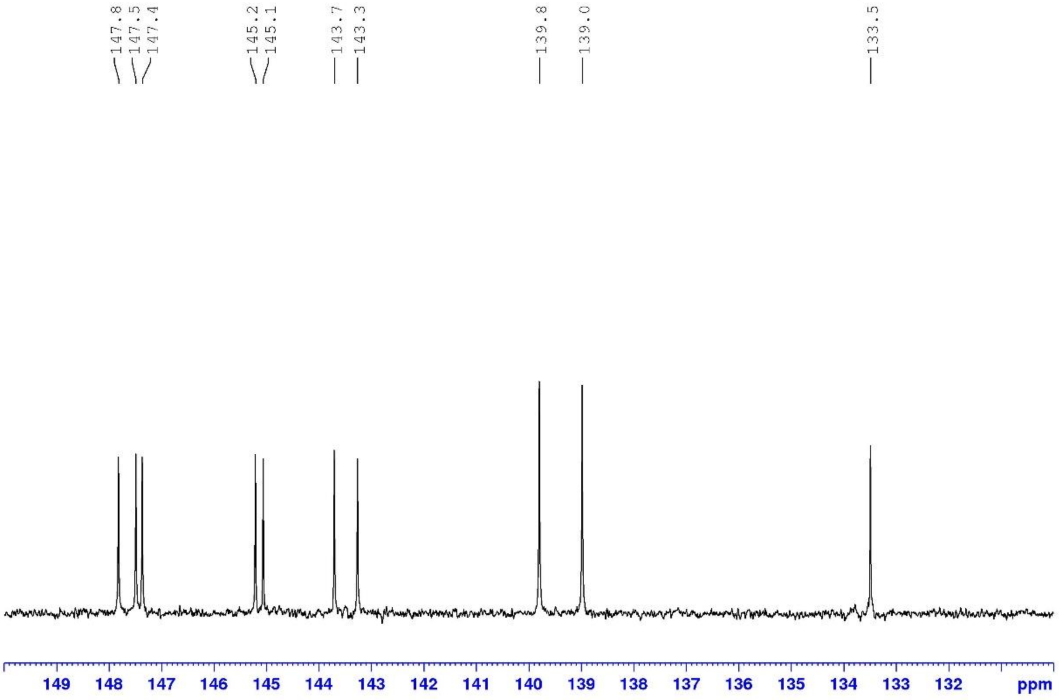

Figure S42. $^{13}C\{^{1}H\}$ NMR of **12** in $CDCl_3$ at a 176 MHz spectrometer at 298 K, part 1 (150 ppm- 130 ppm).

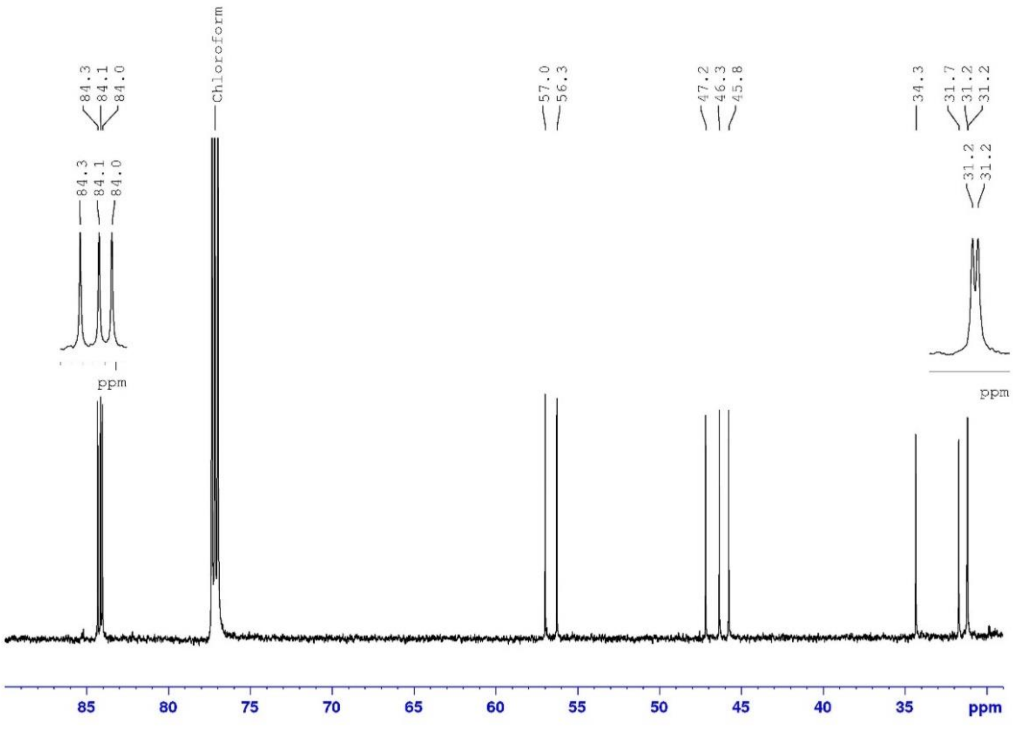

Figure S43. $^{13}C\{^{1}H\}$ NMR of **12** in $CDCl_3$ at a 176 MHz spectrometer at 298 K, part 2 (90 ppm- 29 ppm).

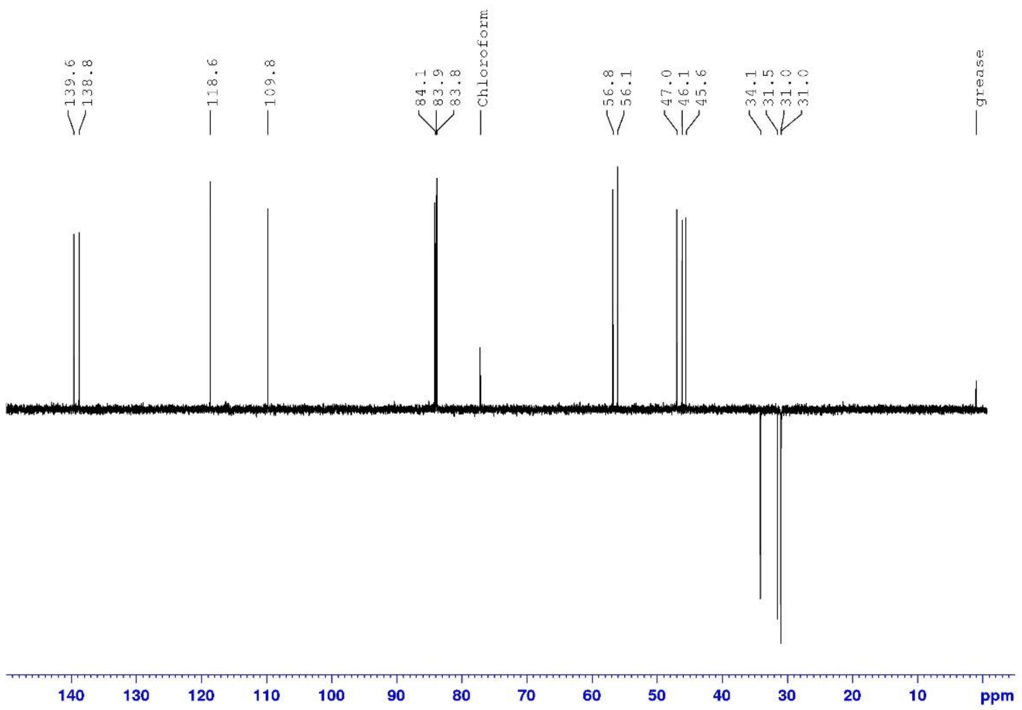

Figure S44. DEPT 135 NMR of **12** in $CDCl_3$ at a 176 MHz spectrometer at 298 K (150 ppm- -5 ppm).

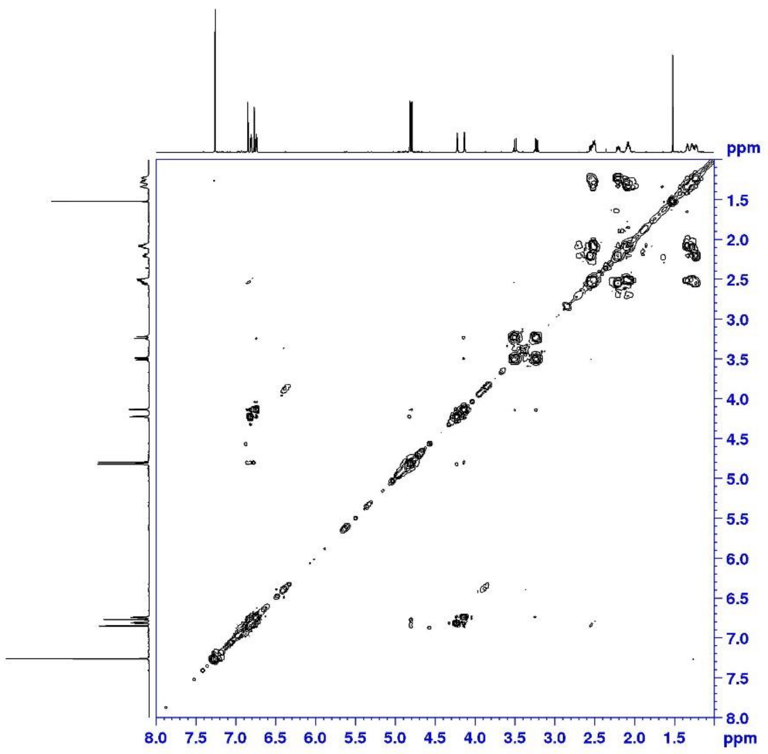

Figure S45. $^1H,^1H$ COSY NMR spectrum of **12** in $CDCl_3$ at a 700 MHz spectrometer at 298 K.

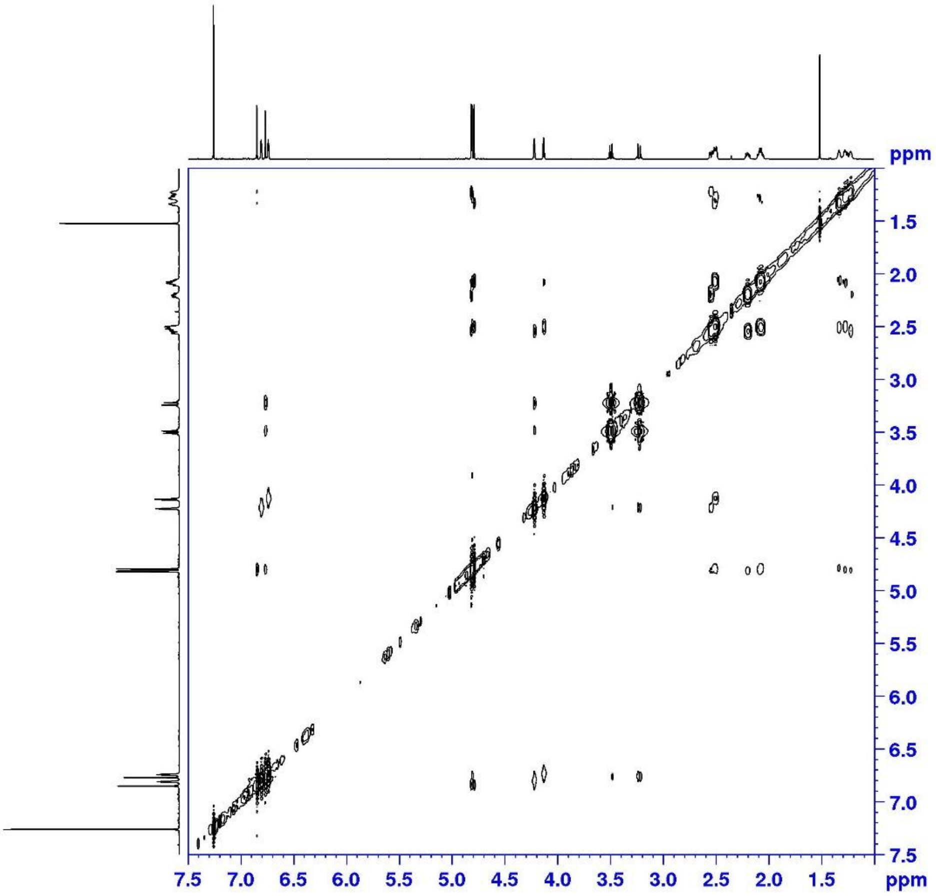

Figure S46. ^{1}H,^{1}H NOESY NMR spectrum of **12** in $CDCl_3$ at a 700 MHz spectrometer at 298 K.

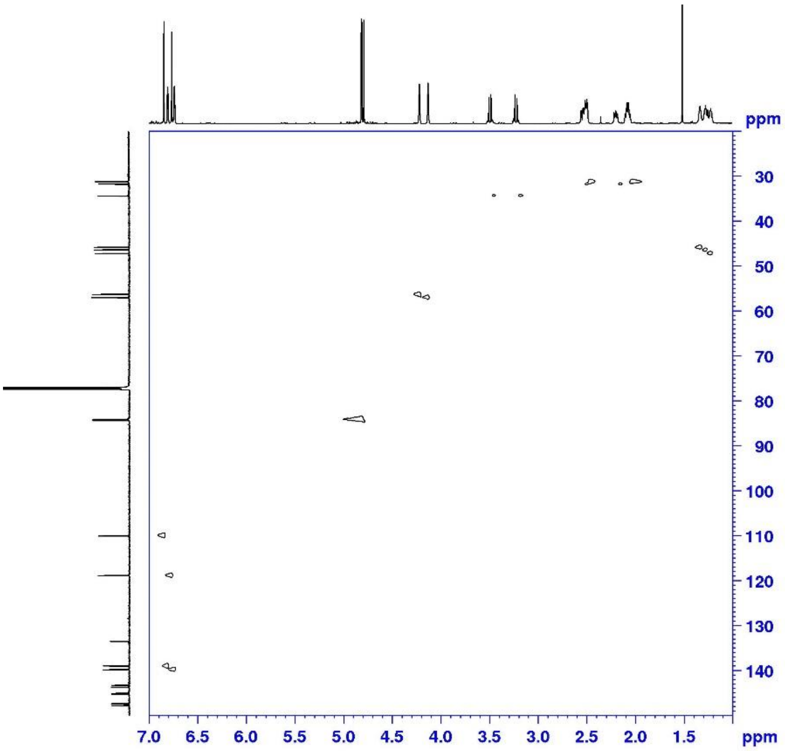

Figure S47. ^{1}H,^{13}C HSQC NMR spectrum of **12** in $CDCl_3$ at a 700 MHz spectrometer at 298 K.

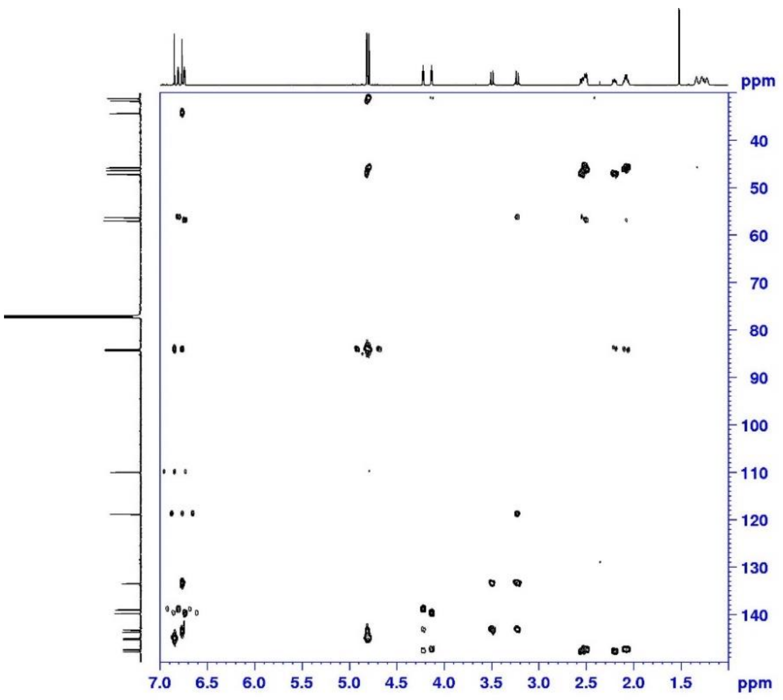

Figure S48. $^{1}H,^{13}C$ HMBC NMR spectrum of **12** in $CDCl_3$ at a 700 MHz spectrometer at 298 K.

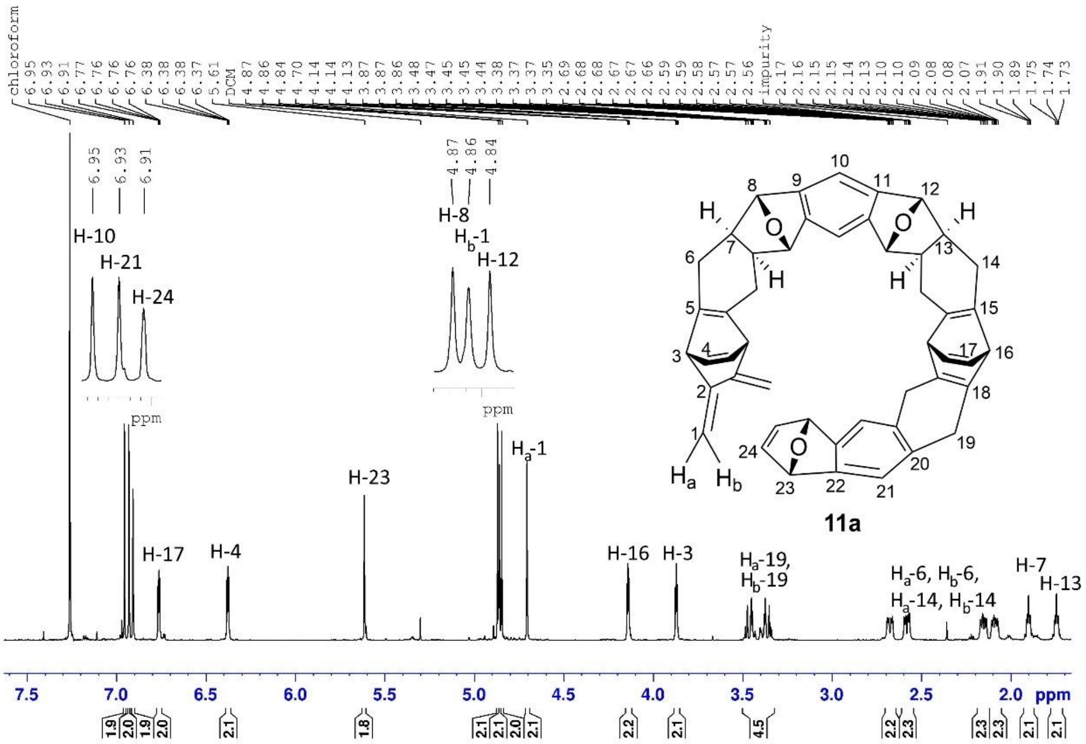

Figure S49. ^{1}H-NMR (700 MHz) spectra and numbering of one set of chemically equivalent proton and carbon atoms of **11a.** The NMR was measured in $CDCl_3$ with the corresponding assignment next to the signals. The ranges 7.00-6.90 ppm and 4.90-4.80 are extended.

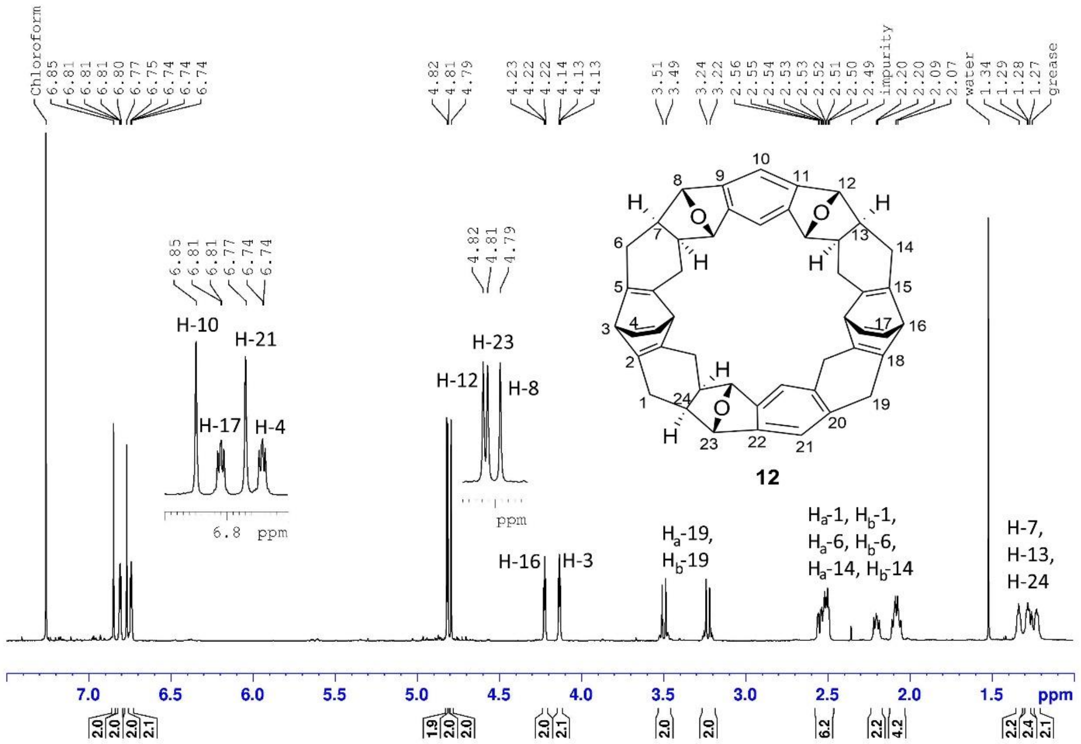

Figure S50. ^{1}H-NMR (700 MHz) spectra and numbering of one set of chemically equivalent proton and carbon atoms of **12.** The NMR was measured in $CDCl_3$ with the corresponding assignment next to the signals. The ranges 6.90-6.70 ppm and 4.85-4.80 are extended.

2. ESI/APCI High Resolution Mass Spectra

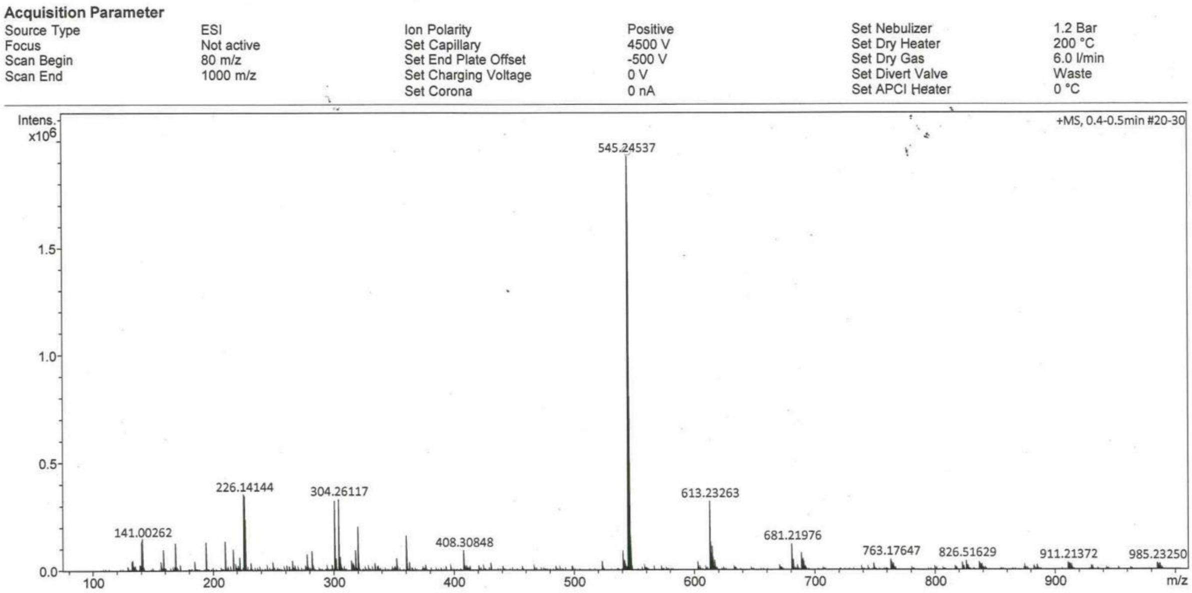

Figure S51. HRMS (APCI) of **6a**.

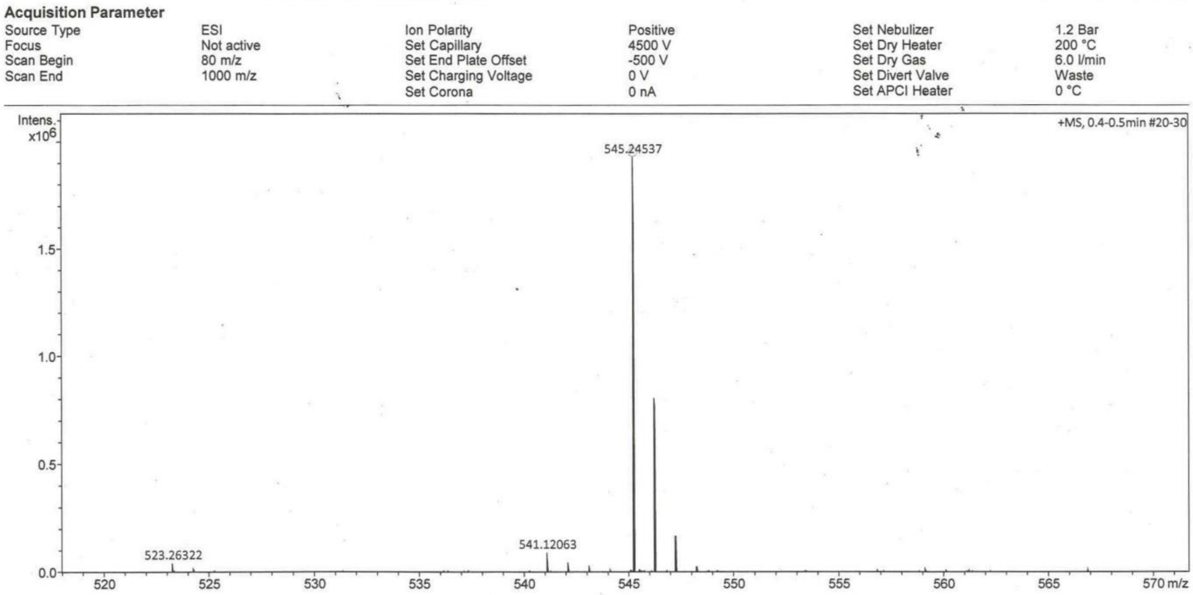

Figure S52. HRMS (APCI) of **6a**.

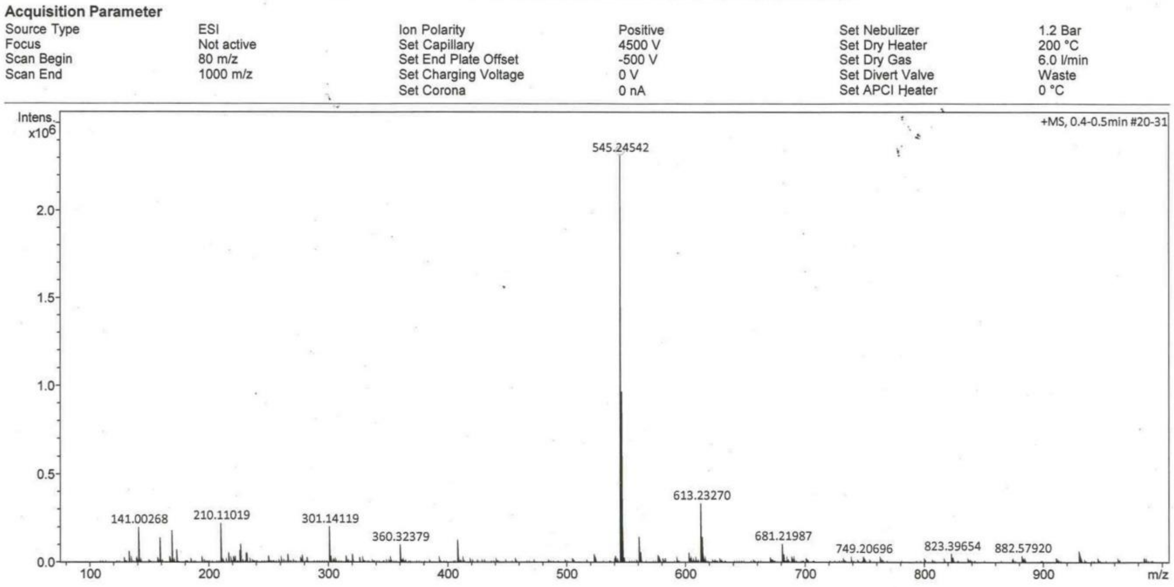

Figure S53. HRMS (APCI) of **6b**.

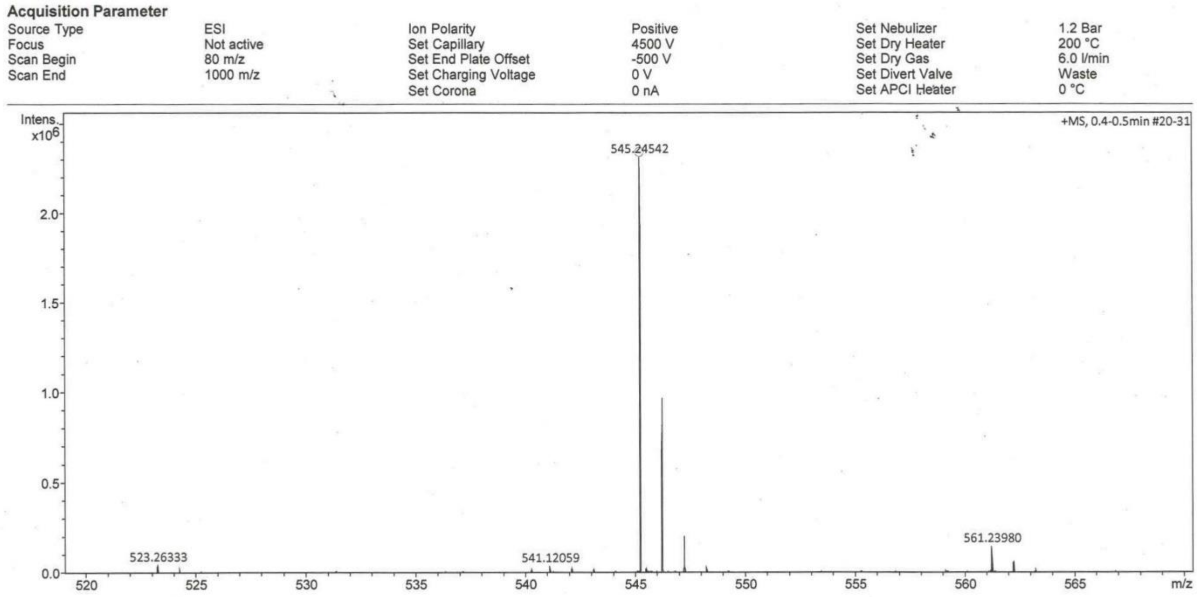

Figure S54. HRMS (APCI) of **6b**.

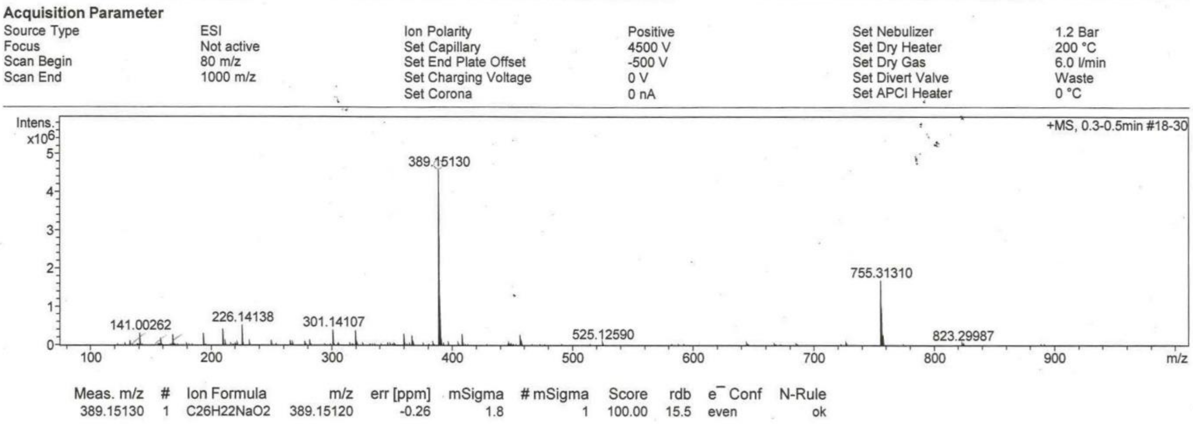

Figure S55. HRMS (APCI) of **4a**.

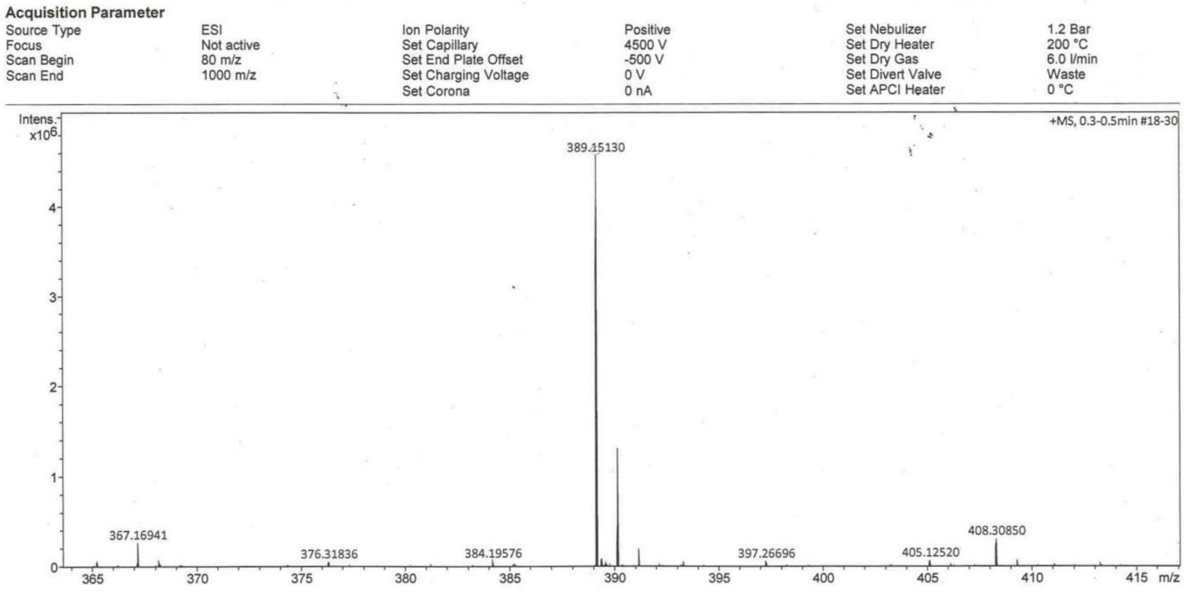

Figure S56. HRMS (APCI) of **4a**.

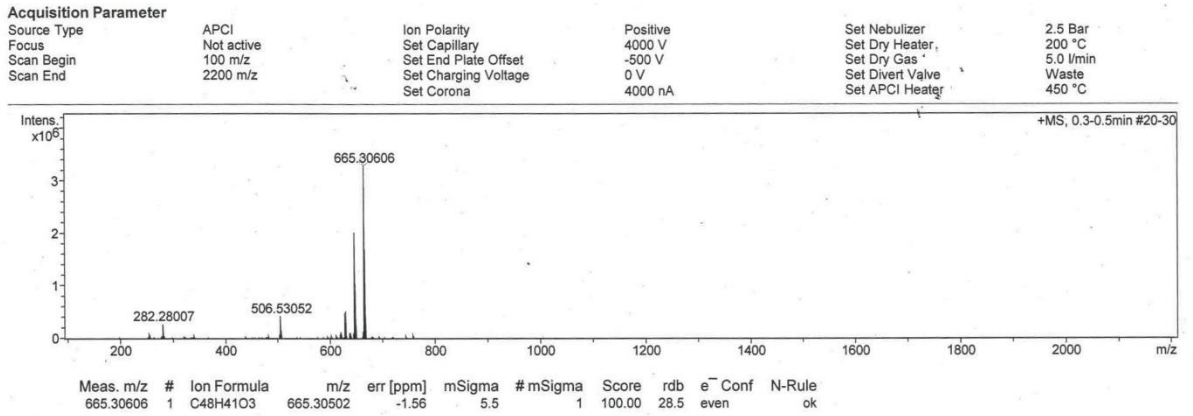

Figure S57. HRMS (APCI) of **11a**.

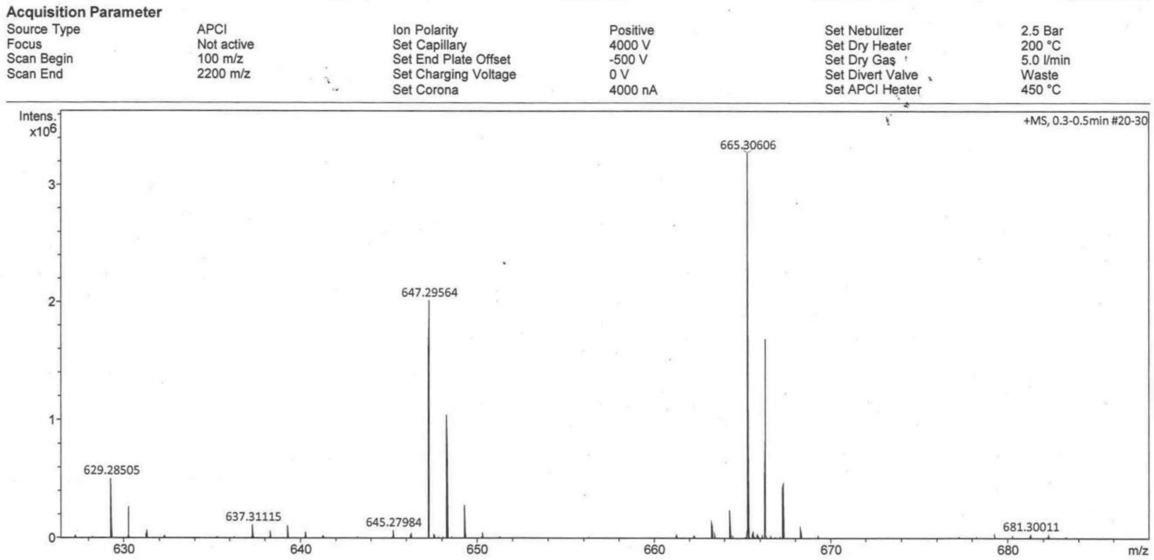

Figure S58. HRMS (APCI) of **11a**.

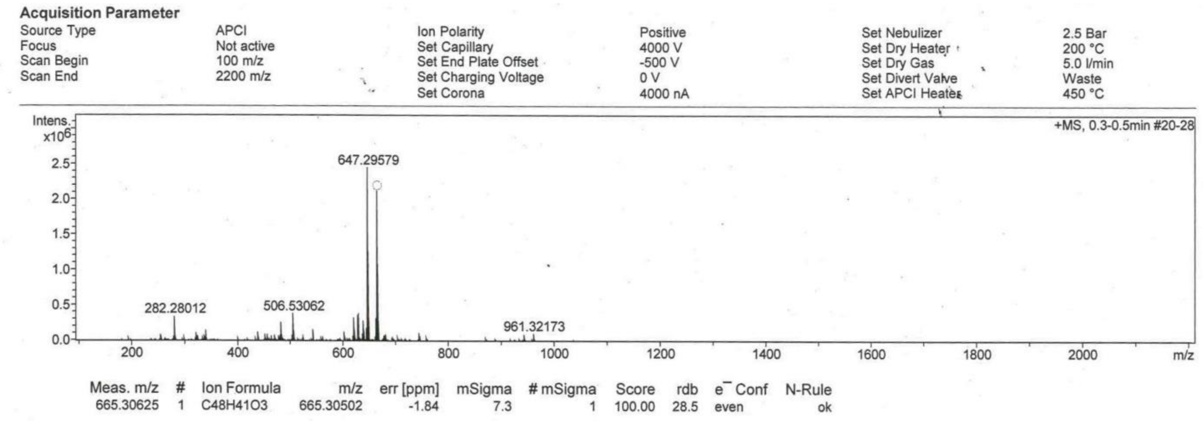

Figure S59. HRMS (APCI) of **11b**.

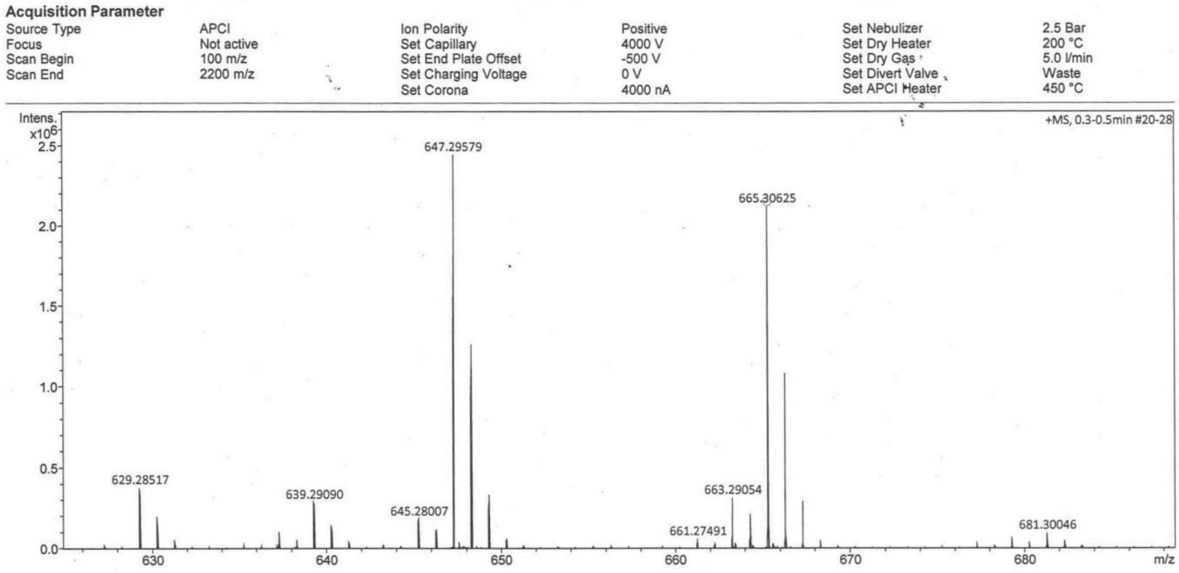

Figure S60. HRMS (APCI) of **11b**.

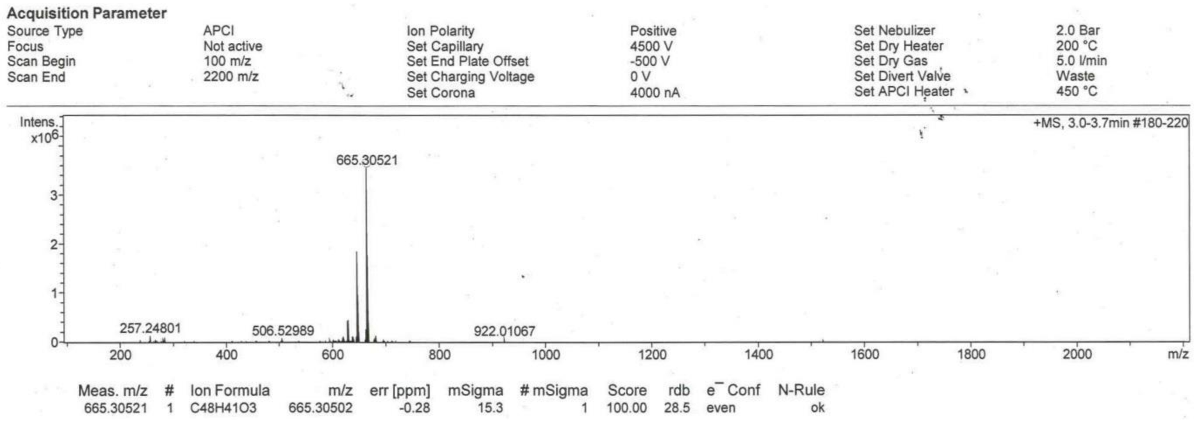

Figure S61. HRMS (APCI) of **12**.

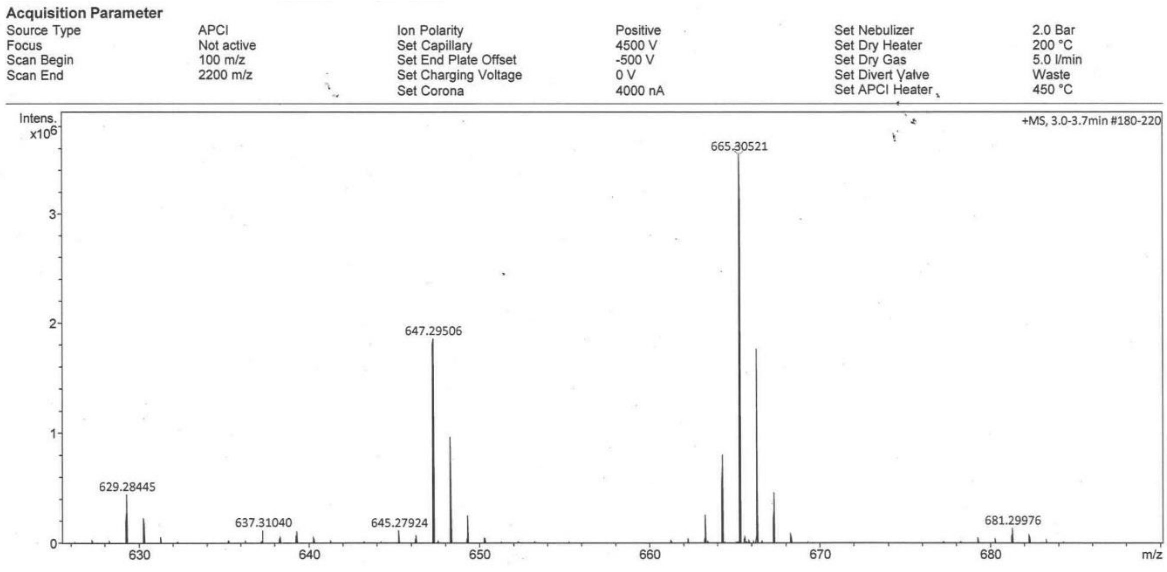

Figure S62. HRMS (APCI) of **12**.

3. HPLC-MS

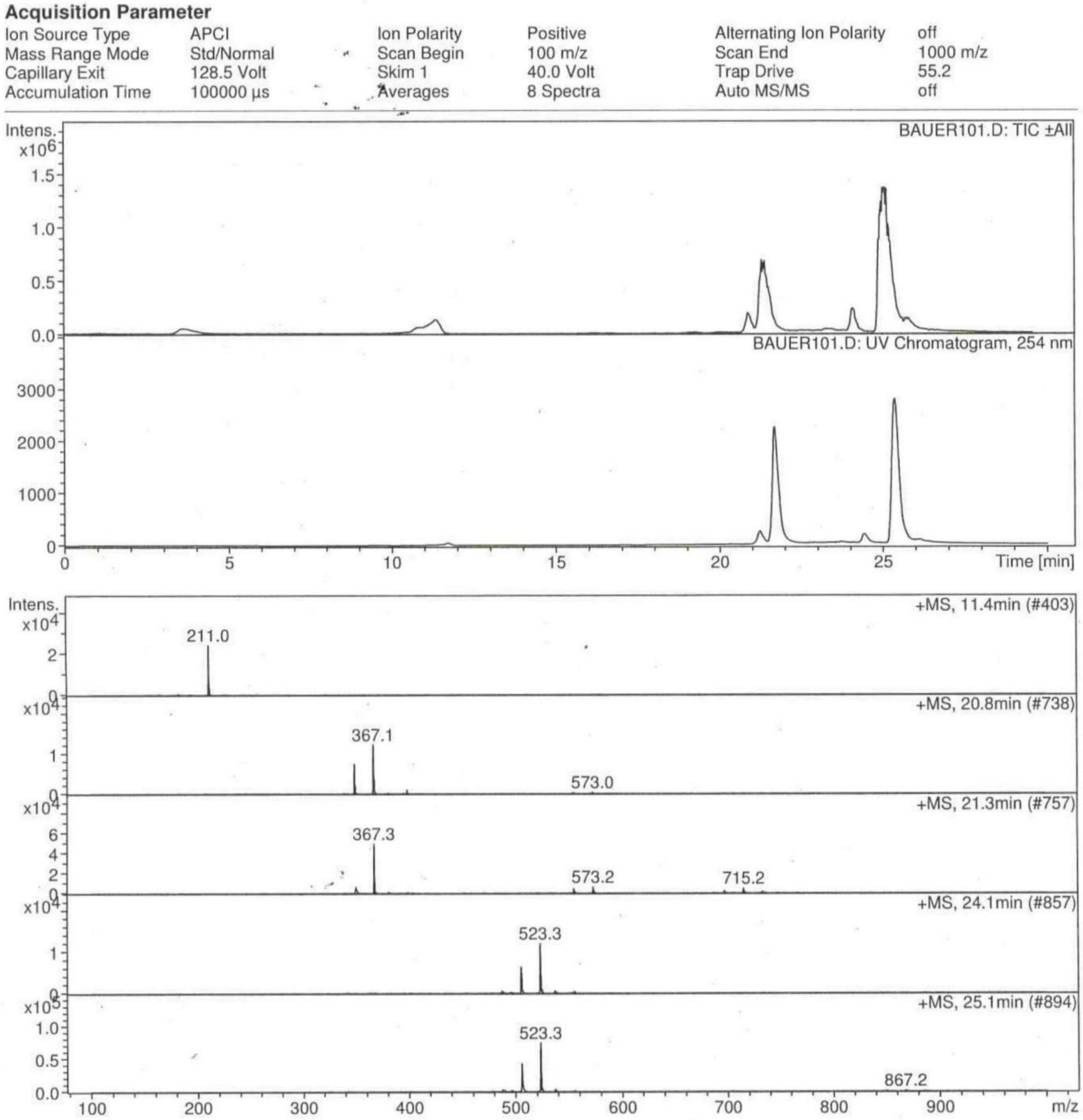

Figure S63. HPLC-MS (APCI) of the crude product for the following reaction: equimolar ratio of **1b** and **2**, toluene, reflux, 18 h.

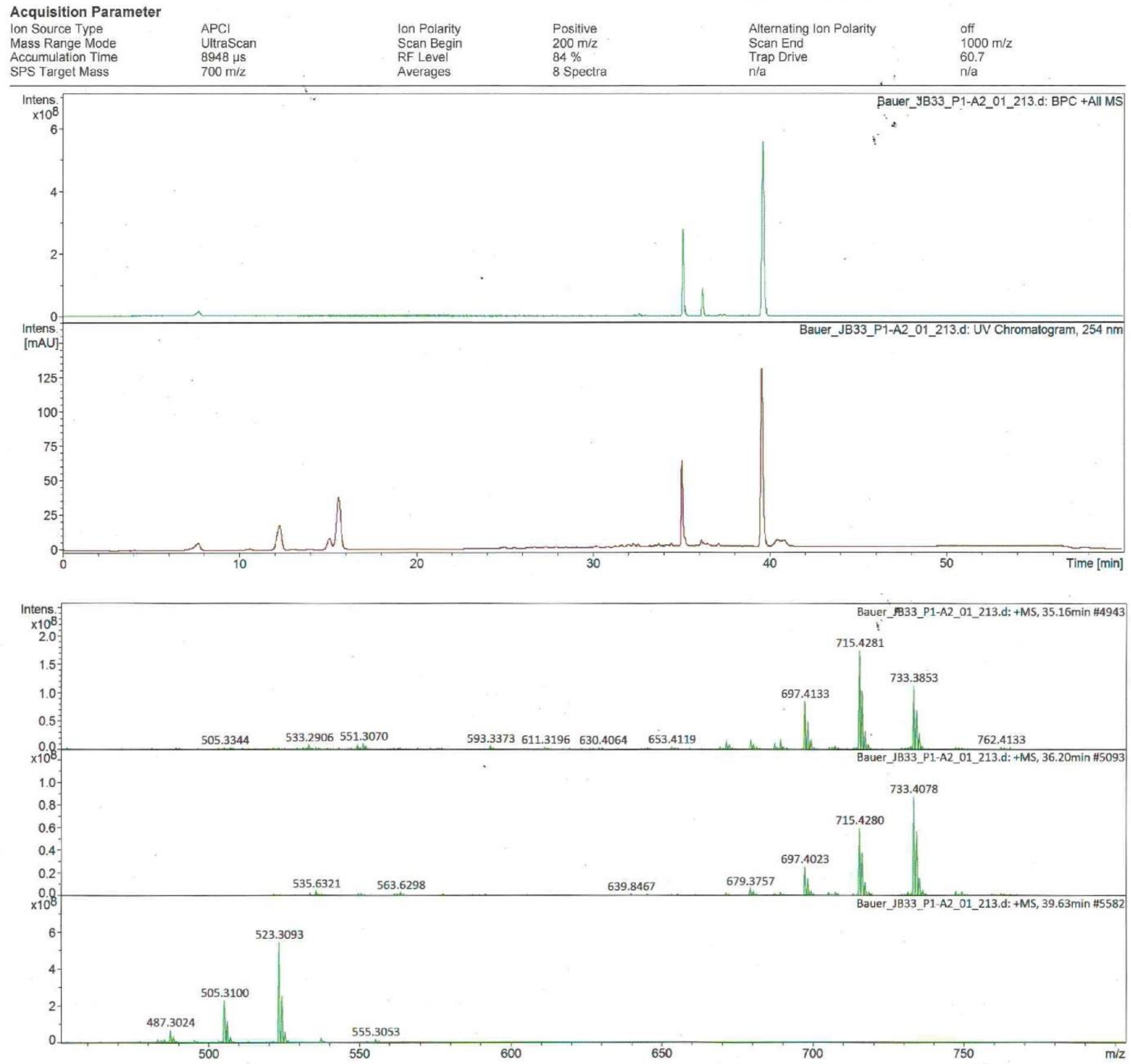

Figure S64. HPLC-MS (APCI) of the crude product for the following reaction: autoclave: **2**, **6a** toluene, 120°C, 168 h, 85 bar.

4. Computational Details

Due to the size of the systems, London dispersion interactions are deemed important in the transition states for Diels-Alder reactions. We have therefore performed computations for formation of isomers **3a-d** and **4a-d** using the 6-311+G** basis set and three different density functional methods. These were M062X [1], B3LYP [2,3], and B3LYP-D3BJ employing London dispersion correction along with Becke-Johnson damping as introduced by Grimme [4,5]. While B3LYP does not account for London dispersion, D3BJ adds a pairwise correction to the B3LYP energy. The highly parameterized M062X functional was constructed to account for London dispersion to some degree. In addition, we have employed the B3LYP/6-311+G** geometries for subsequent single point energy evaluations using the DLPNO-CCSD(T) method [6-8] with tightpno [9] settings in conjunction with the cc-pVTZ basis set and the appropriate fitting basis set [10,11]. The DLPNO-CCSD(T) method was shown to perform very well in recent large scale benchmark tests [12].

The data (see Table S6) show that the B3LYP functional underestimates the stability of the 1:1 cycloaddition products **3** and **4** with respect to the separated reactants and at the same time overestimates the barriers for their formation compared to DLPNO-CCD(T). Including London dispersion via the B3LYP-D3BJ scheme improved the performance significantly, while the best agreement was obtained with the M062X functional in this case. We therefore chose M062X for the present study and computed energies of other compounds and the barriers for their formation only with this functional.

Although the DA reaction is not very sensitive to solvent polarity, we took into account the effect of the toluene solvent in our computations (see Table S7). For this purpose the polarizable continuum model using the integral equation formalism variant (IEFPCM) as implemented in Gaussian 16 was employed [13]. The geometries were optimized and harmonic vibrational frequencies were computed modelling toluene as solvent.

Table S6. Differences of electronic energies (ΔE_{el}), zero-point vibrational corrected energies (ΔE_0), enthalpy ($\Delta H°$) and Gibbs free energy ($\Delta G°$) both at T = 298.15 K in kcal mol^{-1} with respect to separated reactants **1a** + **2** and **1b** + **2** for formation of isomers **3** and **4**, respectively, as computed at various levels of theory.

	B3LYP/6-311+G**				B3LYP-D3BJ/6-311+G**				M062X/6-311+G**				DLPNO-CCSD(T)[a]	
	ΔE_{el}	ΔE_0	$\Delta H°$	$\Delta G°$	ΔE_{el}	ΔE_0	$\Delta H°$	$\Delta G°$	ΔE_{el}	ΔE_0	$\Delta H°$	$\Delta G°$	ΔE_{el}	$\Delta G°$ [b]
**3a**	-39.3	-34.0	-34.9	-20.1	-48.4	-42.9	-43.8	-29.0	-54.1	-48.7	-49.6	-34.6	-52.5	-33.2
**3b**	-39.1	-33.7	-34.6	-19.7	-51.4	-45.7	-46.6	-31.4	-55.5	-49.9	-50.8	-35.3	-53.7	-34.3
**3c**	-40.0	-34.7	-35.5	-20.7	-48.7	-43.2	-44.1	-29.2	-54.3	-49.0	-49.9	-35.0	-53.2	-33.9
**3d**	-39.0	-33.6	-34.5	-19.7	-52.5	-46.9	-47.9	-32.6	-57.2	-51.9	-52.9	-37.2	-54.7	-35.4
TS-**3a**	18.6	19.8	19.5	33.4	6.6	8.1	7.7	21.9	8.9	10.2	9.8	24.2	10.9	25.7
TS-**3b**	27.3	28.4	28.1	42.0	14.9	16.3	15.9	30.0	19.0	20.2	19.8	34.0	20.1	34.8
TS-**3c**	19.8	21.1	20.8	34.6	10.0	11.4	11.0	25.0	12.1	13.6	13.1	27.3	13.5	28.2
TS-**3d**	25.7	26.9	26.5	40.5	11.3	12.6	12.2	26.7	15.3	16.5	16.0	30.5	17.2	32.0
**4a**	-42.7	-37.6	-38.4	-23.4	-51.5	-46.3	-47.1	-32.2	-56.7	-51.6	-52.5	-37.4	-55.5	-36.2
**4b**	-41.6	-36.3	-37.2	-22.1	-54.2	-48.6	-49.6	-33.7	-57.8	-52.4	-53.4	-37.5	-55.5	-36.0
**4c**	-42.7	-37.6	-38.4	-23.4	-51.4	-46.2	-47.0	-31.9	-56.6	-51.5	-52.4	-37.1	-55.5	-36.1
**4d**	-41.5	-36.2	-37.1	-22.1	-51.9	-46.5	-47.4	-32.6	-56.1	-50.8	-51.7	-36.5	-55.1	-35.7
TS-**4a**	17.5	18.6	18.3	32.3	5.9	7.2	6.8	21.1	8.4	9.7	9.2	23.8	10.1	24.9
TS-**4b**	25.7	26.7	26.4	40.4	20.3	13.7	13.3	27.7	16.7	17.9	17.4	31.8	18.0	32.8
TS-**4c**	18.4	19.5	19.2	33.2	7.8	9.1	8.7	22.9	10.1	11.4	11.0	25.4	11.5	26.3
TS-**4d**	24.8	25.8	25.5	39.6	10.5	11.8	11.4	25.9	14.8	16.0	15.6	30.1	16.5	31.3

[a] Single point using the cc-pVTZ basis set based on the B3LYP/6-311+G** geometries. [b] The Gibbs free energy correction was determined at the B3LYP/6-311+G** and added to the DLPNO-CCSD(T)/cc-pVTZ electronic energy.

All DFT computations were performed with the Gaussian 16 program [14], while the DLPNO-CCSD(T) employed Orca 4.2.1 [15,16].

Table S7. Differences of electronic energy (ΔE_{el}), zero-point vibrational corrected energy (ΔE_0), enthalpy ($\Delta H°$) and Gibbs free energy ($\Delta G°$) both at T = 298.15 K in kcal mol^{-1} as computed at the M062X/6-311+G**/toluene level of theory. The energy values are given with respect to separated reactants **1a** + **2** and **1b** + **2** for formation of isomers **3** and **4**, respectively, those of **5** and **6** are given relative to **1a** + **3a** and **1b** + **4a**, respectively, while the energy values of formation of **A** and **B** are given with respect to **3a** + **2** and **4a** + **2**, respectively.

**Structure**	ΔE_{el}	ΔE_0	$\Delta H°$	$\Delta G°$
**3a**	-53.1	-47.8	-48.7	-34.1
**3b**	-54.7	-48.9	-50.0	-34.2
**3c**	-53.5	-48.1	-49.0	-34.5
**3d**	-56.1	-50.6	-51.6	-36.0
TS-**3a**	9.9	11.2	10.8	24.8
TS2-**3a**	54.6	53.8	54.2	66.3
TS-**3b**	19.6	20.9	20.5	34.2
TS2-**3b**	34.8	36.4	35.8	50.8
TS-**3c**	13.0	14.4	14.0	27.8
TS2-**3c**	15.0	16.3	15.8	30.0
TS-**3d**	16.0	17.2	16.8	30.9
TS2-**3d**	22.9	24.0	23.5	37.9
**4a**	-55.7	-50.5	-50.8	-37.3
**4b**	-57.0	-51.3	-51.9	-36.4
**4c**	-55.6	-50.4	-50.7	-36.9
**4d**	-55.1	-49.8	-50.2	-36.6
TS-**4a**	9.5	10.9	11.0	23.8
TS2-**4a**	15.6	16.8	16.9	29.5
TS-**4b**	17.4	18.6	18.7	31.3
TS2-**4b**	32.1	33.8	33.7	48.1
TS-**4c**	11.1	12.5	12.6	25.6
TS2-**4c**	13.5	14.8	14.8	28.5
TS-**4d**	15.6	16.9	17.0	30.0
TS2-**4d**	25.0	26.5	26.5	40.6
**5**	-52.9	-47.7	-48.6	-34.4
TS-**3a-5**	9.9	11.2	10.8	24.7
**6a**	-55.5	-50.3	-50.7	-36.8
**6b**	-55.4	-50.2	-50.6	-36.4
TS-**4a-6a**	9.5	10.9	11.0	24.3
TS-**4a-6b**	11.0	12.5	12.6	25.8

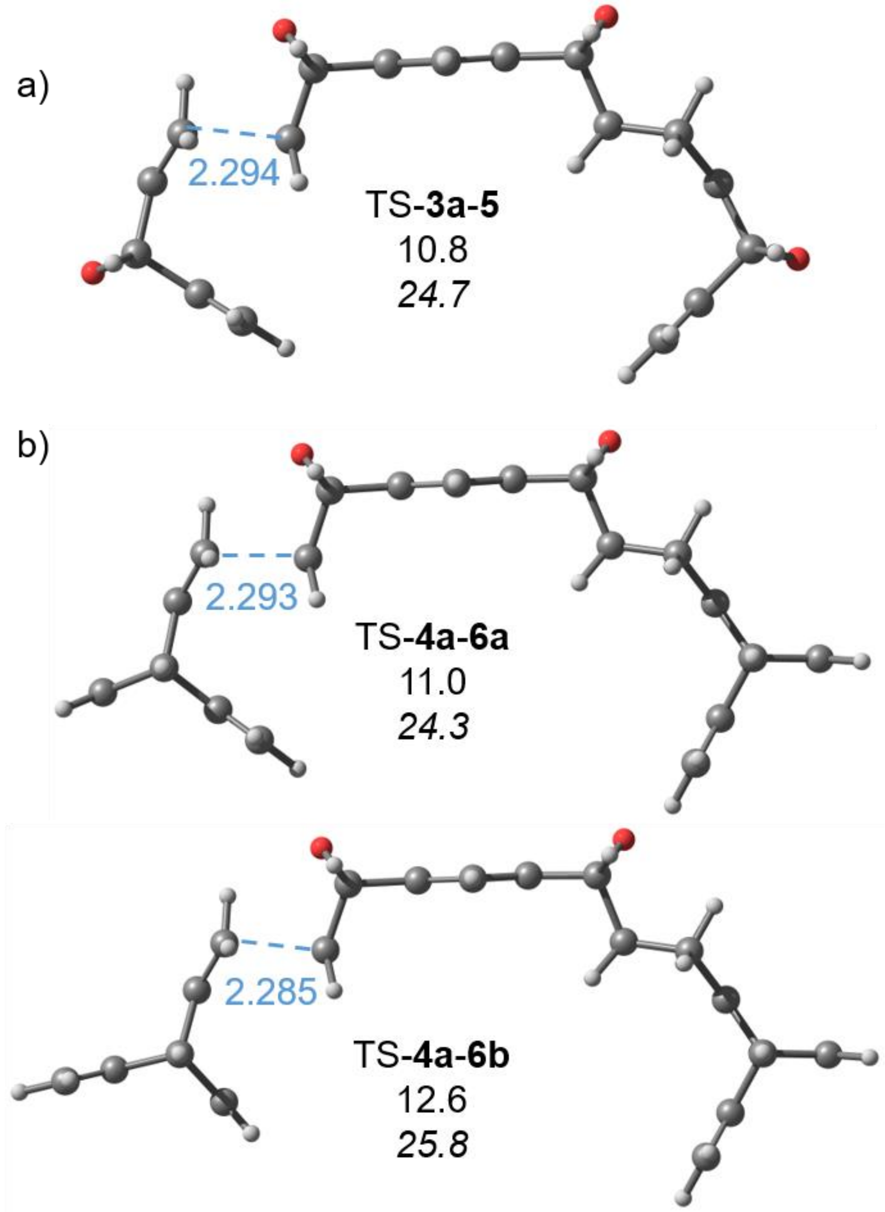

Figure S65. Transition states for the formation of 2:1 cycloaddition adducts **5** (a) and **6** (b) as computed at the M062X/6-311+G**/toluene level of theory. Distances between reacting carbon atoms are given in Å (blue), enthalpies (normal print) and free energies (italics) at 298.15 K are given in kcal mol^{-1}.

Cartesian Coordinates

All coordinates were obtained after full geometry optimization including solvent corrections at the M062X/6-311+G** level of theory and are given in Å.

21

bisdiene **1a**

C	0.000000000	1.077665000	0.746991000
C	1.228576000	0.743112000	-0.089861000
C	1.228576000	-0.743112000	-0.089861000
C	0.000000000	-1.077665000	0.746991000
C	-1.228576000	-0.743112000	-0.089861000
C	-1.228576000	0.743112000	-0.089861000
C	2.078178000	1.592644000	-0.654984000
C	2.078178000	-1.592644000	-0.654983000
C	-2.078178000	1.592644000	-0.654983000
C	-2.078178000	-1.592644000	-0.654984000
O	0.000000000	0.000000000	1.697907000
H	0.000000000	2.045167000	1.240820000
H	0.000000000	-2.045167000	1.240820000
H	2.928695000	1.243582000	-1.229828000
H	1.947076000	2.664233000	-0.558301000
H	1.947076000	-2.664233000	-0.558301000
H	2.928695000	-1.243582000	-1.229828000
H	-2.928695000	1.243582000	-1.229828000
H	-1.947076000	2.664233000	-0.558301000
H	-2.928695000	-1.243582000	-1.229828000
H	-1.947076000	-2.664233000	-0.558301000

24

bisdiene **1b**

C	-0.000008000	-1.288048000	0.469766000
C	-1.224439000	-0.743543000	-0.261968000
C	-1.224426000	0.743559000	-0.261972000
C	0.000008000	1.288048000	0.469766000
C	1.224439000	0.743543000	-0.261968000
C	1.224426000	-0.743559000	-0.261972000
C	-2.142678000	-1.530949000	-0.818257000
C	-2.142646000	1.530977000	-0.818277000
C	2.142646000	-1.530976000	-0.818277000
C	2.142678000	1.530949000	-0.818257000
C	-0.000004000	-0.665839000	1.854275000
C	0.000004000	0.665838000	1.854275000
H	-0.000015000	-2.376308000	0.486244000
H	0.000015000	2.376308000	0.486244000
H	-3.006923000	-1.127000000	-1.332984000
H	-2.051830000	-2.609971000	-0.773703000
H	-2.051781000	2.609997000	-0.773728000
H	-3.006891000	1.127038000	-1.333012000
H	2.051781000	-2.609997000	-0.773728000
H	3.006891000	-1.127038000	-1.333012000
H	3.006923000	1.127000000	-1.332984000
H	2.051830000	2.609971000	-0.773703000
H	-0.000008000	-1.280393000	2.745780000
H	0.000008000	1.280393000	2.745780000

26

bis-dienophile **2**

C	-3.200113000	-0.664022000	1.139188000
C	-3.200113000	0.664021000	1.139188000
C	-2.664139000	1.068234000	-0.247790000
C	-1.174707000	0.697292000	-0.260424000
C	-1.174707000	-0.697291000	-0.260424000
C	-2.664139000	-1.068234000	-0.247790000
C	0.000000000	1.436879000	-0.253433000
C	1.174707000	0.697292000	-0.260424000
C	1.174707000	-0.697291000	-0.260424000
C	0.000000000	-1.436879000	-0.253434000
C	2.664139000	1.068234000	-0.247790000
C	3.200113000	0.664021000	1.139188000
C	3.200113000	-0.664022000	1.139188000

C	2.664139000	-1.068234000	-0.247790000
O	-3.189236000	0.000000000	-1.049041000
O	3.189236000	0.000000000	-1.049041000
H	-3.438180000	-1.348706000	1.939807000
H	-3.438182000	1.348705000	1.939808000
H	-2.944831000	2.040953000	-0.639665000
H	-2.944830000	-2.040953000	-0.639665000
H	0.000000000	2.521367000	-0.259041000
H	0.000000000	-2.521367000	-0.259043000
H	2.944831000	2.040953000	-0.639665000
H	3.438182000	1.348706000	1.939807000
H	3.438180000	-1.348706000	1.939808000
H	2.944830000	-2.040954000	-0.639665000

47
3a

C	-4.809535000	2.195137000	0.664105000
C	-4.809532000	2.195146000	-0.664079000
C	-5.008486000	0.722526000	-1.068725000
C	-3.708253000	-0.004544000	-0.699787000
C	-3.708255000	-0.004554000	0.699787000
C	-5.008491000	0.722510000	1.068730000
C	-2.680517000	-0.564766000	-1.436233000
C	-1.656510000	-1.154537000	-0.695928000
C	-1.656512000	-1.154545000	0.695919000
C	-2.680520000	-0.564784000	1.436228000
C	-0.348154000	-1.820633000	-1.073932000
C	0.801145000	-0.816384000	-0.783503000
C	0.801141000	-0.816387000	0.783498000
C	-0.348155000	-1.820643000	1.073917000
C	2.132788000	-1.265568000	-1.430263000
C	3.284263000	-0.700168000	-0.666959000
C	3.284259000	-0.700162000	0.666959000
C	2.132785000	-1.265567000	1.430261000
C	4.420393000	0.228964000	-1.073569000
C	3.896464000	1.632505000	-0.744345000
C	3.896459000	1.632513000	0.744329000
C	4.420385000	0.228975000	1.073570000
C	3.543927000	2.588759000	1.595250000
C	3.543930000	2.588741000	-1.595278000
O	-5.852736000	0.271094000	-0.000003000
O	-0.179678000	-2.758623000	-0.000008000
O	5.349160000	0.031925000	0.000054000
H	-4.631560000	3.011615000	1.348350000
H	-4.631555000	3.011634000	-1.348311000
H	-5.443827000	0.514189000	-2.041106000
H	-5.443835000	0.514159000	2.041106000
H	-2.678937000	-0.563011000	-2.520839000
H	-2.678943000	-0.563042000	2.520834000
H	-0.301928000	-2.325085000	-2.037001000
H	0.555808000	0.178616000	-1.158160000
H	0.555797000	0.178610000	1.158157000
H	-0.301928000	-2.325104000	2.036981000
H	2.151898000	-0.972685000	-2.482583000
H	2.187130000	-2.359990000	-1.396994000
H	2.151891000	-0.972684000	2.482581000
H	2.187132000	-2.359989000	1.396991000
H	4.891778000	0.100682000	-2.044047000
H	4.891763000	0.100702000	2.044052000
H	3.628549000	2.445075000	2.666491000
H	3.169061000	3.545452000	1.247948000
H	3.169059000	3.545436000	-1.247988000
H	3.628557000	2.445046000	-2.666517000

47
3b

C	3.492320000	-1.481495000	0.664173000
C	3.492315000	-1.481505000	-0.664157000
C	3.581716000	-0.000232000	-1.068940000
C	2.245178000	0.658881000	-0.699540000
C	2.245184000	0.658891000	0.699536000
C	3.581725000	-0.000217000	1.068934000
C	1.236228000	1.253207000	-1.435693000
C	0.240853000	1.891784000	-0.696184000
C	0.240858000	1.891791000	0.696180000

C	1.236239000	1.253224000	1.435689000
C	-0.974812000	2.717404000	-1.071167000
C	-2.253744000	1.877180000	-0.788866000
C	-2.253738000	1.877182000	0.788874000
C	-0.974806000	2.717412000	1.071163000
C	-2.290719000	0.542841000	-1.559237000
C	-2.374086000	-0.639884000	-0.665617000
C	-2.374077000	-0.639883000	0.665630000
C	-2.290700000	0.542844000	1.559247000
C	-2.269865000	-2.107175000	-1.069374000
C	-0.826134000	-2.497179000	-0.743826000
C	-0.826125000	-2.497184000	0.743818000
C	-2.269850000	-2.107175000	1.069386000
C	0.156446000	-2.774931000	1.592082000
C	0.156431000	-2.774906000	-1.592105000
O	4.397224000	0.507401000	-0.000010000
O	-1.014374000	3.668236000	-0.000005000
O	-2.970829000	-2.754921000	0.000010000
H	3.406401000	-2.309663000	1.351683000
H	3.406393000	-2.309683000	-1.351654000
H	4.002048000	0.235907000	-2.041651000
H	4.002065000	0.235935000	2.041638000
H	1.247213000	1.271921000	-2.520383000
H	1.247232000	1.271952000	2.520379000
H	-0.952247000	3.218103000	-2.036517000
H	-3.101105000	2.483918000	-1.107817000
H	-3.101099000	2.483916000	1.107831000
H	-0.952238000	3.218118000	2.036509000
H	-3.134192000	0.535331000	-2.256749000
H	-1.389790000	0.440829000	-2.179410000
H	-1.389764000	0.440834000	2.179410000
H	-3.134165000	0.535333000	2.256770000
H	-2.648760000	-2.408034000	-2.042827000
H	-2.648732000	-2.408034000	2.042844000
H	-0.003682000	-2.758759000	2.664338000
H	1.145477000	-3.035104000	1.233329000
H	1.145470000	-3.035068000	-1.233365000
H	-0.003711000	-2.758726000	-2.664358000

47

3c

C	-5.640832000	-0.664101000	1.740215000
C	-5.640831000	0.664088000	1.740220000
C	-5.519368000	1.068801000	0.259304000
C	-4.093273000	0.699954000	-0.171469000
C	-4.093274000	-0.699953000	-0.171474000
C	-5.519369000	-1.068802000	0.259295000
C	-2.969023000	1.436282000	-0.496325000
C	-1.841852000	0.695755000	-0.851979000
C	-1.841852000	-0.695750000	-0.851983000
C	-2.969023000	-1.436279000	-0.496334000
C	-0.420454000	1.073685000	-1.217580000
C	0.482582000	0.785193000	0.014662000
C	0.482580000	-0.785190000	0.014661000
C	-0.420453000	-1.073678000	-1.217585000
C	1.865302000	1.452470000	-0.125090000
C	2.896829000	0.667011000	0.612239000
C	2.896828000	-0.667012000	0.612239000
C	1.865300000	-1.452470000	-0.125088000
C	4.254787000	1.072858000	1.169512000
C	5.247292000	0.743958000	0.047871000
C	5.247292000	-0.743963000	0.047872000
C	4.254786000	-1.072862000	1.169512000
C	5.925455000	-1.595218000	-0.712165000
C	5.925453000	1.595212000	-0.712169000
O	-6.246907000	0.000002000	-0.362959000
O	-0.050929000	0.000004000	-2.095427000
O	4.519977000	0.000006000	2.082695000
H	-5.642124000	-1.348334000	2.575868000
H	-5.642124000	1.348314000	2.575879000
H	-5.899792000	2.041151000	-0.037743000
H	-5.899793000	-2.041150000	-0.037760000
H	-2.967645000	2.520880000	-0.493532000
H	-2.967646000	-2.520877000	-0.493550000
H	-0.265410000	2.037044000	-1.699136000
H	0.015295000	1.151384000	0.929628000

H	0.015290000	-1.151381000	0.929624000
H	-0.265408000	-2.037035000	-1.699144000
H	1.809700000	2.488360000	0.219356000
H	2.144811000	1.480350000	-1.187034000
H	2.144809000	-1.480354000	-1.187031000
H	1.809695000	-2.488360000	0.219361000
H	4.362301000	2.044435000	1.644459000
H	4.362298000	-2.044438000	1.644460000
H	5.834658000	-2.666450000	-0.572447000
H	6.595005000	-1.248453000	-1.491719000
H	6.595000000	1.248445000	-1.491725000
H	5.834657000	2.666444000	-0.572453000

47
3d

C	-2.218499000	-2.874093000	-0.663745000
C	-2.218500000	-2.874092000	0.663749000
C	-3.175284000	-1.740010000	1.068996000
C	-2.466757000	-0.428662000	0.700190000
C	-2.466756000	-0.428663000	-0.700190000
C	-3.175283000	-1.740012000	-1.068995000
C	-1.910239000	0.600540000	1.435736000
C	-1.373416000	1.654289000	0.695633000
C	-1.373416000	1.654289000	-0.695632000
C	-1.910238000	0.600540000	-1.435736000
C	-0.597817000	2.899361000	1.070055000
C	0.912362000	2.624032000	0.785909000
C	0.912362000	2.624032000	-0.785910000
C	-0.597817000	2.899360000	-1.070055000
C	1.445315000	1.382661000	1.526261000
C	1.448934000	0.167861000	0.666090000
C	1.448933000	0.167860000	-0.666089000
C	1.445315000	1.382660000	-1.526262000
C	1.760882000	-1.268744000	1.069849000
C	3.248323000	-1.446496000	0.744466000
C	3.248322000	-1.446496000	-0.744467000
C	1.760881000	-1.268744000	-1.069848000
C	4.259743000	-1.569478000	-1.595786000
C	4.259744000	-1.569478000	1.595784000
O	-4.130890000	-1.808166000	-0.000004000
O	-0.932980000	3.788550000	0.000000000
O	1.163124000	-2.008845000	0.000001000
H	-1.611212000	-3.453713000	-1.343016000
H	-1.611215000	-3.453711000	1.343022000
H	-3.655678000	-1.794973000	2.041078000
H	-3.655676000	-1.794976000	-2.041077000
H	-1.905789000	0.599129000	2.520773000
H	-1.905786000	0.599129000	-2.520773000
H	-0.808973000	3.350770000	2.036944000
H	1.460495000	3.502162000	1.128158000
H	1.460494000	3.502161000	-1.128159000
H	-0.808973000	3.350768000	-2.036945000
H	2.470045000	1.563785000	1.871917000
H	0.849379000	1.211671000	2.431382000
H	0.849378000	1.211670000	-2.431382000
H	2.470045000	1.563784000	-1.871917000
H	1.428194000	-1.622014000	2.043026000
H	1.428192000	-1.622014000	-2.043025000
H	4.093292000	-1.574754000	-2.667067000
H	5.281946000	-1.675773000	-1.249086000
H	5.281947000	-1.675773000	1.249083000
H	4.093294000	-1.574755000	2.667066000

47
TS-**3a**

C	5.150372000	1.964945000	-0.664019000
C	5.150372000	1.964946000	0.664017000
C	5.177889000	0.478347000	1.068302000
C	3.803561000	-0.096100000	0.697433000
C	3.803561000	-0.096101000	-0.697433000
C	5.177889000	0.478346000	-1.068302000
C	2.714110000	-0.535358000	1.436662000
C	1.628063000	-0.986523000	0.697780000
C	1.628064000	-0.986523000	-0.697779000
C	2.714110000	-0.535360000	-1.436662000

C	0.245994000	-1.529369000	1.071521000
C	-0.776663000	-0.445212000	0.689226000
C	-0.776663000	-0.445213000	-0.689227000
C	0.245994000	-1.529370000	-1.071520000
C	-2.684116000	-1.453216000	1.473076000
C	-3.631935000	-0.839745000	0.710141000
C	-3.631935000	-0.839745000	-0.710142000
C	-2.684116000	-1.453217000	-1.473076000
C	-4.532935000	0.336600000	1.075111000
C	-3.730853000	1.593330000	0.743369000
C	-3.730853000	1.593330000	-0.743369000
C	-4.532935000	0.336600000	-1.075111000
C	-3.177733000	2.452257000	-1.592597000
C	-3.177733000	2.452257000	1.592597000
O	5.966566000	-0.065411000	0.000000000
O	0.039878000	-2.463038000	0.000001000
O	-5.480889000	0.316065000	0.000000000
H	5.069483000	2.796332000	-1.348663000
H	5.069483000	2.796333000	1.348661000
H	5.585890000	0.221550000	2.041005000
H	5.585890000	0.221548000	-2.041005000
H	2.716890000	-0.541219000	2.521210000
H	2.716891000	-0.541221000	-2.521210000
H	0.141411000	-2.011025000	2.040434000
H	-0.980963000	0.417710000	1.306914000
H	-0.980963000	0.417709000	-1.306915000
H	0.141411000	-2.011026000	-2.040433000
H	-2.627369000	-1.271854000	2.541894000
H	-2.203209000	-2.353449000	1.109117000
H	-2.627369000	-1.271854000	-2.541894000
H	-2.203209000	-2.353449000	-1.109117000
H	-5.024750000	0.316315000	2.043461000
H	-5.024749000	0.316314000	-2.043461000
H	-3.279458000	2.324536000	-2.664481000
H	-2.614679000	3.310926000	-1.243151000
H	-2.614679000	3.310927000	1.243151000
H	-3.279459000	2.324536000	2.664481000

47
TS-**3b**

C	-3.249307000	-2.888643000	-0.664085000
C	-3.249305000	-2.888639000	0.664101000
C	-4.057179000	-1.641268000	1.068681000
C	-3.189366000	-0.430446000	0.699470000
C	-3.189368000	-0.430450000	-0.699467000
C	-4.057181000	-1.641273000	-1.068669000
C	-2.493615000	0.511760000	1.436642000
C	-1.806125000	1.471755000	0.696194000
C	-1.806125000	1.471751000	-0.696204000
C	-2.493618000	0.511752000	-1.436645000
C	-0.920539000	2.654158000	1.069327000
C	0.544919000	2.324033000	0.692194000
C	0.544918000	2.324024000	-0.692204000
C	-0.920538000	2.654149000	-1.069343000
C	1.051905000	0.246613000	1.473929000
C	2.150294000	-0.024099000	0.707029000
C	2.150278000	-0.024117000	-0.707014000
C	1.051870000	0.246573000	-1.473892000
C	3.626197000	0.040728000	1.075706000
C	4.224134000	-1.324277000	0.743732000
C	4.224119000	-1.324294000	-0.743735000
C	3.626172000	0.040702000	-1.075727000
C	4.651742000	-2.251245000	-1.592347000
C	4.651768000	-2.251211000	1.592357000
O	-5.013030000	-1.591957000	0.000007000
O	-1.265699000	3.562604000	-0.000012000
O	4.159080000	0.838048000	-0.000027000
H	-2.739843000	-3.551095000	-1.348235000
H	-2.739839000	-3.551088000	1.348254000
H	-4.539711000	-1.637762000	2.041041000
H	-4.539716000	-1.637773000	-2.041028000
H	-2.492739000	0.512610000	2.521414000
H	-2.492743000	0.512596000	-2.521417000
H	-1.077728000	3.109003000	2.042937000
H	1.365088000	2.673710000	1.299277000
H	1.365094000	2.673681000	-1.299289000

H	-1.077723000	3.108988000	-2.042957000
H	1.149071000	0.392030000	2.545506000
H	0.069077000	-0.037608000	1.119962000
H	0.069051000	-0.037634000	-1.119890000
H	1.149005000	0.391961000	-2.545477000
H	3.880376000	0.457424000	2.046699000
H	3.880327000	0.457375000	-2.046737000
H	4.599139000	-2.097636000	-2.664074000
H	5.067753000	-3.189490000	-1.242379000
H	5.067768000	-3.189466000	1.242402000
H	4.599187000	-2.097578000	2.664081000

47
TS-**3c**

C	-5.701529000	-0.664019000	1.913485000
C	-5.701528000	0.663989000	1.913498000
C	-5.636805000	1.068332000	0.428222000
C	-4.229243000	0.697496000	-0.059265000
C	-4.229245000	-0.697491000	-0.059280000
C	-5.636808000	-1.068333000	0.428201000
C	-3.113602000	1.436696000	-0.426634000
C	-2.000232000	0.697748000	-0.805448000
C	-2.000235000	-0.697735000	-0.805463000
C	-3.113607000	-1.436688000	-0.426664000
C	-0.584351000	1.071606000	-1.251490000
C	0.361063000	0.691042000	-0.098830000
C	0.361057000	-0.691053000	-0.098844000
C	-0.584356000	-1.071588000	-1.251513000
C	2.314264000	1.473917000	-0.953823000
C	3.212654000	0.708012000	-0.269257000
C	3.212657000	-0.708022000	-0.269254000
C	2.314272000	-1.473937000	-0.953817000
C	4.084140000	1.076028000	0.923469000
C	5.523228000	0.743787000	0.537311000
C	5.523232000	-0.743784000	0.537314000
C	4.084145000	-1.076030000	0.923472000
C	6.514438000	-1.591669000	0.290707000
C	6.514430000	1.591675000	0.290700000
O	-6.389909000	0.000007000	-0.163818000
O	-0.311630000	0.000018000	-2.167377000
O	3.794722000	-0.000001000	1.837856000
H	-5.671811000	-1.348563000	2.748326000
H	-5.671807000	1.348517000	2.748353000
H	-6.027988000	2.041033000	0.146488000
H	-6.027994000	-2.041027000	0.146447000
H	-3.115600000	2.521227000	-0.431693000
H	-3.115609000	-2.521217000	-0.431748000
H	-0.446358000	2.040204000	-1.725450000
H	0.478508000	1.305474000	0.781476000
H	0.478488000	-1.305508000	0.781448000
H	-0.446364000	-2.040176000	-1.725494000
H	2.256810000	2.544110000	-0.781138000
H	1.903241000	1.114851000	-1.890055000
H	1.903248000	-1.114878000	-1.890051000
H	2.256823000	-2.544128000	-0.781122000
H	3.917833000	2.047029000	1.382474000
H	3.917843000	-2.047031000	1.382479000
H	6.362155000	-2.663519000	0.344355000
H	7.506380000	-1.240910000	0.028253000
H	7.506373000	1.240920000	0.028245000
H	6.362142000	2.663525000	0.344344000

47
TS-**3d**

C	-4.118675000	-2.216491000	-0.664093000
C	-4.118675000	-2.216489000	0.664098000
C	-4.492179000	-0.777975000	1.068656000
C	-3.286804000	0.097409000	0.699365000
C	-3.286804000	0.097406000	-0.699370000
C	-4.492179000	-0.777979000	-1.068657000
C	-2.328124000	0.770671000	1.436624000
C	-1.371024000	1.461841000	0.696268000
C	-1.371023000	1.461837000	-0.696278000
C	-2.328123000	0.770664000	-1.436632000
C	-0.154449000	2.300548000	1.069333000

C	1.129040000	1.520824000	0.690607000
C	1.129040000	1.520815000	-0.690606000
C	-0.154446000	2.300537000	-1.069346000
C	0.940741000	-0.629469000	1.473190000
C	1.885555000	-1.251766000	0.708966000
C	1.885544000	-1.251783000	-0.708928000
C	0.940717000	-0.629502000	-1.473149000
C	3.322716000	-1.611522000	1.075037000
C	4.155365000	-0.374769000	0.743415000
C	4.155358000	-0.374792000	-0.743439000
C	3.322699000	-1.611551000	-1.075015000
C	4.720702000	0.476246000	-1.592588000
C	4.720705000	0.476300000	1.592535000
O	-5.383842000	-0.430049000	-0.000001000
O	-0.192818000	3.271330000	-0.000011000
H	-3.844190000	-3.005775000	-1.348320000
H	-3.844190000	-3.005769000	1.348328000
H	-4.948999000	-0.622466000	2.041036000
H	-4.948999000	-0.622475000	-2.041038000
H	-2.327068000	0.771099000	2.521408000
H	-2.327065000	0.771087000	-2.521416000
H	-0.158712000	2.781760000	2.042961000
H	2.018375000	1.565733000	1.301178000
H	2.018375000	1.565706000	-1.301178000
H	-0.158702000	2.781741000	-2.042978000
H	1.081415000	-0.511753000	2.543346000
H	-0.080264000	-0.577164000	1.117164000
H	-0.080280000	-0.577183000	-1.117100000
H	1.081368000	-0.511811000	-2.543311000
H	3.499067000	-2.070808000	2.043496000
H	3.499031000	-2.070865000	-2.043463000
H	4.644547000	0.332368000	-2.664546000
H	5.282112000	1.335836000	-1.242841000
H	5.282103000	1.335887000	1.242761000
H	4.644556000	0.332449000	2.664497000
O	3.680844000	-2.489239000	0.000020000

47
TS2-**3a**

C	1.741967000	0.643407000	-0.836103000
C	3.046838000	1.037902000	-1.104551000
C	4.038404000	0.251938000	-0.536414000
C	3.744510000	-0.854476000	0.263126000
C	2.442353000	-1.240787000	0.542204000
C	1.447639000	-0.465086000	-0.041332000
C	5.572320000	0.305346000	-0.545739000
C	5.999694000	0.709260000	0.878212000
C	5.720265000	-0.343682000	1.637842000
C	5.122620000	-1.388763000	0.676766000
C	-0.084253000	-0.508285000	0.000108000
C	-0.486330000	0.701872000	0.839256000
C	-0.257366000	1.784899000	0.057667000
C	0.367132000	1.196765000	-1.226817000
O	5.865575000	-1.098550000	-0.516260000
O	-0.378573000	-0.019783000	-1.325425000
C	-4.353205000	2.087543000	1.657621000
C	-3.920477000	1.058569000	0.931474000
C	-3.289043000	1.184783000	-0.403333000
C	-2.470842000	2.246992000	-0.742499000
C	-3.873519000	-0.400686000	1.381938000
C	-4.696892000	-1.185699000	0.368438000
C	-4.092001000	-1.097608000	-0.983293000
C	-3.345485000	0.101487000	-1.269645000
C	-5.802246000	-1.875404000	0.638948000
C	-4.136432000	-2.069432000	-1.915524000
H	3.275406000	1.895394000	-1.727959000
H	2.211566000	-2.101156000	1.160252000
H	6.054159000	0.800012000	-1.383414000
H	6.370478000	1.681919000	1.166121000
H	5.802324000	-0.456783000	2.708756000
H	5.194943000	-2.436314000	0.951795000
H	-0.601840000	-1.433419000	0.223361000
H	-0.785045000	0.642043000	1.872556000
H	-0.165688000	2.811719000	0.381673000
H	0.306787000	1.782771000	-2.140611000
H	-4.314454000	3.112428000	1.302770000

H	-4.776729000	1.921549000	2.642180000
H	-2.065950000	2.323270000	-1.745971000
H	-2.435262000	3.132971000	-0.121276000
H	-4.358711000	-0.477870000	2.372960000
H	-2.809132000	0.155080000	-2.211651000
H	-6.188600000	-1.920056000	1.651476000
H	-6.354862000	-2.406247000	-0.129793000
H	-4.626437000	-3.015826000	-1.718829000
H	-3.670015000	-1.933991000	-2.884914000
O	-2.571609000	-0.801994000	1.344235000

47
TS2-**3b**

C	-3.837134000	-1.344052000	-0.664288000
C	-3.837102000	-1.344122000	0.664164000
C	-3.666044000	0.130408000	1.069163000
C	-2.239157000	0.568115000	0.698812000
C	-2.239183000	0.568187000	-0.698805000
C	-3.666088000	0.130520000	-1.069144000
C	-1.175306000	1.064481000	1.434922000
C	-0.122601000	1.597292000	0.697308000
C	-0.122621000	1.597355000	-0.697265000
C	-1.175353000	1.064623000	-1.434899000
C	0.969834000	2.590125000	1.070831000
C	2.383931000	2.110832000	0.694020000
C	2.383916000	2.110873000	-0.693974000
C	0.969814000	2.590209000	-1.070724000
C	-0.476395000	-2.163561000	-1.591765000
C	0.541930000	-2.133500000	-0.742496000
C	0.542033000	-2.133568000	0.742564000
C	-0.476167000	-2.163681000	1.591982000
C	2.021680000	-2.221602000	-1.077043000
C	2.735246000	-0.920884000	-0.705660000
C	2.735328000	-0.920942000	0.705512000
C	2.021831000	-2.221701000	1.076883000
C	3.204932000	0.105067000	1.482042000
C	3.204793000	0.105175000	-1.482163000
O	-4.386111000	0.767647000	0.000058000
O	0.758931000	3.554047000	0.000093000
H	-3.895392000	-2.179678000	-1.346801000
H	-3.895321000	-2.179821000	1.346590000
H	-4.040442000	0.434974000	2.041749000
H	-4.040526000	0.435188000	-2.041682000
H	-1.201541000	1.120637000	2.517947000
H	-1.201622000	1.120891000	-2.517918000
H	0.876784000	3.062040000	2.044896000
H	3.208379000	2.484641000	1.280987000
H	3.208354000	2.484725000	-1.280928000
H	0.876755000	3.062203000	-2.044751000
H	-0.314272000	-2.204989000	-2.663300000
H	-1.498141000	-2.140317000	-1.233758000
H	-1.497973000	-2.140383000	1.234139000
H	-0.313882000	-2.205218000	2.663488000
H	2.268908000	-2.649230000	-2.044603000
H	2.269210000	-2.649414000	2.044367000
H	3.057881000	0.103225000	2.557186000
H	4.079548000	0.633193000	1.130178000
H	4.079489000	0.633222000	-1.130366000
H	3.057621000	0.103418000	-2.557290000
O	2.480114000	-3.062095000	-0.000150000

47
TS2-**3c**

C	-1.278649000	0.037400000	-0.698363000
C	-2.448822000	0.166163000	-1.436360000
C	-3.614849000	0.309468000	-0.697534000
C	-3.614853000	0.309468000	0.697537000
C	-2.448830000	0.166165000	1.436369000
C	-1.278653000	0.037401000	0.698379000
C	-5.096102000	0.466027000	-1.068335000
C	-5.785533000	-0.851304000	-0.664055000
C	-5.785537000	-0.851305000	0.664044000
C	-5.096109000	0.466027000	1.068328000
C	0.194631000	-0.166098000	1.070979000
C	0.536549000	-1.615923000	0.691555000

C	0.536547000	-1.615925000	-0.691530000
C	0.194634000	-0.166100000	-1.070956000
O	-5.527333000	1.321584000	-0.000005000
O	0.827960000	0.534309000	0.000012000
C	2.943451000	2.244997000	1.595282000
C	3.463788000	1.371352000	0.742686000
C	3.463764000	1.371347000	-0.742700000
C	2.943384000	2.244979000	-1.595284000
C	4.271133000	0.122958000	1.076160000
C	3.449237000	-1.113820000	0.707784000
C	3.449224000	-1.113825000	-0.707790000
C	4.271105000	0.122956000	-1.076192000
C	2.663334000	-1.927768000	1.473304000
C	2.663314000	-1.927785000	-1.473291000
H	-2.449136000	0.170453000	-2.521033000
H	-2.449150000	0.170457000	2.521043000
H	-5.330805000	0.887208000	-2.041032000
H	-6.112925000	-1.619757000	-1.348751000
H	-6.112935000	-1.619757000	1.348736000
H	-5.330817000	0.887209000	2.041023000
H	0.531114000	0.205492000	2.035421000
H	0.228989000	-2.452457000	1.302843000
H	0.228964000	-2.452457000	-1.302810000
H	0.531122000	0.205489000	-2.035396000
H	2.382854000	3.105779000	1.248198000
H	3.057937000	2.119460000	2.666343000
H	3.057832000	2.119437000	-2.666348000
H	2.382782000	3.105751000	-1.248183000
H	4.764783000	0.113117000	2.044031000
H	4.764730000	0.113112000	-2.044075000
H	2.587629000	-1.783569000	2.546461000
H	2.446085000	-2.925759000	1.115597000
H	2.446099000	-2.925778000	-1.115571000
H	2.587595000	-1.783598000	-2.546449000
O	5.225660000	0.123986000	-0.000027000

47

TS2-**3d**

C	3.095324000	1.871507000	-0.663559000
C	3.095283000	1.871559000	0.663457000
C	3.443242000	0.428738000	1.068864000
C	2.230307000	-0.436153000	0.699076000
C	2.230345000	-0.436206000	-0.699039000
C	3.443304000	0.428653000	-1.068830000
C	1.293648000	-1.138078000	1.435781000
C	0.368929000	-1.872874000	0.695911000
C	0.368975000	-1.872937000	-0.695865000
C	1.293728000	-1.138191000	-1.435741000
C	-0.620788000	-2.968700000	1.070195000
C	-2.060808000	-2.571034000	0.691577000
C	-2.060749000	-2.571112000	-0.691659000
C	-0.620704000	-2.968810000	-1.070115000
C	-2.693265000	-0.512382000	1.488075000
C	-2.016693000	0.381794000	0.706749000
C	-2.016638000	0.381838000	-0.706885000
C	-2.693147000	-0.512268000	-1.488334000
C	-0.971252000	1.425290000	1.074292000
C	-1.611779000	2.776935000	0.744965000
C	-1.611730000	2.776983000	-0.744909000
C	-0.971172000	1.425364000	-1.074281000
C	-2.027349000	3.710049000	-1.592768000
C	-2.027439000	3.709955000	1.592855000
O	4.331779000	0.066897000	0.000057000
O	-0.337993000	-3.907566000	0.000101000
O	-0.032814000	1.317261000	0.000036000
H	2.813710000	2.661542000	-1.344027000
H	2.813622000	2.661646000	1.343845000
H	3.898717000	0.265302000	2.041053000
H	3.898832000	0.265136000	-2.040979000
H	1.314727000	-1.168177000	2.520373000
H	1.314871000	-1.168378000	-2.520330000
H	-0.493594000	-3.431918000	2.044574000
H	-2.888574000	-2.912069000	1.294511000
H	-2.888490000	-2.912109000	-1.294646000
H	-0.493426000	-3.432135000	-2.044432000
H	-3.645728000	-0.893969000	1.145818000

H	-2.530046000	-0.544252000	2.561000000
H	-2.529823000	-0.544079000	-2.561244000
H	-3.645610000	-0.893944000	-1.146177000
H	-0.486564000	1.336859000	2.043624000
H	-0.486412000	1.336999000	-2.043583000
H	-1.948439000	3.568522000	-2.664649000
H	-2.457163000	4.642240000	-1.242796000
H	-2.457216000	4.642173000	1.242914000
H	-1.948598000	3.568360000	2.664732000

50

4a

C	-5.131683000	2.216397000	0.664091000
C	-5.131683000	2.216397000	-0.664091000
C	-5.319388000	0.742290000	-1.068748000
C	-4.013620000	0.025296000	-0.699790000
C	-4.013620000	0.025296000	0.699790000
C	-5.319388000	0.742290000	1.068748000
C	-2.980802000	-0.525564000	-1.436105000
C	-1.951528000	-1.106678000	-0.696048000
C	-1.951528000	-1.106678000	0.696048000
C	-2.980802000	-0.525564000	1.436105000
C	-0.635985000	-1.758266000	-1.074004000
C	0.500368000	-0.739633000	-0.782596000
C	0.500368000	-0.739633000	0.782596000
C	-0.635985000	-1.758266000	1.074004000
C	1.838880000	-1.174460000	-1.411436000
C	2.994230000	-0.575157000	-0.666111000
C	2.994230000	-0.575157000	0.666111000
C	1.838880000	-1.174460000	1.411436000
C	4.187588000	0.136951000	-1.281543000
C	4.089721000	1.573641000	-0.744209000
C	4.089721000	1.573641000	0.744209000
C	4.187588000	0.136951000	1.281543000
C	4.010968000	2.642879000	1.533314000
C	4.010968000	2.642879000	-1.533314000
O	-6.160445000	0.284579000	0.000000000
O	-0.458345000	-2.694770000	0.000000000
C	5.461990000	-0.423207000	0.665018000
C	5.461990000	-0.423208000	-0.665018000
H	-4.958984000	3.034088000	1.348243000
H	-4.958984000	3.034088000	-1.348243000
H	-5.753184000	0.530727000	-2.041144000
H	-5.753184000	0.530726000	2.041144000
H	-2.978720000	-0.522758000	-2.520730000
H	-2.978720000	-0.522758000	2.520730000
H	-0.583704000	-2.262109000	-2.037115000
H	0.243018000	0.250986000	-1.160582000
H	0.243018000	0.250986000	1.160582000
H	-0.583704000	-2.262109000	2.037115000
H	1.856936000	-0.897660000	-2.468625000
H	1.908012000	-2.268675000	-1.362396000
H	1.908012000	-2.268675000	1.362396000
H	1.856936000	-0.897660000	2.468625000
H	4.187172000	0.119442000	-2.370095000
H	4.187172000	0.119442000	2.370095000
H	4.023085000	2.542202000	2.612380000
H	3.934688000	3.646099000	1.129179000
H	3.934689000	3.646099000	-1.129179000
H	4.023086000	2.542202000	-2.612380000
H	6.292406000	-0.738498000	1.283883000
H	6.292406000	-0.738498000	-1.283883000

50

4b

C	3.372316000	-1.734190000	0.664323000
C	3.372191000	-1.734337000	-0.664073000
C	3.590694000	-0.267352000	-1.068864000
C	2.319487000	0.510728000	-0.699366000
C	2.319581000	0.510864000	0.699263000
C	3.590857000	-0.267119000	1.068754000
C	1.379268000	1.209377000	-1.435981000
C	0.463775000	1.956320000	-0.696500000
C	0.463860000	1.956442000	0.696358000
C	1.379457000	1.209651000	1.435864000

C	-0.645969000	2.919136000	-1.070169000
C	-2.016441000	2.238558000	-0.785601000
C	-2.016343000	2.238612000	0.785639000
C	-0.645867000	2.919292000	1.069986000
C	-2.195272000	0.908526000	-1.533390000
C	-2.122818000	-0.303455000	-0.664554000
C	-2.122648000	-0.303416000	0.664738000
C	-2.194970000	0.908606000	1.533530000
C	-2.097086000	-1.698819000	-1.274626000
C	-0.816893000	-2.352027000	-0.742962000
C	-0.816634000	-2.351908000	0.742856000
C	-2.096709000	-1.698750000	1.274873000
C	0.128725000	-2.859636000	1.530597000
C	0.128083000	-2.860067000	-1.530964000
O	4.448674000	0.167250000	-0.000149000
O	-0.570241000	3.869390000	-0.000174000
C	-3.229853000	-2.513359000	0.665538000
C	-3.230054000	-2.513390000	-0.664907000
H	3.210707000	-2.549629000	1.352843000
H	3.210430000	-2.549925000	-1.352383000
H	4.030032000	-0.068994000	-2.041717000
H	4.030329000	-0.068529000	2.041499000
H	1.392115000	1.226515000	-2.520660000
H	1.392441000	1.226999000	2.520539000
H	-0.566643000	3.411741000	-2.036815000
H	-2.784354000	2.937150000	-1.118651000
H	-2.784269000	2.937163000	1.118743000
H	-0.566472000	3.412039000	2.036553000
H	-3.159531000	0.898272000	-2.054668000
H	-1.436032000	0.824181000	-2.322618000
H	-1.435582000	0.824328000	2.322620000
H	-3.159133000	0.898333000	2.054987000
H	-2.112176000	-1.683504000	-2.363761000
H	-2.111469000	-1.683373000	2.364012000
H	0.038657000	-2.822477000	2.610247000
H	1.010684000	-3.333740000	1.117543000
H	1.010087000	-3.334310000	-1.118164000
H	0.037622000	-2.823069000	-2.610587000
H	-3.919474000	-3.070974000	1.286464000
H	-3.919858000	-3.071037000	-1.285601000

50

4c

C	-5.659237000	1.908842000	0.663990000
C	-5.659236000	1.908768000	-0.664204000
C	-5.638401000	0.422906000	-1.068801000
C	-4.245162000	-0.104576000	-0.699837000
C	-4.245163000	-0.104497000	0.699850000
C	-5.638402000	0.423025000	1.068754000
C	-3.146528000	-0.508290000	-1.436106000
C	-2.047303000	-0.943087000	-0.695951000
C	-2.047303000	-0.943007000	0.696059000
C	-3.146528000	-0.508126000	1.436165000
C	-0.655687000	-1.410327000	-1.073814000
C	0.332309000	-0.246277000	-0.783153000
C	0.332307000	-0.246183000	0.783176000
C	-0.655686000	-1.410200000	1.073975000
C	1.712723000	-0.493163000	-1.418019000
C	2.785703000	0.237186000	-0.666270000
C	2.785701000	0.237271000	0.666239000
C	1.712720000	-0.492985000	1.418077000
C	4.024840000	0.867284000	-1.281135000
C	5.188397000	0.018276000	-0.744070000
C	5.188397000	0.018376000	0.744077000
C	4.024835000	0.867449000	1.281027000
C	6.047761000	-0.622016000	1.533496000
C	6.047753000	-0.622234000	-1.533402000
O	-6.406968000	-0.148140000	0.000008000
O	-0.352477000	-2.313348000	0.000134000
C	4.229135000	2.243630000	0.664868000
C	4.229138000	2.243544000	-0.665152000
H	-5.604175000	2.742728000	1.348192000
H	-5.604174000	2.742577000	-1.348499000
H	-6.038285000	0.152507000	-2.041127000
H	-6.038286000	0.152735000	2.041109000
H	-3.145199000	-0.505934000	-2.520740000

H	-3.145199000	-0.505647000	2.520799000
H	-0.535125000	-1.902306000	-2.036882000
H	-0.061360000	0.699383000	-1.158689000
H	-0.061367000	0.699521000	1.158597000
H	-0.535122000	-1.902066000	2.037102000
H	1.690776000	-0.201282000	-2.471284000
H	1.926943000	-1.569792000	-1.386594000
H	1.926943000	-1.569617000	1.386788000
H	1.690769000	-0.200972000	2.471305000
H	4.009501000	0.876021000	-2.369801000
H	4.009491000	0.876326000	2.369693000
H	5.966298000	-0.561296000	2.612524000
H	6.854281000	-1.223744000	1.129731000
H	6.854264000	-1.223919000	-1.129557000
H	5.966289000	-0.561659000	-2.612439000
H	4.402897000	3.114489000	1.284046000
H	4.402903000	3.114324000	-1.284442000

50
4d

C	2.555169000	-3.922830000	-0.664117000
C	2.555169000	-3.922830000	0.664117000
C	1.188188000	-4.505322000	1.068865000
C	0.142982000	-3.444030000	0.699997000
C	0.142982000	-3.444030000	-0.699997000
C	1.188188000	-4.505322000	-1.068865000
C	-0.668257000	-2.600445000	1.435388000
C	-1.507345000	-1.765810000	0.695988000
C	-1.507345000	-1.765810000	-0.695988000
C	-0.668257000	-2.600445000	-1.435388000
C	-2.498579000	-0.684652000	1.075737000
C	-1.855891000	0.702408000	0.783201000
C	-1.855891000	0.702408000	-0.783201000
C	-2.498579000	-0.684652000	-1.075737000
C	-0.507633000	0.994806000	1.458244000
C	0.280669000	1.998653000	0.666028000
C	0.280669000	1.998653000	-0.666028000
C	-0.507633000	0.994806000	-1.458244000
C	1.129785000	3.102510000	1.278527000
C	0.512406000	4.403424000	0.743913000
C	0.512406000	4.403424000	-0.743913000
C	1.129785000	3.102510000	-1.278527000
C	0.047969000	5.368967000	-1.533846000
C	0.047969000	5.368967000	1.533846000
O	0.976663000	-5.438953000	0.000000000
O	-3.445465000	-0.779997000	0.000000000
C	2.522100000	3.048933000	-0.665001000
C	2.522100000	3.048933000	0.665001000
H	3.294938000	-3.533817000	-1.348150000
H	3.294938000	-3.533817000	1.348150000
H	1.102539000	-4.980445000	2.041141000
H	1.102539000	-4.980445000	-2.041141000
H	-0.667170000	-2.597538000	2.520260000
H	-0.667170000	-2.597538000	-2.520260000
H	-2.996183000	-0.783308000	2.037606000
H	-2.566643000	1.461407000	1.114158000
H	-2.566643000	1.461407000	-1.114158000
H	-2.996183000	-0.783308000	-2.037606000
H	-0.673673000	1.354177000	2.477827000
H	0.083024000	0.074171000	1.540800000
H	0.083024000	0.074171000	-1.540800000
H	-0.673673000	1.354177000	-2.477827000
H	1.134660000	3.084608000	2.367154000
H	1.134660000	3.084608000	-2.367154000
H	0.095422000	5.278848000	-2.612847000
H	-0.390680000	6.274607000	-1.130175000
H	-0.390680000	6.274607000	1.130175000
H	0.095422000	5.278848000	2.612847000
H	3.409596000	3.063407000	-1.284844000
H	3.409596000	3.063407000	1.284844000

50
TS-**4a**

C	1.999145000	-5.347322000	-0.664023000
C	1.999145000	-5.347322000	0.664023000

C	0.512893000	-5.391823000	1.068280000
C	-0.077099000	-4.024158000	0.697331000
C	-0.077099000	-4.024158000	-0.697331000
C	0.512893000	-5.391823000	-1.068280000
C	-0.527085000	-2.938834000	1.436489000
C	-0.988526000	-1.856969000	0.697956000
C	-0.988526000	-1.856969000	-0.697956000
C	-0.527085000	-2.938834000	-1.436489000
C	-1.542394000	-0.479043000	1.071635000
C	-0.467437000	0.552643000	0.689061000
C	-0.467437000	0.552643000	-0.689061000
C	-1.542394000	-0.479043000	-1.071635000
C	-1.501583000	2.457497000	1.439407000
C	-0.845895000	3.409198000	0.710398000
C	-0.845895000	3.409198000	-0.710398000
C	-1.501583000	2.457497000	-1.439407000
C	0.277473000	4.268968000	1.284431000
C	1.563335000	3.636778000	0.743887000
C	1.563335000	3.636778000	-0.743887000
C	0.277473000	4.268968000	-1.284431000
C	2.513985000	3.136629000	-1.531551000
C	2.513985000	3.136629000	1.531551000
O	-0.021490000	-6.187086000	0.000000000
O	-2.478329000	-0.282187000	0.000000000
C	0.181207000	5.649505000	-0.665534000
C	0.181207000	5.649505000	0.665534000
H	2.829506000	-5.255833000	-1.348586000
H	2.829506000	-5.255833000	1.348586000
H	0.260875000	-5.802822000	2.041000000
H	0.260875000	-5.802822000	-2.041000000
H	-0.532359000	-2.941473000	2.521073000
H	-0.532359000	-2.941473000	-2.521073000
H	-2.025508000	-0.378304000	2.040301000
H	0.392052000	0.764918000	1.308674000
H	0.392052000	0.764918000	-1.308674000
H	-2.025508000	-0.378304000	-2.040301000
H	-1.358895000	2.401664000	2.514357000
H	-2.388635000	1.975066000	1.048129000
H	-2.388635000	1.975066000	-1.048129000
H	-1.358895000	2.401664000	-2.514357000
H	0.269210000	4.280192000	2.372898000
H	0.269210000	4.280192000	-2.372898000
H	2.421660000	3.177542000	-2.610847000
H	3.409675000	2.678608000	-1.127089000
H	3.409675000	2.678608000	1.127089000
H	2.421660000	3.177542000	2.610847000
H	0.133583000	6.539122000	-1.280779000
H	0.133583000	6.539122000	1.280779000

50
TS-**4b**

C	-3.546552000	-2.813344000	-0.664109000
C	-3.546557000	-2.813347000	0.664083000
C	-4.297593000	-1.530933000	1.068697000
C	-3.376404000	-0.360319000	0.699433000
C	-3.376399000	-0.360315000	-0.699447000
C	-4.297584000	-1.530928000	-1.068723000
C	-2.636476000	0.547780000	1.436592000
C	-1.904687000	1.474220000	0.696259000
C	-1.904682000	1.474225000	-0.696252000
C	-2.636465000	0.547788000	-1.436596000
C	-0.963838000	2.613283000	1.069360000
C	0.483747000	2.214911000	0.691161000
C	0.483752000	2.214923000	-0.691139000
C	-0.963833000	2.613294000	-1.069339000
C	0.881442000	0.086276000	1.436776000
C	2.004178000	-0.203009000	0.708081000
C	2.004196000	-0.202984000	-0.708093000
C	0.881476000	0.086321000	-1.436805000
C	3.414260000	-0.180916000	1.284391000
C	4.114400000	-1.428779000	0.743770000
C	4.114440000	-1.428736000	-0.743781000
C	3.414292000	-0.180862000	-1.284366000
C	4.638963000	-2.364876000	-1.531519000
C	4.638821000	-2.364999000	1.531481000
O	-5.250554000	-1.439109000	-0.000017000

O	-1.267762000	3.537381000	0.000014000
C	4.141894000	1.005776000	-0.665666000
C	4.141879000	1.005747000	0.665759000
H	-3.066339000	-3.497392000	-1.348171000
H	-3.066350000	-3.497398000	1.348146000
H	-4.779573000	-1.505995000	2.041049000
H	-4.779558000	-1.505986000	-2.041078000
H	-2.634141000	0.547468000	2.521359000
H	-2.634123000	0.547481000	-2.521363000
H	-1.100024000	3.074470000	2.043219000
H	1.320586000	2.513521000	1.303065000
H	1.320592000	2.513553000	-1.303031000
H	-1.100017000	3.074490000	-2.043195000
H	0.946045000	0.197871000	2.515051000
H	-0.100191000	-0.143975000	1.045262000
H	-0.100166000	-0.143951000	-1.045324000
H	0.946106000	0.197954000	-2.515075000
H	3.413087000	-0.166937000	2.372852000
H	3.413146000	-0.166837000	-2.372827000
H	4.586024000	-2.279221000	-2.610570000
H	5.133039000	-3.240928000	-1.127074000
H	5.132860000	-3.241062000	1.127012000
H	4.585827000	-2.279405000	2.610534000
H	4.634852000	1.747490000	-1.281757000
H	4.634822000	1.747435000	1.281894000

50
TS-**4c**

C	-5.738545000	2.009968000	0.530576000
C	-5.739178000	1.920497000	-0.794486000
C	-5.718224000	0.409970000	-1.097682000
C	-4.325499000	-0.092900000	-0.692655000
C	-4.324943000	0.001072000	0.698896000
C	-5.717058000	0.553799000	1.034071000
C	-3.221545000	-0.543256000	-1.403449000
C	-2.119945000	-0.905419000	-0.639060000
C	-2.119566000	-0.811274000	0.753628000
C	-3.220616000	-0.349590000	1.463060000
C	-0.718289000	-1.419835000	-0.979045000
C	0.262611000	-0.274277000	-0.674091000
C	0.262673000	-0.181047000	0.702543000
C	-0.717927000	-1.275060000	1.159277000
C	2.204920000	-1.268480000	-1.356137000
C	3.123230000	-0.517771000	-0.676171000
C	3.122763000	-0.421924000	0.739064000
C	2.204406000	-1.074104000	1.513829000
C	3.972614000	0.568087000	-1.325734000
C	5.387007000	0.392384000	-0.771272000
C	5.386289000	0.493435000	0.713001000
C	3.971325000	0.741653000	1.237157000
C	6.446954000	0.387724000	1.510537000
C	6.448305000	0.178283000	-1.545867000
O	-6.488354000	-0.085952000	0.006920000
O	-0.477114000	-2.270287000	0.152552000
C	3.478489000	1.998123000	0.531755000
C	3.479320000	1.908180000	-0.796681000
H	-5.683840000	2.887801000	1.157394000
H	-5.685049000	2.706130000	-1.533623000
H	-6.118163000	0.075086000	-2.049782000
H	-6.115949000	0.350024000	2.023005000
H	-3.224416000	-0.621811000	-2.485187000
H	-3.222730000	-0.282219000	2.545539000
H	-0.595497000	-1.962432000	-1.913070000
H	0.418180000	0.553022000	-1.350741000
H	0.418333000	0.729772000	1.261675000
H	-0.594444000	-1.686802000	2.157823000
H	2.153096000	-1.214253000	-2.439344000
H	1.761623000	-2.145264000	-0.900995000
H	1.761269000	-2.004354000	1.181247000
H	2.152509000	-0.873478000	2.579658000
H	3.961896000	0.502407000	-2.412264000
H	3.959151000	0.823103000	2.322627000
H	6.345408000	0.470565000	2.586365000
H	7.442441000	0.213548000	1.118373000
H	7.443112000	0.057127000	-1.132542000
H	6.347989000	0.114757000	-2.623119000

H	3.189945000	2.880514000	1.089418000
H	3.191438000	2.707243000	-1.468619000

50
TS-**4d**

C	-4.123826000	-2.341328000	-0.664115000
C	-4.123826000	-2.341331000	0.664111000
C	-4.581108000	-0.927105000	1.068666000
C	-3.429624000	0.017861000	0.699327000
C	-3.429625000	0.017864000	-0.699322000
C	-4.581109000	-0.927101000	-1.068664000
C	-2.511976000	0.746157000	1.436539000
C	-1.597533000	1.492809000	0.696387000
C	-1.597535000	1.492813000	-0.696379000
C	-2.511978000	0.746163000	-1.436533000
C	-0.430081000	2.398868000	1.069315000
C	0.894081000	1.691499000	0.690563000
C	0.894078000	1.691505000	-0.690562000
C	-0.430084000	2.398875000	-1.069305000
C	0.817704000	-0.477188000	1.437181000
C	1.843624000	-1.017575000	0.709209000
C	1.843625000	-1.017570000	-0.709221000
C	0.817703000	-0.477183000	-1.437191000
C	3.236090000	-1.263113000	1.284492000
C	4.083893000	-0.107717000	0.743842000
C	4.083885000	-0.107701000	-0.743837000
C	3.236093000	-1.263098000	-1.284504000
C	4.693053000	0.776694000	-1.531858000
C	4.693095000	0.776643000	1.531875000
O	-5.492107000	-0.632784000	0.000002000
O	-0.524868000	3.366468000	0.000008000
C	3.788239000	-2.531874000	-0.665514000
C	3.788236000	-2.531883000	0.665489000
H	-3.802704000	-3.112850000	-1.348328000
H	-3.802703000	-3.112854000	1.348320000
H	-5.046351000	-0.798871000	2.041046000
H	-5.046353000	-0.798863000	-2.041044000
H	-2.509989000	0.745330000	2.521327000
H	-2.509993000	0.745341000	-2.521322000
H	-0.461811000	2.879043000	2.042996000
H	1.778292000	1.784499000	1.302953000
H	1.778288000	1.784507000	-1.302953000
H	-0.461818000	2.879057000	-2.042983000
H	0.911618000	-0.368568000	2.513612000
H	-0.192102000	-0.487921000	1.049307000
H	-0.192103000	-0.487920000	-1.049317000
H	0.911617000	-0.368557000	-2.513621000
H	3.233706000	-1.276783000	2.372944000
H	3.233712000	-1.276754000	-2.372956000
H	4.629469000	0.697760000	-2.611108000
H	5.274250000	1.597980000	-1.127794000
H	5.274315000	1.597917000	1.127821000
H	4.629523000	0.697688000	2.611124000
H	4.158189000	-3.342276000	-1.280796000
H	4.158185000	-3.342292000	1.280762000

50
TS2-**4a**

C	-1.776153000	-0.698476000	-0.158106000
C	-2.949593000	-1.436288000	-0.252172000
C	-4.119449000	-0.697450000	-0.361421000
C	-4.119447000	0.697462000	-0.361400000
C	-2.949591000	1.436295000	-0.252130000
C	-1.776151000	0.698478000	-0.158086000
C	-5.604474000	-1.068273000	-0.476760000
C	-6.257380000	-0.664056000	0.859079000
C	-6.257379000	0.664034000	0.859099000
C	-5.604473000	1.068291000	-0.476727000
C	-0.297165000	1.070376000	0.001153000
C	0.090805000	0.690684000	1.439417000
C	0.090806000	-0.690729000	1.439396000
C	-0.297166000	-1.070381000	0.001122000
O	-6.059295000	0.000022000	-1.320118000
O	0.314160000	0.000008000	-0.721368000
C	2.242382000	1.441104000	1.675855000

C	2.955977000	0.708174000	0.765559000
C	2.955977000	-0.708199000	0.765539000
C	2.242376000	-1.441155000	1.675810000
C	3.687821000	1.284598000	-0.442142000
C	5.117297000	0.744701000	-0.311822000
C	5.117300000	-0.744687000	-0.311830000
C	3.687828000	-1.284588000	-0.442174000
C	6.186261000	1.531932000	-0.214401000
C	6.186264000	-1.531914000	-0.214386000
C	3.141160000	-0.664107000	-1.716084000
C	3.141157000	0.664147000	-1.716067000
H	-2.950166000	-2.520979000	-0.256414000
H	-2.950162000	2.520986000	-0.256341000
H	-5.850833000	-2.040945000	-0.891296000
H	-6.563116000	-1.348759000	1.636395000
H	-6.563114000	1.348714000	1.636437000
H	-5.850829000	2.040976000	-0.891234000
H	0.024768000	2.037509000	-0.376182000
H	-0.182076000	1.305750000	2.285187000
H	-0.182062000	-1.305822000	2.285152000
H	0.024765000	-2.037503000	-0.376240000
H	2.072909000	1.052353000	2.670853000
H	2.180345000	2.520757000	1.577991000
H	2.180337000	-2.520805000	1.577914000
H	2.072885000	-1.052430000	2.670815000
H	3.677090000	2.373361000	-0.447264000
H	3.677102000	-2.373351000	-0.447324000
H	6.085657000	2.611072000	-0.217314000
H	7.188523000	1.127423000	-0.129507000
H	7.188522000	-1.127402000	-0.129458000
H	6.085665000	-2.611055000	-0.217311000
H	2.792468000	-1.279691000	-2.535456000
H	2.792465000	1.279751000	-2.535424000

50
TS2-**4b**

C	3.768085000	-1.653438000	0.664191000
C	3.768089000	-1.653431000	-0.664204000
C	3.728799000	-0.170253000	-1.069127000
C	2.349046000	0.399416000	-0.698557000
C	2.349043000	0.399410000	0.698556000
C	3.728793000	-0.170264000	1.069129000
C	1.349412000	1.016200000	-1.434313000
C	0.367000000	1.670184000	-0.697488000
C	0.366997000	1.670178000	0.697489000
C	1.349405000	1.016188000	1.434312000
C	-0.587898000	2.797740000	-1.070622000
C	-2.050419000	2.504452000	-0.692831000
C	-2.050422000	2.504447000	0.692830000
C	-0.587902000	2.797730000	1.070630000
C	0.386995000	-2.194499000	1.528400000
C	-0.664354000	-1.980549000	0.742179000
C	-0.664338000	-1.980550000	-0.742156000
C	0.387025000	-2.194525000	-1.528353000
C	-2.074321000	-1.774351000	1.285147000
C	-2.656709000	-0.489937000	0.706736000
C	-2.656693000	-0.489932000	-0.706751000
C	-2.074290000	-1.774340000	-1.285157000
C	-3.083377000	0.584485000	-1.446666000
C	-3.083404000	0.584477000	1.446648000
O	4.504622000	0.399661000	0.000006000
O	-0.249613000	3.724938000	0.000009000
C	-2.888977000	-2.906016000	-0.665829000
C	-2.888993000	-2.906021000	0.665791000
H	3.751053000	-2.490311000	1.347296000
H	3.751061000	-2.490297000	-1.347318000
H	4.129120000	0.099365000	-2.041785000
H	4.129108000	0.099344000	2.041792000
H	1.384975000	1.072823000	-2.517082000
H	1.384963000	1.072802000	2.517082000
H	-0.433521000	3.253113000	-2.044895000
H	-2.827763000	2.957493000	-1.288401000
H	-2.827768000	2.957483000	1.288400000
H	-0.433530000	3.253094000	2.044907000
H	0.288932000	-2.183861000	2.608360000
H	1.371695000	-2.371606000	1.114657000

H	1.371711000	-2.371658000	-1.114590000
H	0.288984000	-2.183891000	-2.608315000
H	-2.089715000	-1.779716000	2.373496000
H	-2.089658000	-1.779698000	-2.373506000
H	-2.976241000	0.579377000	-2.526974000
H	-3.892724000	1.188021000	-1.062211000
H	-3.892737000	1.188023000	1.062179000
H	-2.976290000	0.579362000	2.526959000
H	-3.385624000	-3.643423000	-1.283737000
H	-3.385655000	-3.643433000	1.283681000

50
TS2-**4c**

C	-1.456882000	-0.012902000	0.698562000
C	-2.625285000	-0.158693000	1.436159000
C	-3.789273000	-0.318986000	0.697458000
C	-3.789272000	-0.318987000	-0.697454000
C	-2.625284000	-0.158692000	-1.436154000
C	-1.456882000	-0.012901000	-0.698556000
C	-5.268204000	-0.495930000	1.068290000
C	-5.975848000	0.811805000	0.664059000
C	-5.975847000	0.811803000	-0.664063000
C	-5.268202000	-0.495933000	-1.068288000
C	0.012942000	0.213964000	-1.070794000
C	0.334452000	1.668732000	-0.690732000
C	0.334452000	1.668729000	0.690747000
C	0.012942000	0.213959000	1.070802000
O	-5.688175000	-1.357203000	0.000002000
O	0.656494000	-0.477863000	0.000002000
C	2.805635000	-2.323348000	-1.533354000
C	3.292081000	-1.370139000	-0.742733000
C	3.292112000	-1.370148000	0.742725000
C	2.805720000	-2.323374000	1.533358000
C	3.964111000	-0.106972000	-1.284038000
C	3.227473000	1.099726000	-0.708546000
C	3.227491000	1.099720000	0.708552000
C	3.964146000	-0.106979000	1.284018000
C	2.472303000	1.976415000	-1.440810000
C	2.472336000	1.976403000	1.440840000
C	5.354710000	-0.097088000	0.665707000
C	5.354691000	-0.097086000	-0.665766000
H	-2.625662000	-0.162664000	2.520871000
H	-2.625660000	-0.162664000	-2.520866000
H	-5.497201000	-0.920300000	2.040991000
H	-6.313205000	1.576073000	1.348620000
H	-6.313202000	1.576068000	-1.348628000
H	-5.497198000	-0.920307000	-2.040988000
H	0.355778000	-0.152157000	-2.035260000
H	0.028059000	2.502486000	-1.306488000
H	0.028062000	2.502481000	1.306508000
H	0.355777000	-0.152166000	2.035266000
H	2.334004000	-3.212379000	-1.130726000
H	2.857731000	-2.233203000	-2.612597000
H	2.857854000	-2.233234000	2.612600000
H	2.334098000	-3.212416000	1.130744000
H	3.977050000	-0.098323000	-2.372740000
H	3.977116000	-0.098337000	2.372720000
H	2.402901000	1.868437000	-2.519015000
H	2.272906000	2.967486000	-1.055983000
H	2.272927000	2.967476000	1.056025000
H	2.402959000	1.868417000	2.519046000
H	6.244341000	-0.114428000	1.282623000
H	6.244305000	-0.114423000	-1.282706000

50
TS2-**4d**

C	2.591684000	2.882711000	-0.664121000
C	2.591684000	2.882712000	0.664121000
C	3.353708000	1.606472000	1.068755000
C	2.465346000	0.409788000	0.697954000
C	2.465346000	0.409788000	-0.697954000
C	3.353708000	1.606472000	-1.068755000
C	1.812805000	-0.564998000	1.435385000
C	1.192191000	-1.568583000	0.696849000
C	1.192192000	-1.568583000	-0.696849000

C	1.812806000	-0.564998000	-1.435385000
C	0.697445000	-2.960533000	1.070324000
C	-0.779695000	-3.171382000	0.691468000
C	-0.779695000	-3.171382000	-0.691468000
C	0.697445000	-2.960533000	-1.070324000
C	-3.874071000	2.410552000	1.532404000
C	-3.011513000	1.772087000	0.745103000
C	-3.011513000	1.772087000	-0.745103000
C	-3.874071000	2.410552000	-1.532404000
C	-1.855698000	0.917388000	1.285738000
C	-2.128607000	-0.464754000	0.707513000
C	-2.128607000	-0.464754000	-0.707514000
C	-1.855698000	0.917388000	-1.285738000
C	-2.304689000	-1.605431000	-1.447480000
C	-2.304689000	-1.605432000	1.447479000
O	4.310292000	1.532891000	0.000000000
O	1.332028000	-3.711883000	0.000000000
C	-0.608995000	1.513685000	-0.664468000
C	-0.608995000	1.513685000	0.664468000
H	2.120559000	3.575856000	-1.345906000
H	2.120559000	3.575856000	1.345906000
H	3.836349000	1.589879000	2.041181000
H	3.836350000	1.589879000	-2.041181000
H	1.848701000	-0.589185000	2.519489000
H	1.848702000	-0.589185000	-2.519489000
H	0.997687000	-3.335710000	2.044642000
H	-1.386586000	-3.827887000	1.295598000
H	-1.386585000	-3.827887000	-1.295598000
H	0.997688000	-3.335711000	-2.044641000
H	-3.797908000	2.344638000	2.611531000
H	-4.678288000	3.014724000	1.127876000
H	-4.678288000	3.014724000	-1.127876000
H	-3.797908000	2.344638000	-2.611531000
H	-1.843569000	0.912510000	2.374330000
H	-1.843570000	0.912510000	-2.374330000
H	-2.207371000	-1.574292000	-2.528396000
H	-2.931620000	-2.395074000	-1.059078000
H	-2.931620000	-2.395074000	1.059078000
H	-2.207371000	-1.574292000	2.528396000
H	0.173296000	1.938488000	-1.278286000
H	0.173296000	1.938488000	1.278286000

68

5

C	-2.644846000	-2.395154000	1.074358000
C	-1.177084000	-2.440543000	0.698251000
C	-1.177084000	-2.440543000	-0.698251000
C	-2.644846000	-2.395154000	-1.074358000
C	-3.169381000	-0.962303000	-0.783602000
C	-3.169381000	-0.962303000	0.783602000
C	0.000000000	-2.419042000	1.435308000
C	0.000000000	-2.419042000	-1.435308000
C	-4.553113000	-0.716031000	1.430158000
C	-4.553113000	-0.716031000	-1.430158000
O	-3.242507000	-3.137017000	0.000000000
C	1.177084000	-2.440543000	-0.698251000
C	1.177084000	-2.440543000	0.698251000
C	-5.289639000	0.334280000	0.666981000
C	2.644846000	-2.395154000	-1.074358000
C	-5.289639000	0.334280000	-0.666981000
C	3.169381000	-0.962303000	-0.783602000
C	3.169381000	-0.962303000	0.783602000
C	2.644846000	-2.395154000	1.074358000
O	3.242507000	-3.137017000	0.000000000
O	-6.743434000	1.973230000	0.000000000
C	-5.835282000	1.696863000	1.073588000
C	-4.698298000	2.672468000	0.744388000
C	-4.698298000	2.672468000	-0.744388000
C	-5.835282000	1.696863000	-1.073588000
C	-3.928055000	3.339540000	1.595576000
C	-3.928055000	3.339540000	-1.595576000
C	4.553113000	-0.716031000	-1.430158000
C	5.289639000	0.334280000	-0.666981000
C	5.289639000	0.334280000	0.666981000
C	4.553113000	-0.716031000	1.430158000
C	5.835282000	1.696863000	-1.073588000

C	4.698298000	2.672468000	-0.744388000
C	4.698298000	2.672468000	0.744388000
C	5.835282000	1.696863000	1.073588000
C	3.928055000	3.339540000	-1.595576000
C	3.928055000	3.339540000	1.595576000
O	6.743434000	1.973230000	0.000000000
H	-2.928616000	-2.815168000	2.037194000
H	-2.928616000	-2.815168000	-2.037194000
H	-2.475988000	-0.207662000	-1.158215000
H	-2.475988000	-0.207662000	1.158215000
H	0.000000000	-2.406691000	2.519952000
H	0.000000000	-2.406691000	-2.519952000
H	-4.429358000	-0.450737000	2.482665000
H	-5.127211000	-1.649344000	1.395929000
H	-5.127211000	-1.649344000	-1.395929000
H	-4.429358000	-0.450737000	-2.482665000
H	2.928616000	-2.815168000	-2.037194000
H	2.475988000	-0.207662000	-1.158215000
H	2.475988000	-0.207662000	1.158215000
H	2.928616000	-2.815168000	2.037194000
H	-6.309837000	1.812616000	2.044075000
H	-6.309837000	1.812616000	-2.044075000
H	-3.137969000	3.996657000	1.248615000
H	-4.072233000	3.254540000	2.666734000
H	-4.072233000	3.254540000	-2.666734000
H	-3.137969000	3.996657000	-1.248615000
H	5.127211000	-1.649344000	-1.395929000
H	4.429358000	-0.450737000	-2.482665000
H	4.429358000	-0.450737000	2.482665000
H	5.127211000	-1.649344000	1.395929000
H	6.309837000	1.812616000	-2.044075000
H	6.309837000	1.812616000	2.044075000
H	3.137969000	3.996657000	-1.248615000
H	4.072233000	3.254540000	-2.666734000
H	4.072233000	3.254540000	2.666734000
H	3.137969000	3.996657000	1.248615000

68
TS-**3a-5**

C	-2.620702000	-2.078393000	1.071993000
C	-1.138874000	-2.171654000	0.700197000
C	-1.138874000	-2.171654000	-0.700197000
C	-2.620702000	-2.078393000	-1.071993000
C	-3.087232000	-0.663406000	-0.689398000
C	-3.087232000	-0.663405000	0.689397000
C	0.031402000	-2.229115000	1.436092000
C	0.031403000	-2.229116000	-1.436092000
C	-5.240770000	-0.767721000	1.473238000
C	-5.240770000	-0.767719000	-1.473240000
O	-3.201397000	-2.837428000	0.000000000
C	1.209901000	-2.313446000	-0.696046000
C	1.209901000	-2.313446000	0.696047000
C	-5.839437000	0.189438000	0.710078000
C	2.677623000	-2.347597000	-1.073912000
C	-5.839437000	0.189438000	-0.710080000
C	3.281008000	-0.945606000	-0.783637000
C	3.281008000	-0.945606000	0.783637000
C	2.677623000	-2.347597000	1.073913000
O	3.235318000	-3.120537000	0.000000000
O	-7.026063000	2.018505000	0.000000000
C	-6.158313000	1.636320000	1.075178000
C	-4.900169000	2.436336000	0.743395000
C	-4.900168000	2.436337000	-0.743394000
C	-6.158313000	1.636321000	-1.075178000
C	-4.036036000	2.981879000	1.592248000
C	-4.036035000	2.981881000	-1.592246000
C	4.674450000	-0.777113000	-1.433889000
C	5.479883000	0.218115000	-0.666847000
C	5.479883000	0.218115000	0.666847000
C	4.674450000	-0.777112000	1.433889000
C	6.138209000	1.530110000	-1.073486000
C	5.089386000	2.599690000	-0.744371000
C	5.089386000	2.599690000	0.744370000
C	6.138209000	1.530110000	1.073486000
C	4.380345000	3.331322000	-1.595681000
C	4.380344000	3.331322000	1.595680000

O	7.067052000	1.728423000	0.000000000
H	-2.919838000	-2.470679000	2.040642000
H	-2.919838000	-2.470680000	-2.040642000
H	-2.906222000	0.204884000	-1.306876000
H	-2.906222000	0.204885000	1.306875000
H	0.032210000	-2.228361000	2.520755000
H	0.032210000	-2.228362000	-2.520755000
H	-5.112605000	-0.627035000	2.542014000
H	-5.187329000	-1.787101000	1.109779000
H	-5.187329000	-1.787100000	-1.109782000
H	-5.112604000	-0.627033000	-2.542017000
H	2.937458000	-2.782296000	-2.037051000
H	2.629623000	-0.153988000	-1.157256000
H	2.629623000	-0.153987000	1.157256000
H	2.937458000	-2.782295000	2.037052000
H	-6.612886000	1.825610000	2.043445000
H	-6.612885000	1.825612000	-2.043446000
H	-3.163502000	3.522842000	1.242196000
H	-4.181860000	2.909926000	2.664212000
H	-4.181857000	2.909928000	-2.664210000
H	-3.163500000	3.522843000	-1.242192000
H	5.190993000	-1.743898000	-1.412693000
H	4.562101000	-0.494345000	-2.483186000
H	4.562101000	-0.494344000	2.483186000
H	5.190993000	-1.743897000	1.412694000
H	6.620751000	1.604569000	-2.044082000
H	6.620751000	1.604570000	2.044082000
H	3.650201000	4.054639000	-1.249114000
H	4.517209000	3.234406000	-2.666787000
H	4.517209000	3.234407000	2.666786000
H	3.650201000	4.054639000	1.249112000

74

6a

C	2.644940000	-2.487448000	-1.074403000
C	1.177367000	-2.535449000	-0.698379000
C	1.177367000	-2.535449000	0.698379000
C	2.644940000	-2.487448000	1.074403000
C	3.164858000	-1.053041000	0.782707000
C	3.164858000	-1.053041000	-0.782707000
C	0.000000000	-2.513053000	-1.435061000
C	0.000000000	-2.513053000	1.435061000
C	4.550063000	-0.804018000	-1.411532000
C	4.550063000	-0.804018000	1.411532000
O	3.243183000	-3.229305000	0.000000000
C	-1.177367000	-2.535449000	0.698379000
C	-1.177367000	-2.535449000	-0.698379000
C	5.285245000	0.269842000	-0.666106000
C	-2.644940000	-2.487448000	1.074403000
C	5.285245000	0.269842000	0.666106000
C	-3.164858000	-1.053041000	0.782707000
C	-3.164858000	-1.053041000	-0.782707000
C	-2.644940000	-2.487448000	-1.074403000
O	-3.243183000	-3.229305000	0.000000000
C	7.387322000	1.571306000	0.665031000
C	5.999534000	1.461928000	-1.281578000
C	5.233159000	2.680988000	-0.744186000
C	5.233159000	2.680988000	0.744186000
C	5.999534000	1.461928000	1.281578000
C	4.656614000	3.584943000	-1.533213000
C	4.656614000	3.584943000	1.533213000
C	7.387322000	1.571306000	-0.665031000
C	-4.550063000	-0.804018000	1.411532000
C	-5.285245000	0.269842000	0.666106000
C	-5.285245000	0.269842000	-0.666106000
C	-4.550063000	-0.804018000	-1.411532000
C	-5.999534000	1.461928000	1.281578000
C	-5.233159000	2.680988000	0.744186000
C	-5.233159000	2.680988000	-0.744186000
C	-5.999534000	1.461928000	-1.281578000
C	-4.656614000	3.584943000	1.533213000
C	-4.656614000	3.584943000	-1.533213000
C	-7.387322000	1.571306000	0.665031000
C	-7.387322000	1.571306000	-0.665031000
H	2.929924000	-2.906708000	-2.037267000
H	2.929924000	-2.906708000	2.037267000
H	2.469931000	-0.301582000	1.160616000

H	2.469931000	-0.301582000	-1.160616000
H	0.000000000	-2.498633000	-2.519725000
H	0.000000000	-2.498633000	2.519725000
H	4.435287000	-0.551272000	-2.468641000
H	5.127820000	-1.735872000	-1.362604000
H	5.127820000	-1.735872000	1.362604000
H	4.435287000	-0.551272000	2.468641000
H	-2.929924000	-2.906708000	2.037267000
H	-2.469931000	-0.301582000	1.160616000
H	-2.469931000	-0.301582000	-1.160616000
H	-2.929924000	-2.906708000	-2.037267000
H	8.268089000	1.686640000	1.283857000
H	6.007460000	1.446328000	-2.370158000
H	6.007460000	1.446328000	2.370158000
H	4.113859000	4.432008000	-1.128882000
H	4.714496000	3.501851000	-2.612274000
H	4.714496000	3.501851000	2.612274000
H	4.113859000	4.432008000	1.128882000
H	8.268089000	1.686640000	-1.283857000
H	-5.127820000	-1.735872000	1.362604000
H	-4.435287000	-0.551272000	2.468641000
H	-5.127820000	-1.735872000	-1.362604000
H	-4.435287000	-0.551272000	-2.468641000
H	-6.007460000	1.446328000	2.370158000
H	-6.007460000	1.446328000	-2.370158000
H	-4.113859000	4.432008000	1.128882000
H	-4.714496000	3.501851000	2.612274000
H	-4.714496000	3.501851000	-2.612274000
H	-4.113859000	4.432008000	-1.128882000
H	-8.268089000	1.686640000	1.283857000
H	-8.268089000	1.686640000	-1.283857000

74

TS-**4a**-**6a**

C	-2.604456000	-2.453609000	1.073962000
C	-1.136629000	-2.430510000	0.696075000
C	-1.136629000	-2.430510000	-0.696075000
C	-2.604456000	-2.453609000	-1.073962000
C	-3.193491000	-1.045659000	-0.782673000
C	-3.193491000	-1.045659000	0.782673000
C	0.042492000	-2.347241000	1.435783000
C	0.042492000	-2.347241000	-1.435783000
C	-4.588082000	-0.864065000	1.413466000
C	-4.588082000	-0.864065000	-1.413466000
O	-3.167791000	-3.222789000	0.000000000
C	1.212967000	-2.290741000	-0.700392000
C	1.212967000	-2.290741000	0.700392000
C	-5.380837000	0.166618000	0.666064000
C	2.694556000	-2.190027000	-1.072068000
C	-5.380837000	0.166618000	-0.666064000
C	3.153066000	-0.772863000	-0.689192000
C	3.153066000	-0.772863000	0.689192000
C	2.694556000	-2.190027000	1.072068000
O	3.278523000	-2.946903000	0.000000000
C	-7.563928000	1.326803000	-0.665032000
C	-6.171650000	1.309432000	1.281415000
C	-5.488875000	2.577106000	0.744179000
C	-5.488875000	2.577106000	-0.744179000
C	-6.171650000	1.309432000	-1.281415000
C	-4.975734000	3.518595000	1.533091000
C	-4.975734000	3.518595000	-1.533091000
C	-7.563928000	1.326803000	0.665032000
C	5.317984000	-0.868542000	-1.439240000
C	5.886306000	0.137997000	-0.710431000
C	5.886306000	0.137997000	0.710431000
C	5.317984000	-0.868542000	1.439240000
C	6.167372000	1.524486000	-1.284495000
C	5.035928000	2.403328000	-0.743862000
C	5.035928000	2.403328000	0.743862000
C	6.167372000	1.524486000	1.284495000
C	4.169533000	3.038155000	-1.531688000
C	4.169533000	3.038155000	1.531688000
C	7.450677000	2.042215000	-0.665538000
C	7.450677000	2.042215000	0.665538000
H	-2.868424000	-2.885854000	2.037145000
H	-2.868424000	-2.885854000	-2.037145000

H	-2.535121000	-0.261729000	-1.160006000
H	-2.535121000	-0.261729000	1.160006000
H	0.041496000	-2.343906000	2.520499000
H	0.041496000	-2.343906000	-2.520499000
H	-4.483727000	-0.600663000	2.469128000
H	-5.117985000	-1.824307000	1.371211000
H	-5.117985000	-1.824307000	-1.371211000
H	-4.483727000	-0.600663000	-2.469128000
H	2.996410000	-2.581025000	-2.040472000
H	2.967649000	0.093045000	-1.308522000
H	2.967649000	0.093045000	1.308522000
H	2.996410000	-2.581025000	2.040472000
H	-8.450278000	1.383977000	-1.283989000
H	-6.178555000	1.293321000	2.369985000
H	-6.178555000	1.293321000	-2.369985000
H	-4.492177000	4.400692000	1.128524000
H	-5.027952000	3.432104000	2.612176000
H	-5.027952000	3.432104000	-2.612176000
H	-4.492177000	4.400692000	-1.128524000
H	-8.450278000	1.383977000	1.283989000
H	5.273193000	-1.877187000	-1.047664000
H	5.205136000	-0.765161000	-2.514202000
H	5.273193000	-1.877187000	1.047664000
H	5.205136000	-0.765161000	2.514202000
H	6.180875000	1.522035000	-2.372965000
H	6.180875000	1.522035000	2.372965000
H	3.365017000	3.642363000	-1.127556000
H	4.247032000	2.972966000	-2.610952000
H	4.247032000	2.972965000	2.610952000
H	3.365017000	3.642363000	1.127556000
H	8.271292000	2.389238000	-1.280667000
H	8.271292000	2.389238000	1.280667000

74

6b

C	2.822591000	-2.378805000	-1.074389000
C	1.354212000	-2.372535000	-0.698421000
C	1.354238000	-2.372534000	0.698445000
C	2.822627000	-2.378818000	1.074365000
C	3.396168000	-0.964985000	0.782714000
C	3.396133000	-0.964970000	-0.782736000
C	0.178383000	-2.309205000	-1.435088000
C	0.178437000	-2.309181000	1.435154000
C	4.788685000	-0.768723000	-1.413639000
C	4.788748000	-0.768770000	1.413561000
O	3.392695000	-3.142549000	-0.000026000
C	-0.999114000	-2.291267000	0.698407000
C	-0.999143000	-2.291289000	-0.698299000
C	5.570815000	0.269855000	-0.666099000
C	-2.464186000	-2.194956000	1.074409000
C	5.570848000	0.269830000	0.666022000
C	-2.935422000	-0.743191000	0.783302000
C	-2.935552000	-0.743303000	-0.783187000
C	-2.464231000	-2.195032000	-1.074248000
O	-3.086485000	-2.916085000	0.000129000
C	7.743897000	1.448362000	0.664960000
C	6.351736000	1.419424000	-1.281463000
C	5.658451000	2.681332000	-0.744166000
C	5.658490000	2.681303000	0.744182000
C	6.351800000	1.419373000	1.281393000
C	5.137447000	3.618506000	-1.533059000
C	5.137533000	3.618448000	1.533140000
C	7.743864000	1.448389000	-0.665098000
C	-4.306823000	-0.446330000	-1.416793000
C	-5.016298000	0.641318000	-0.666209000
C	-5.016213000	0.641606000	0.666264000
C	-4.306526000	-0.445723000	1.417185000
C	-5.909904000	1.705956000	-1.281280000
C	-7.313358000	1.382407000	-0.744046000
C	-7.313241000	1.382779000	0.744114000
C	-5.909688000	1.706547000	1.280989000
C	-8.355552000	1.131816000	-1.533272000
C	-8.355331000	1.132640000	1.533620000
C	-5.557994000	3.052501000	-0.665495000
C	-5.557884000	3.052809000	0.664538000
H	3.091728000	-2.808358000	-2.037252000

H	3.091792000	-2.808386000	2.037213000
H	2.729608000	-0.188019000	1.160111000
H	2.729547000	-0.188006000	-1.160087000
H	0.178857000	-2.295590000	-2.519766000
H	0.178949000	-2.295547000	2.519831000
H	4.681406000	-0.506209000	-2.469210000
H	5.328425000	-1.723479000	-1.371635000
H	5.328479000	-1.723529000	1.371498000
H	4.681518000	-0.506293000	2.469146000
H	-2.763064000	-2.604315000	2.037273000
H	-2.211835000	-0.018179000	1.158914000
H	-2.212292000	-0.018086000	-1.159066000
H	-2.763161000	-2.604473000	-2.037060000
H	8.629739000	1.513078000	1.283895000
H	6.358733000	1.403376000	-2.370032000
H	6.358851000	1.403281000	2.369961000
H	4.646626000	4.496575000	-1.128485000
H	5.190390000	3.532479000	-2.612145000
H	5.190533000	3.532377000	2.612220000
H	4.646695000	4.496536000	1.128629000
H	8.629676000	1.513129000	-1.284073000
H	-4.915636000	-1.359546000	-1.381004000
H	-4.177116000	-0.188108000	-2.471063000
H	-4.915453000	-1.358905000	1.382164000
H	-4.176441000	-0.186946000	2.471278000
H	-5.892400000	1.707879000	-2.369940000
H	-5.892026000	1.708956000	2.369645000
H	-9.333817000	0.896472000	-1.129321000
H	-8.256914000	1.155615000	-2.612334000
H	-8.256532000	1.156966000	2.612655000
H	-9.333672000	0.897171000	1.129927000
H	-5.376471000	3.921526000	-1.285021000
H	-5.376264000	3.922118000	1.283636000

74
TS-**4a-6b**

C	2.896811000	-2.347694000	-1.074129000
C	1.428827000	-2.342715000	-0.696243000
C	1.428849000	-2.342855000	0.695964000
C	2.896851000	-2.347889000	1.073791000
C	3.469298000	-0.933096000	0.782635000
C	3.469289000	-0.932966000	-0.782746000
C	0.248704000	-2.276704000	-1.435951000
C	0.248746000	-2.277019000	1.435723000
C	4.861474000	-0.735783000	-1.414024000
C	4.861482000	-0.735962000	1.413933000
O	3.469318000	-3.110176000	-0.000248000
C	-0.922487000	-2.238066000	0.700288000
C	-0.922504000	-2.237898000	-0.700471000
C	5.644622000	0.301728000	-0.666030000
C	-2.405424000	-2.163301000	1.072026000
C	5.644624000	0.301644000	0.666061000
C	-2.889532000	-0.754157000	0.690182000
C	-2.889555000	-0.753994000	-0.689947000
C	-2.405441000	-2.163034000	-1.072163000
O	-2.976420000	-2.929342000	-0.000163000
C	7.821520000	1.473130000	0.665111000
C	6.429257000	1.448828000	-1.281306000
C	5.740312000	2.713064000	-0.744011000
C	5.740291000	2.712958000	0.744336000
C	6.429264000	1.448669000	1.281472000
C	5.222814000	3.652217000	-1.532861000
C	5.222690000	3.651955000	1.533304000
C	7.821517000	1.473212000	-0.664950000
C	-5.043667000	-0.896885000	-1.438425000
C	-5.630108000	0.099937000	-0.709207000
C	-5.630342000	0.099695000	0.709134000
C	-5.044130000	-0.897303000	1.438277000
C	-5.995560000	1.463080000	-1.284249000
C	-7.387451000	1.794202000	-0.743933000
C	-7.387820000	1.793637000	0.743756000
C	-5.996079000	1.462611000	1.284536000
C	-8.431398000	2.042196000	-1.531872000
C	-8.432354000	2.040259000	1.531349000
C	-5.052105000	2.485652000	-0.665201000
C	-5.052399000	2.485425000	0.666280000

H	3.165829000	-2.776676000	-2.037364000
H	3.165915000	-2.777037000	2.036939000
H	2.801837000	-0.156926000	1.160028000
H	2.801855000	-0.156709000	-1.160008000
H	0.249584000	-2.274143000	-2.520665000
H	0.249661000	-2.274695000	2.520437000
H	4.753656000	-0.472047000	-2.469260000
H	5.401320000	-1.690569000	-1.373525000
H	5.401342000	-1.690737000	1.373329000
H	4.753662000	-0.472345000	2.469199000
H	-2.700579000	-2.560190000	2.040088000
H	-2.704321000	0.111313000	1.309165000
H	-2.704326000	0.111657000	-1.308681000
H	-2.700610000	-2.559668000	-2.040327000
H	8.707547000	1.534923000	1.284082000
H	6.436216000	1.432789000	-2.369879000
H	6.436230000	1.432498000	2.370043000
H	4.735162000	4.532027000	-1.128235000
H	5.275541000	3.566090000	-2.611950000
H	5.275388000	3.565680000	2.612382000
H	4.734977000	4.531781000	1.128785000
H	8.707540000	1.535085000	-1.283919000
H	-4.974872000	-1.902942000	-1.044074000
H	-4.947600000	-0.797742000	-2.515408000
H	-4.975065000	-1.903226000	1.043641000
H	-4.948277000	-0.798406000	2.515299000
H	-5.981807000	1.466282000	-2.372737000
H	-5.982784000	1.465387000	2.373031000
H	-9.409742000	2.276474000	-1.127987000
H	-8.334182000	2.014019000	-2.610912000
H	-8.335667000	2.011311000	2.610415000
H	-9.410687000	2.274039000	1.127150000
H	-4.469341000	3.159312000	-1.281172000
H	-4.469901000	3.158870000	1.282738000

91

11a

C	3.849830000	4.261648000	1.533078000
C	-5.536554000	5.123356000	1.070735000
C	-5.247906000	2.475134000	1.402723000
C	-4.971865000	-0.012992000	1.509728000
C	-4.646411000	-2.632869000	1.266326000
C	-2.200533000	-3.775852000	1.422168000
C	0.258691000	-4.416264000	1.074263000
C	2.622933000	-3.229750000	1.434954000
C	4.960501000	-1.991430000	1.074418000
C	5.874854000	0.383195000	1.408658000
C	6.060429000	3.067701000	1.281796000
C	4.795762000	3.756578000	0.744253000
C	-4.215494000	5.800255000	0.664298000
C	-5.386578000	3.642781000	0.700986000
C	-5.109987000	1.257989000	0.696294000
C	-4.822092000	-1.230693000	0.662435000
C	-3.349547000	-3.182740000	0.664077000
C	-0.857310000	-3.375266000	0.783186000
C	1.586770000	-3.789714000	0.698382000
C	3.679412000	-2.709265000	0.698398000
C	4.760509000	-0.478960000	0.782674000
C	6.016457000	1.678814000	0.666195000
C	4.795776000	3.756601000	-0.744238000
C	-4.215554000	5.800288000	-0.664388000
C	-5.386597000	3.642779000	-0.700978000
C	-5.110003000	1.257987000	-0.696282000
C	-4.822103000	-1.230696000	-0.662418000
C	-3.349547000	-3.182728000	-0.664068000
C	-0.857314000	-3.375261000	-0.783197000
C	1.586767000	-3.789707000	-0.698405000
C	3.679410000	-2.709258000	-0.698417000
C	4.760503000	-0.478953000	-0.782672000
C	6.016452000	1.678819000	-0.666187000
C	3.849865000	4.261706000	-1.533064000
C	-5.536620000	5.123351000	-1.070734000
C	-5.247941000	2.475129000	-1.402712000
C	-4.971885000	-0.012997000	-1.509713000
C	-4.646423000	-2.632870000	-1.266306000
C	-2.200543000	-3.775839000	-1.422173000

C	0.258687000	-4.416254000	-1.074288000
C	2.622928000	-3.229736000	-1.434975000
C	4.960497000	-1.991418000	-1.074433000
C	5.874844000	0.383206000	-1.408658000
C	6.060437000	3.067709000	-1.281779000
O	5.832841000	-2.374718000	-0.000011000
O	0.064578000	-5.349054000	-0.000017000
C	7.217990000	3.840152000	0.665033000
C	7.218003000	3.840142000	-0.665006000
O	-6.390365000	5.554777000	0.000026000
C	-5.778313000	-3.481468000	-0.663480000
C	-5.778311000	-3.481457000	0.663516000
H	2.961733000	4.734033000	1.128417000
H	3.940733000	4.217647000	2.612178000
H	-5.957471000	5.363735000	2.042014000
H	-5.248932000	2.461727000	2.488888000
H	-4.110007000	0.080915000	2.183999000
H	-5.846465000	-0.120230000	2.165342000
H	-4.645845000	-2.626654000	2.356070000
H	-2.285146000	-4.870814000	1.403084000
H	-2.210733000	-3.473397000	2.472852000
H	0.196086000	-4.919379000	2.037069000
H	2.616278000	-3.216751000	2.519636000
H	5.406731000	-2.232356000	2.037202000
H	6.819779000	-0.171819000	1.350036000
H	5.663009000	0.548776000	2.467971000
H	6.075096000	3.058105000	2.370349000
H	-3.441032000	6.116667000	1.347478000
H	-0.574281000	-2.390001000	1.156811000
H	3.797610000	-0.132358000	1.161168000
H	-3.441151000	6.116725000	-1.347623000
H	-0.574287000	-2.389993000	-1.156816000
H	3.797600000	-0.132349000	-1.161157000
H	3.940781000	4.217725000	-2.612164000
H	2.961769000	4.734093000	-1.128405000
H	-5.957604000	5.363714000	-2.041987000
H	-5.249006000	2.461716000	-2.488877000
H	-5.846486000	-0.120240000	-2.165325000
H	-4.110029000	0.080913000	-2.183985000
H	-4.645869000	-2.626662000	-2.356050000
H	-2.210750000	-3.473383000	-2.472855000
H	-2.285159000	-4.870801000	-1.403088000
H	0.196079000	-4.919361000	-2.037099000
H	2.616269000	-3.216725000	-2.519657000
H	5.406724000	-2.232334000	-2.037221000
H	5.662991000	0.548795000	-2.467968000
H	6.819769000	-0.171809000	-1.350046000
H	6.075110000	3.058121000	-2.370332000
H	7.931225000	4.369726000	1.283771000
H	7.931247000	4.369713000	-1.283737000
H	-6.485033000	-4.004415000	-1.294130000
H	-6.485033000	-4.004372000	1.294189000

91

12

C	3.848204000	-2.529823000	-1.400247000
C	1.692423000	-3.907919000	-1.076240000
C	-0.907240000	-3.404586000	-1.402118000
C	-3.335682000	-2.768308000	-1.492760000
C	-5.441820000	-1.143745000	-1.268442000
C	-4.708708000	1.465755000	-1.390922000
C	-3.025790000	3.397745000	-1.074759000
C	-0.394489000	3.662656000	-1.434199000
C	2.249360000	3.726897000	-1.074639000
C	4.159803000	2.027927000	-1.402589000
C	5.467357000	-0.351139000	-1.272296000
C	4.678743000	-1.520482000	-0.664080000
C	2.434524000	-2.575610000	-0.783088000
C	0.239285000	-3.700298000	-0.698706000
C	-2.089809000	-3.114548000	-0.699465000
C	-4.337934000	-2.033286000	-0.662727000
C	-5.185228000	0.242418000	-0.664480000
C	-3.360074000	1.909882000	-0.782647000
C	-1.571580000	3.608100000	-0.698428000
C	0.780537000	3.752691000	-0.698393000
C	2.772000000	2.293445000	-0.782936000

C	4.831349000	0.908286000	-0.664036000
C	4.678739000	-1.520480000	0.664086000
C	2.434520000	-2.575610000	0.783088000
C	0.239283000	-3.700303000	0.698700000
C	-2.089812000	-3.114555000	0.699457000
C	-4.337938000	-2.033294000	0.662723000
C	-5.185220000	0.242415000	0.664487000
C	-3.360067000	1.909880000	0.782649000
C	-1.571578000	3.608105000	0.698428000
C	0.780539000	3.752696000	0.698388000
C	2.772000000	2.293448000	0.782933000
C	4.831349000	0.908288000	0.664038000
C	3.848198000	-2.529820000	1.400252000
C	1.692421000	-3.907921000	1.076237000
C	-0.907245000	-3.404599000	1.402112000
C	-3.335688000	-2.768324000	1.492752000
C	-5.441818000	-1.143748000	1.268445000
C	-4.708695000	1.465747000	1.390933000
C	-3.025787000	3.397744000	1.074763000
C	-0.394486000	3.662667000	1.434196000
C	2.249362000	3.726902000	1.074632000
C	4.159803000	2.027931000	1.402587000
C	5.467353000	-0.351137000	1.272302000
O	2.840681000	4.472393000	-0.000006000
O	-3.706482000	4.062472000	0.000004000
C	6.875120000	-0.439059000	-0.663279000
C	6.875118000	-0.439062000	0.663289000
O	2.155909000	-4.738275000	-0.000001000
C	-6.759338000	-1.638803000	0.663396000
C	-6.759334000	-1.638813000	-0.663393000
H	1.906785000	-4.369733000	-2.037861000
H	-0.911705000	-3.376457000	-2.488025000
H	-3.049490000	-2.161718000	-2.359775000
H	-3.780345000	-3.684669000	-1.904622000
H	-5.438090000	-1.147117000	-2.357926000
H	-5.430529000	2.282716000	-1.270905000
H	-4.593712000	1.287373000	-2.462838000
H	-3.359517000	3.780487000	-2.037129000
H	-0.392826000	3.636236000	-2.518768000
H	2.531917000	4.148536000	-2.037133000
H	4.759787000	2.943092000	-1.318963000
H	4.053298000	1.807452000	-2.467799000
H	5.467066000	-0.350977000	-2.361593000
H	-2.574892000	1.253841000	-1.163431000
H	2.074541000	1.543741000	-1.161148000
H	-2.574881000	1.253839000	1.163425000
H	2.074541000	1.543746000	1.161148000
H	3.779666000	-2.301754000	2.466931000
H	4.300585000	-3.525393000	1.308981000
H	1.906782000	-4.369735000	2.037858000
H	-0.911713000	-3.376478000	2.488019000
H	-3.780352000	-3.684689000	1.904605000
H	-3.049498000	-2.161742000	2.359773000
H	-5.438085000	-1.147124000	2.357928000
H	-4.593691000	1.287361000	2.462847000
H	-5.430518000	2.282709000	1.270925000
H	-3.359511000	3.780483000	2.037134000
H	-0.392821000	3.636253000	2.518766000
H	2.531921000	4.148545000	2.037123000
H	4.053297000	1.807459000	2.467798000
H	4.759787000	2.943096000	1.318958000
H	5.467059000	-0.350974000	2.361599000
H	7.753990000	-0.495102000	-1.291970000
H	7.753985000	-0.495107000	1.291983000
H	-7.586685000	-1.940460000	1.292131000
H	-7.586679000	-1.940477000	-1.292128000
H	3.779677000	-2.301759000	-2.466926000
H	1.863622000	-1.725472000	1.161208000
H	1.863628000	-1.725472000	-1.161212000
H	4.300590000	-3.525396000	-1.308973000

5. References

1. Zhao, Y.; Truhlar, D. *Theor. Chem. Acc.* **2008**, *120*, 215.
2. Becke, A. D. *J. Chem. Phys.* **1993**, *98*, 5648.
3. Lee, C.; Yang, W.; Parr, R. G. *Phys. Rev. B* **1988**, *37*, 785.
4. Grimme, S.; Antony, J.; Ehrlich, S.; Krieg, H. *J. Chem. Phys.* **2010**, *132*, 154104.
5. Grimme, S.; Ehrlich, S.; Goerigk, L. *J. Comp. Chem.* **2011**, *32*, 1456.
6. Riplinger, C.; Neese, F. *J. Chem. Phys.* **2013**, *138*, 034106.
7. Riplinger, C.; Sandhoefer, B.; Hansen, A.; Neese, F. *J. Chem. Phys.* **2013**, *139*, 134101.
8. Riplinger, C.; Pinski, P.; Becker, U.; Valeev, E. F.; Neese, F. *J. Chem. Phys.* **2016**, *144*, 024109.
9. Liakos, D. G.; Sparta, M.; Kesharwani, M. K.; Martin, J. M. L.; Neese, F. *J. Chem. Theor. Comput.* **2015**, *11*, 1525.
10. Dunning, T. H. *J. Chem. Phys.* **1989**, *90*, 1007.
11. Weigend, F.; Köhn, A.; Hättig, C. *J. Chem. Phys.* **2002**, *116*, 3175.
12. Liakos, D. G.; Guo, Y.; Neese, F. *J. Phys. Chem. A* **2020**, *124*, 90.
13. Tomasi, J.; Mennucci, B.; Cammi, R. *Chem. Rev.* **2005**, *105*, 2999.
14. Frisch, M. J.; Trucks, G. W.; Schlegel, H. B.; Scuseria, G. E.; Robb, M. A.; Cheeseman, J. R.; Scalmani, G.; Barone, V.; Petersson, G. A.; Nakatsuji, H.; Li, X.; Caricato, M.; Marenich, A. V.; Bloino, J.; Janesko, B. G.; Gomperts, R.; Mennucci, B.; Hratchian, H. P.; Ortiz, J. V.; Izmaylov, A. F.; Sonnenberg, J. L.; Williams; Ding, F.; Lipparini, F.; Egidi, F.; Goings, J.; Peng, B.; Petrone, A.; Henderson, T.; Ranasinghe, D.; Zakrzewski, V. G.; Gao, J.; Rega, N.; Zheng, G.; Liang, W.; Hada, M.; Ehara, M.; Toyota, K.; Fukuda, R.; Hasegawa, J.; Ishida, M.; Nakajima, T.; Honda, Y.; Kitao, O.; Nakai, H.; Vreven, T.; Throssell, K.; Montgomery Jr., J. A.; Peralta, J. E.; Ogliaro, F.; Bearpark, M. J.; Heyd, J. J.; Brothers, E. N.; Kudin, K. N.; Staroverov, V. N.; Keith, T. A.; Kobayashi, R.; Normand, J.; Raghavachari, K.; Rendell, A. P.; Burant, J. C.; Iyengar, S. S.; Tomasi, J.; Cossi, M.; Millam, J. M.; Klene, M.; Adamo, C.; Cammi, R.; Ochterski, J. W.; Martin, R. L.; Morokuma, K.; Farkas, O.; Foresman, J. B.; Fox, D. J., Gaussian 16 Rev. C.01, Wallingford, CT, 2016.
15. Neese, F. *WIREs Comput. Mol. Sci.* **2012**, *2*, 73.
16. Neese, F. *WIREs Computational Molecular Science* **2018**, *8*, e1327.
